# Carbamate-Functionalized N‑Acetyl-D-Glucosamine Derivatives for Gel Formation and Chemiluminescent Properties of their Metallogels

**DOI:** 10.1021/acsomega.6c05849

**Published:** 2026-09-16

**Authors:** Pooja Sharma, Pramod Aryal, Richard Chen, Alice Mao, Guijun Wang

**Affiliations:** Department of Chemistry and Biochemistry, 6042 Old Dominion University, Norfolk, Virginia 23529, United States

## Abstract

Sugar-based low-molecular-weight gelators are an interesting and useful class of compounds due to their many applications. Several carbamate derivatives of d-glucose and d-glucosamine have been previously reported as effective gelators, demonstrating that the fine-tuning of molecular structures can have a profound influence on molecular assembling properties. In this study, a series of 12 3-O-carbamate derivatives of N-acetyl-d-glucosamine were synthesized in short steps with several derivatives found to be organogelators. The derivatives were screened in 11 different solvents for their gelation properties. Among the series of synthesized compounds, ten derivatives showed gelation in at least one of the tested solvents. Selected gelators were characterized and then evaluated for their metallogel formation and metal ion sensing abilities. The most versatile gelator in the series is the cyclohexyl derivative, compound **7**, which formed gels in several solvents including DMSO/water and ethanol/water mixtures, as well as in the presence of various metal ions. The hydrogels formed by compound **7** in DMSO/water mixtures can be used for dye removal. In addition, the metallogels formed by the gelator demonstrated enhanced chemiluminescence for luminol and hydrogen peroxide.

## Graphical abstract

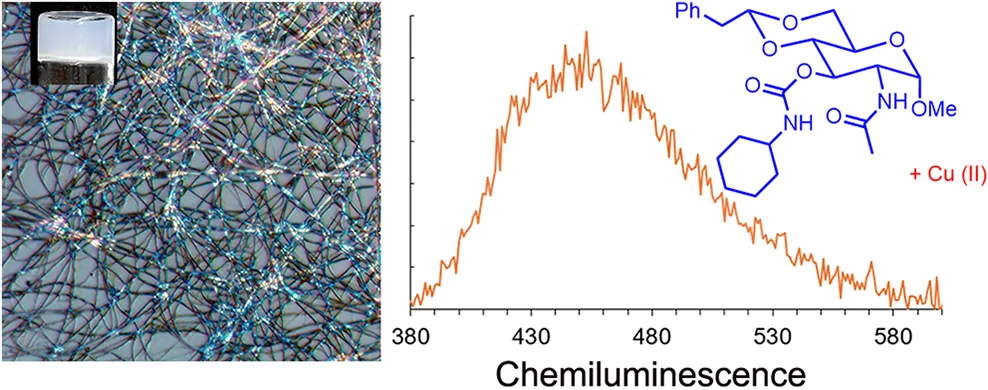

## Introduction

There has been growing interest and significant development in the field of low-molecular-weight gelators (LMWGs), also known as molecular gelators, over the past two decades.^1–5^ LMWGs are able to form thermally reversible gels in various solvents, and the resulting gels are often referred to as supramolecular gels. These gels have found applications in various fields, ranging from environmental pollution abatement, such as oil-spill remediation and dye removal,^6–8^ to biomedical applications, such as targeted drug delivery, as matrices for tissue engineering,^3,9–12^ and for the detection of ions and applications in catalysis.^13–16^ For these various applications, it is ideal for the gelator molecules to be nontoxic, biocompatible, readily available, and inexpensive. Carbohydrates are among the best candidates for the preparation of these self-assembling systems, and common sugars have been functionalized to obtain molecular gelators with multifarious applications.^2,17–20^ As part of our efforts in the discovery of functional carbohydrate-based LMWGs, we have identified that certain carbohydrate templates can be used for further functionalization to form supramolecular gels.^2^ Several classes of sugar derivatives were found to be effective gelators, and the gels have exhibited applications for phase-selective gelation, dye removal, and as drug delivery carriers.^21–24^ The incorporation of metal ions into supramolecular gels can introduce additional desired properties, making metallogels and other multi-component gels attractive soft materials for interdisciplinary applications.^25–34^ Certain metal-organic gels have also been explored for their catalytic properties in various reactions, while others have been able to form conductive multi-component gels.^35–39^

The development of highly efficient chemiluminescence (CL) catalysts is important for expanding the application of the luminol CL system in many fields, including drug analysis, clinical diagnosis, environmental testing, and food safety analysis.^40^ It is interesting to note that supramolecular hydrogels formed by molecular self-assembly have been shown to exhibit enhanced CL for luminol.^41^ Recently, researchers have developed metal-organic gels as enzyme-mimicking catalysts for CL enhancement and applied these systems to the quantitative measurement of dopamine in clinical samples.^42,43^ Some luminol imide derivatives have been synthesized and identified as organogelators that are useful for electrochemiluminescence (ECL) measurements.^44^ Copper-based metal-organic gels have been reported in the literature to exhibit CL for the luminol/H_2_O_2_ system and were demonstrated for dopamine (DA) detection.^45^ In another report, metal-organic frameworks formed by Fe^3+^ and Co^2+^ ions with terephthalic acid also showed CL and were used for DA analysis.^46^ FeCoNi trimetallic organo-xerogels were found to exhibit enhanced CL for luminol/hydrogen peroxide;^42^ the FeCoNi-MOXs have peroxidase-like functions and are able to catalyze the CL generation of luminol/H_2_O_2_. A Mn-porphyrin metal-organic framework likewise exhibited nanozyme-mimicking properties that enhanced luminol CL, and the resulting system was used for DA detection.^47^

Common monosaccharides such as d-glucose and d-glucosamine have been used as building blocks for the design and construction of molecular gelators; they have shown applications in environmental contexts.^22,23,48–52^ Selective functionalization of d-glucosamine at different positions has produced a series of carbamate derivatives that exhibit versatile gel-formation properties in many different solvents.^36,53^ The C-2 carbamate derivatives (**I**) were effective LMWGs, forming gels in the presence of various anions, and their metal-organic xerogels exhibited catalytic effect for luminol’s chemiluminescence. (Figure 1). This indicated that the introduction of a methylene group to the 4,6-acetal protection leads to derivatives that are useful in gelation in organic solvents and aqueous mixtures. The C-3 carbamate derivatives (**II**) were prepared and reported as effective LMWGs, forming gels in the presence of metal ions and various tetraalkylammonium ions.^53^ In this study, we aimed to understand the structural influence on gelation properties and obtain the general scope of effective gelators by further functionalization of the N-acetyl-d-glucosamine derivatives. Another series of C-3 carbamate derivatives with the general structure **III**, which contain the benzyl acetal protective group, were synthesized, and their gel-formation properties were evaluated. We envisioned that this small modification to the template would preserve the gelation formation properties and may lead to effective molecular self-assemblies with different properties compared to those of previous studies.

**Figure 1:**
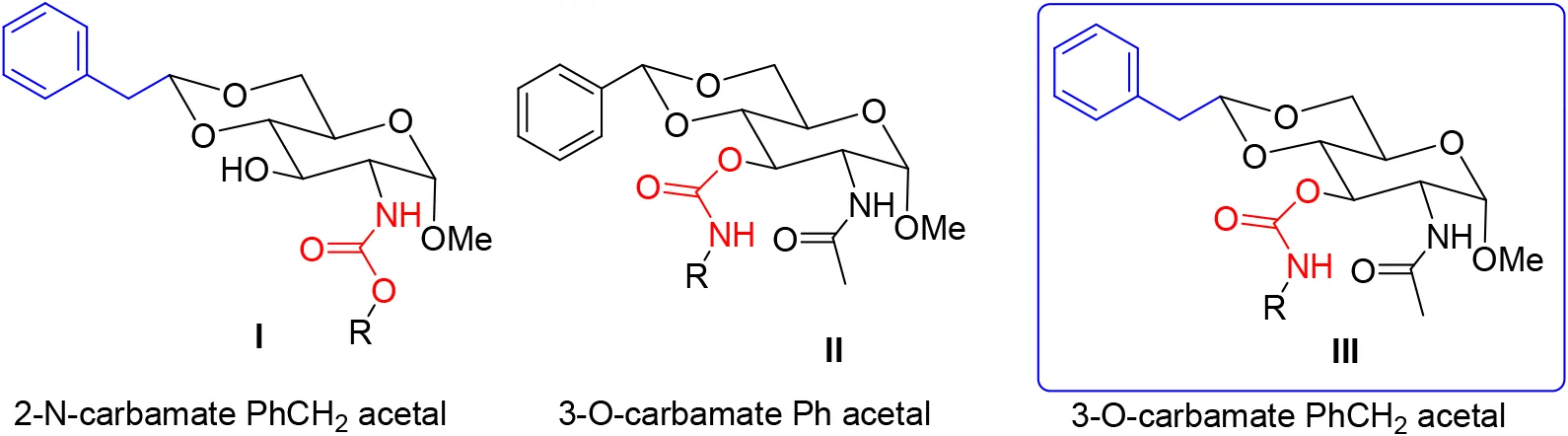
Structures of glucosamine-based gelators **I** and **II,** and the structure of carbamate derivatives **III** for this study.

## Results and Discussion

The synthesis of the series of carbamates is shown in Scheme 1. The 4,6-protected headgroup **1** was prepared from *N*-acetyl-d-glucosamine following a literature procedure.^24^ It was converted to the C-3 imidazole derivative **2** by treatment with carbonyldiimidazole (CDI).^53,54^ The compound was then reacted with various aliphatic amines to produce a series of O-linked carbamate derivatives **3–14** at the 3-position of the sugar ring. The selection of the amine library was based on the gelation properties of the C-3 carbamate reported previously, including short-chain alkyl amines and several benzyl amines and dimeric amines.

**Scheme 1:**
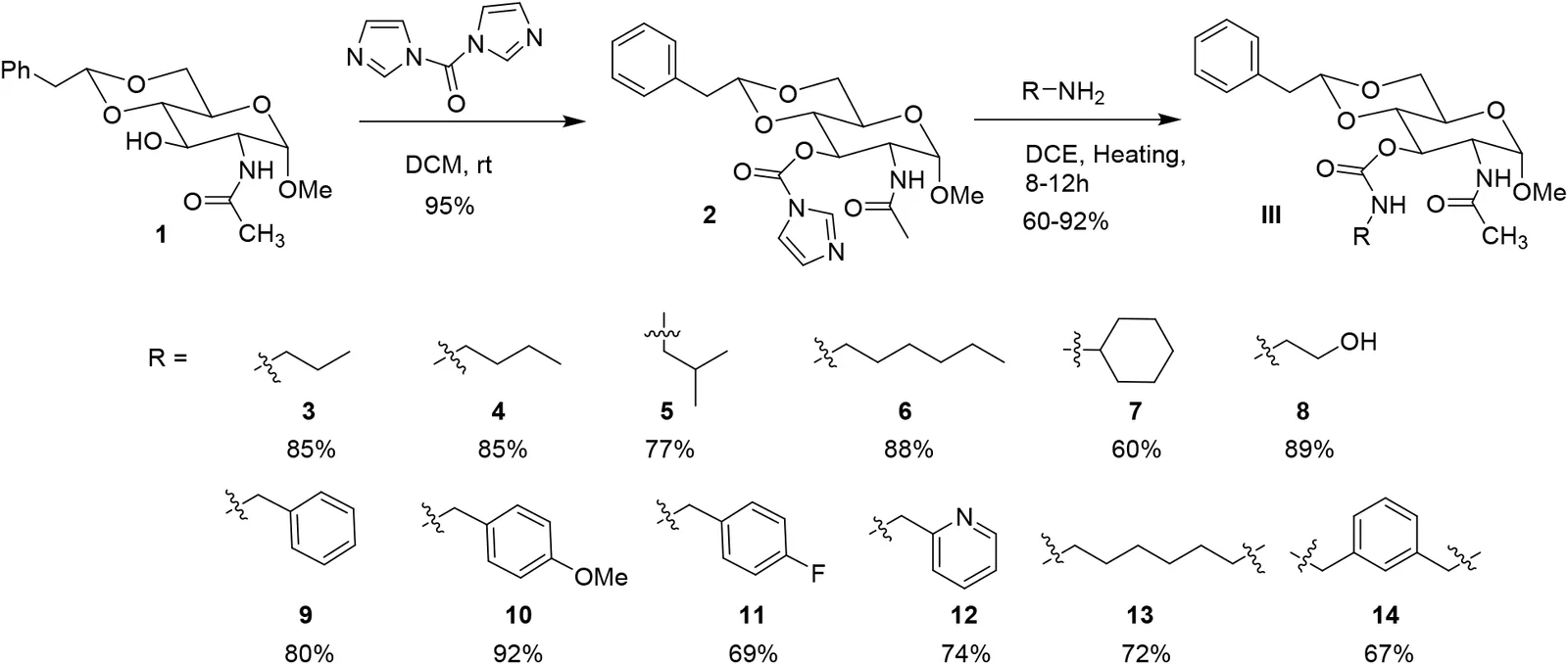
Synthesis of Carbamate Derivatives **3–14** with 4,6-*O*-(Phenylethylidene) Protected N-acetyl Glucosamine Head Group

After these compounds were synthesized, they were screened in a series of solvents for their self-assembly properties. Detailed results of the gelation properties are shown in Table 1. The solvents tested included hexane, toluene, glycerol, pump oil, ethanol–water mixture, DMSO–water mixture, and water. As shown in Table 1, several compounds exhibited gelation properties in multiple solvents. The aliphatic derivatives **3–7** and the aromatic derivatives **9–11** formed gels in at least three different solvents. Among these compounds, the benzyl derivative **9** exhibited the lowest minimum gelation concentration (MGC) in pump oil. Within the aliphatic carbamates, the branched isobutyl derivative **5** and the cyclohexyl derivative **7** were more effective than the linear-chain derivatives **3**, **4**, and **6**. Compounds **5** and **7** formed gels in almost all tested solvents except toluene. Most of the synthesized compounds formed gels in glycerol, vacuum pump oil, ethanol/water mixtures, and DMSO/water mixtures, typically at concentrations of 20.0 mg/mL. The cyclohexyl carbamate derivative **7** was the best-performing gelator among the class of compounds, forming gels in several solvents at low concentrations below 0.2 wt %. It formed stable gels in EtOH:H_2_O (v/v 1:2) and in DMSO:H_2_O (v/v 1:2) at 1.8 mg/mL. The compound was tested in different ratios of DMSO to water; at a 1:1 ratio, it formed a spontaneous gel (10 mg/mL), and at tested ratios of DMSO:H_2_O from 1:2 up to 1:10 (1.8 mg/mL), stable gels were formed after heating and cooling The resulting gels ranged from opaque to translucent, and representative photographs of these gels are provided in Figure S21 of the Supporting Information. Two aromatic derivatives from this series, the p-methoxyl benzyl carbamate **10** and the p-fluoro benzyl derivative **11**, formed gels in toluene at 20.0 mg/mL. In general, derivatives with aromatic functions displayed less extensive gelation in comparison to the aliphatic derivatives, only forming gels at 20.0 mg/mL in a limited number of solvents, including EtOH:H_2_O (v/v 1:1), DMSO:H_2_O (v/v 1:1 and 1:2), and glycerol. Interestingly, the two dimers synthesized using 1,6-diamino hexane and m-xylene diamine remained insoluble or precipitated in most solvents tested and therefore were not effective gelators. A possible reason for the enhanced performance of the aliphatic derivatives is due to the enhanced van der Waals interactions that the aliphatic chains can provide. In addition, the alkyl groups make the packing of the parent headgroups less rigid, thereby providing further interactions with solvents and immobilizing the solvents. The stronger aromatic group interactions led to being more prone to precipitation than gelation.

**Table 1:**
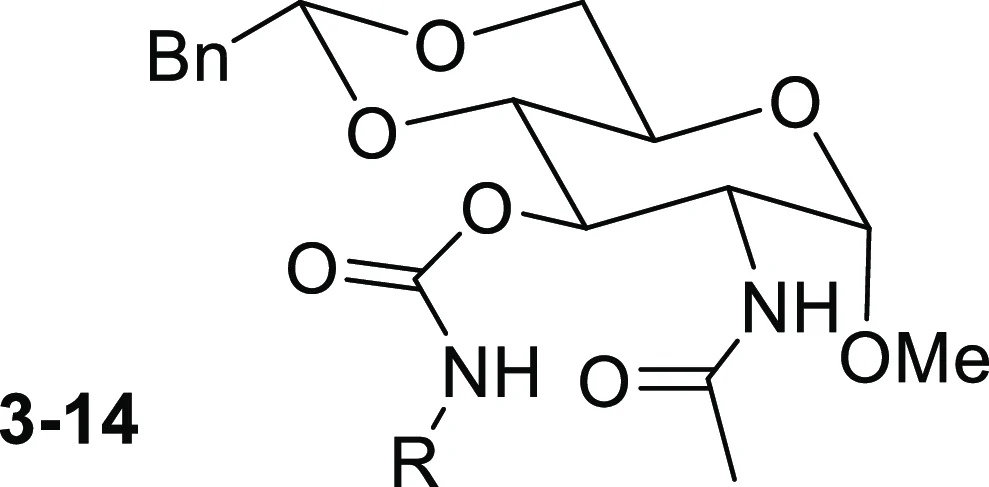

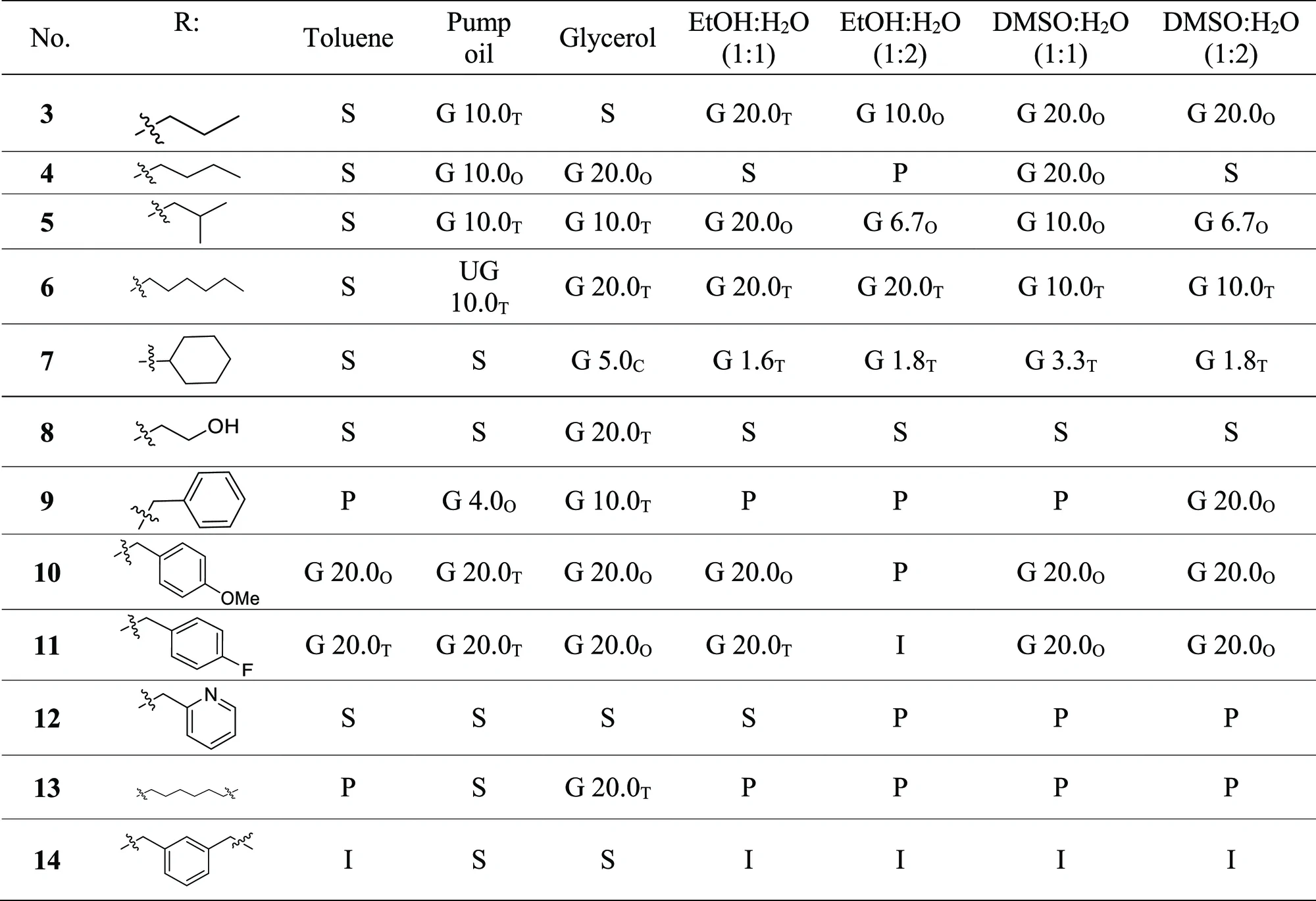
Gelation Test Results of Library of 3-Carbamates in Several Solvents

All compounds were tested starting at 20.0 mg/mL. The following abbreviations are used in Table 1: UG, unstable gel at room temperature; G, stable gel at room temperature; P, precipitation; S, soluble; I, insoluble. Numerical values indicate the minimum gelation concentration (MGC) in mg/mL; gel appearance: C, clear or transparent; T, translucent; O, opaque. At 20.0 mg/mL, all compounds were soluble in *i*-PrOH and EtOH but were insoluble or precipitated in hexane and water. The ratios of solvent mixtures are all in volume ratios.

To analyze the relationship between molecular structures and the gelation mechanism, the gelation properties of derivatives with general structure **III** in this study were compared with those of the corresponding derivative **II**, with a focus on the effect of introducing a methylene group at the 4,6-acetal position (**III**). As shown in Table 2, for hexyl carbamate derivatives, no significant difference in gelation performance was observed; compound **II-a** and compound **6** formed gels similarly in glycerol and aqueous mixtures. However, for cyclohexyl derivatives, in glycerol, ethanol water (1:2), and DMSO water (1:2), structure **III** was more effective than **II-b**; compound **7** formed gels in all three solvents and at a lower MGC for the aqueous solutions. The *p*-methoxy derivative in **III** (compound **10**) exhibited enhanced gelation properties. These comparisons suggest that the introduction of a methylene group did not diminish the gelation properties and, for certain analogues and solvent systems, can lead to more effective gelation. The reason for this may be related to the additional structural flexibility introduced by the aliphatic acetal relative to that of the more structurally rigid aromatic acetal.

**Table 2:**
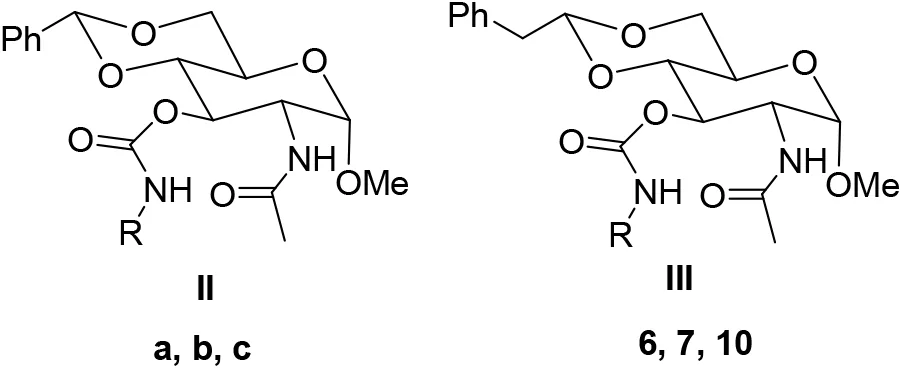

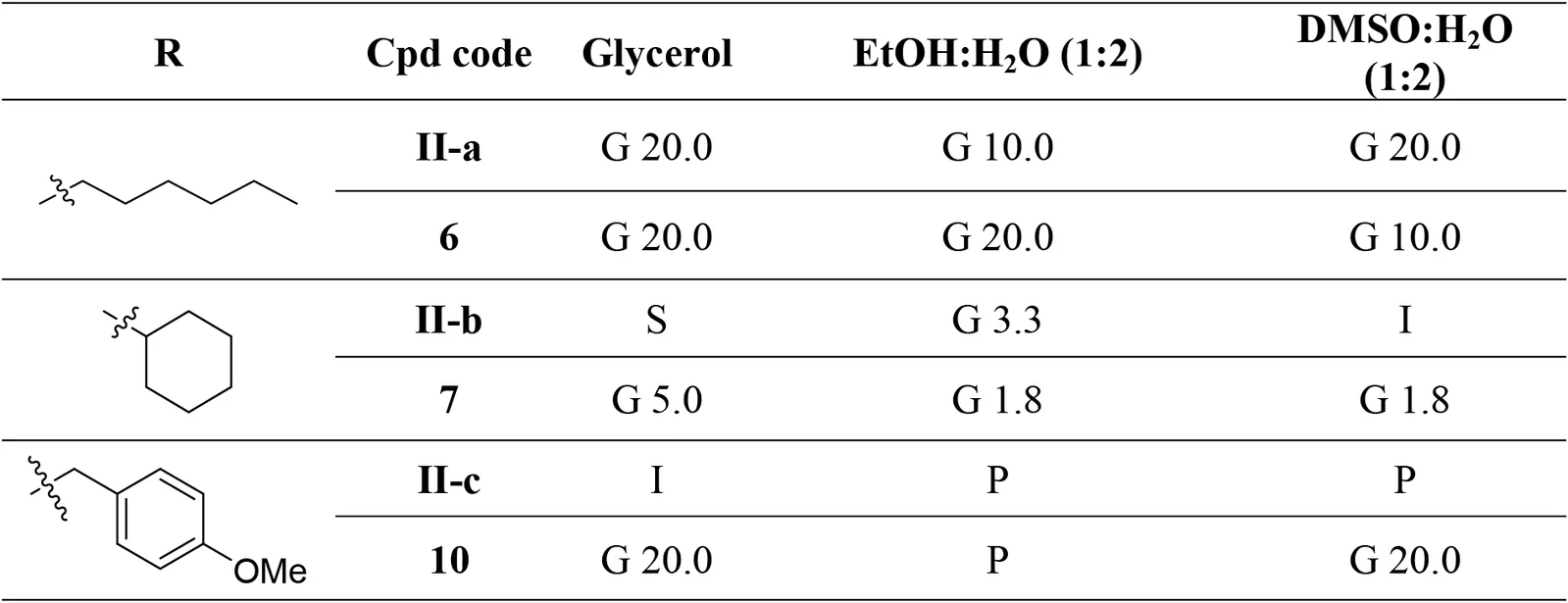
Gelation Property Comparisons for Compounds **II** and **III**

It is interesting to note that among the series of alkyl and aryl groups attached to the 3-OH via a carbamoyl function, the cyclohexyl derivative exhibited gelation properties in multiple solvents and formed gels at relatively low concentrations in several solvents. This “cyclohexyl effect” was noted by Miravet et al. who observed that linear n-hexyl derivative and cyclohexyl derivatives exhibited very different gelation properties.^55^ The cyclohexyl derivative formed gels while the n-hexyl derivative did not. This difference was attributed to the unique geometry and hydrophobicity of the cyclohexyl group compared to linear alkyl or aromatic groups. The gelation properties of compound **7** may be due to similar mechanisms. The bulkier cyclohexyl group may allow the formation of the molecular assembly in a preformed extended conformation that enhances the intermolecular hydrogen-bonding network, leading to more effective gelation. The cyclohexyl derivative **7** was able to form gels in aqueous solutions at low concentrations; this could be useful for further applications. This compound was then evaluated for its capacity to form gels in the presence of metal ions, since metallogels are interesting materials with numerous potential applications. Solutions of several metal salts were prepared in water, and 2.0 mg of compound **7** was dissolved in 0.5 mL of a DMSO:H_2_O (v/v 1:3) solution, one equivalent of metal salt (total 0.5 mL) was added to the solution, and the mixture was heated and cooled for gelation test. The compound was able to form gels in the presence of several metal salts including Zn­(OAc)_2_, Cu­(OAc)_2_, NiCl_2_, and FeCl_2_,but only precipipates formed with Hg­(OAc)_2_. Photographs of the gels formed by compound **7** in the presence of these metal salts are shown in Figure S24.

### Gel Morphology Characterization

Optical microscopy and scanning electron microscopy were used to characterize the morphological features of the selected gel samples. Liu et al. have shown that self-organized networks of LMWGs form nanofibrous networks with fractal-like patterns or spherulite structures.^56,57^ The molecular structures of gelator molecules can affect the structural features of their molecular assemblies, and the different aggregation morphologies may affect the gelation strength.^58,59^ Compounds **5** and **7** were studied in detail because they formed gels at relatively low concentrations compared with the other compounds. Their optical micrographs (OM) are shown in Figures 2–3, and the corresponding SEM images are shown in Figure 4. For compounds that formed gels at higher concentrations, such as 20 mg/mL, the aggregates typically formed planar sheets or crystalline tubes, an example is shown in Figure 2. The gel formed by compound **3** in EtOH:H_2_O (v/v 1:1) at 20 mg/mL formed straight tubules or cylinders (Figure 2a). The gel formed by compound **5** in EtOH:H_2_O (v/v 1:2) at 6.7 mg/mL displayed crystalline planar ribbons or stacked sheets (Figure 2b). Compound **7** in EtOH:H_2_O (v/v 1:1) at 1.6 mg/mL formed smooth films with densely packed thin fibers on top of the film (Figure 2c). The gel formed by compound **7** in DMSO:H_2_O (v/v 1:2) at 1.8 mg/mL exhibited very uniform and long fibers; these curved fibers were also somewhat intertwined but distinctive at the edge part of the gel (Figure 2d–e). The center of the dried gel appears to have more densely bundled fibers, and these were densely stacked on to one another. The gel formed by compound **10** in EtOH:H_2_O (v/v 1:1) at 20 mg/mL exhibited planar sheet morphologies (Figure 2f).

**Figure 2:**
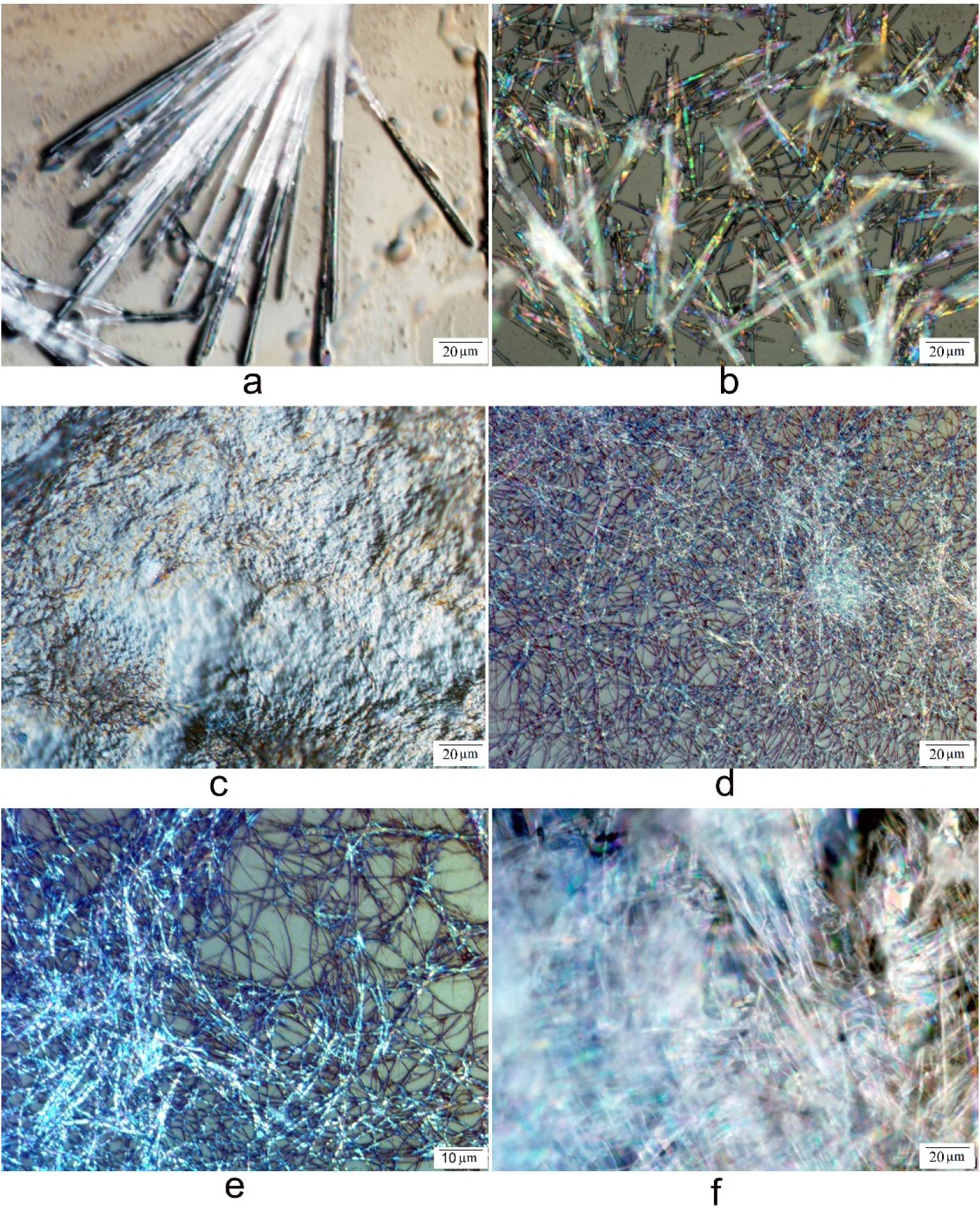
Optical micrographs of the gels formed by several compounds. (a) Compound **3** in EtOH:H_2_O (v/v 1:1) at 20 mg/mL; (b) compound **5** in EtOH:H_2_O (v/v 1:2) at 6.7 mg/mL; (c) compound **7** in EtOH:H_2_O (v/v 1:1) at 1.6 mg/mL; (d-e) compound **7** in DMSO:H_2_O (v/v 1:2) at 1.8 mg/mL; (f) compound **10** in EtOH:H_2_O (v/v 1:1) at 20 mg/mL.

**Figure 3:**
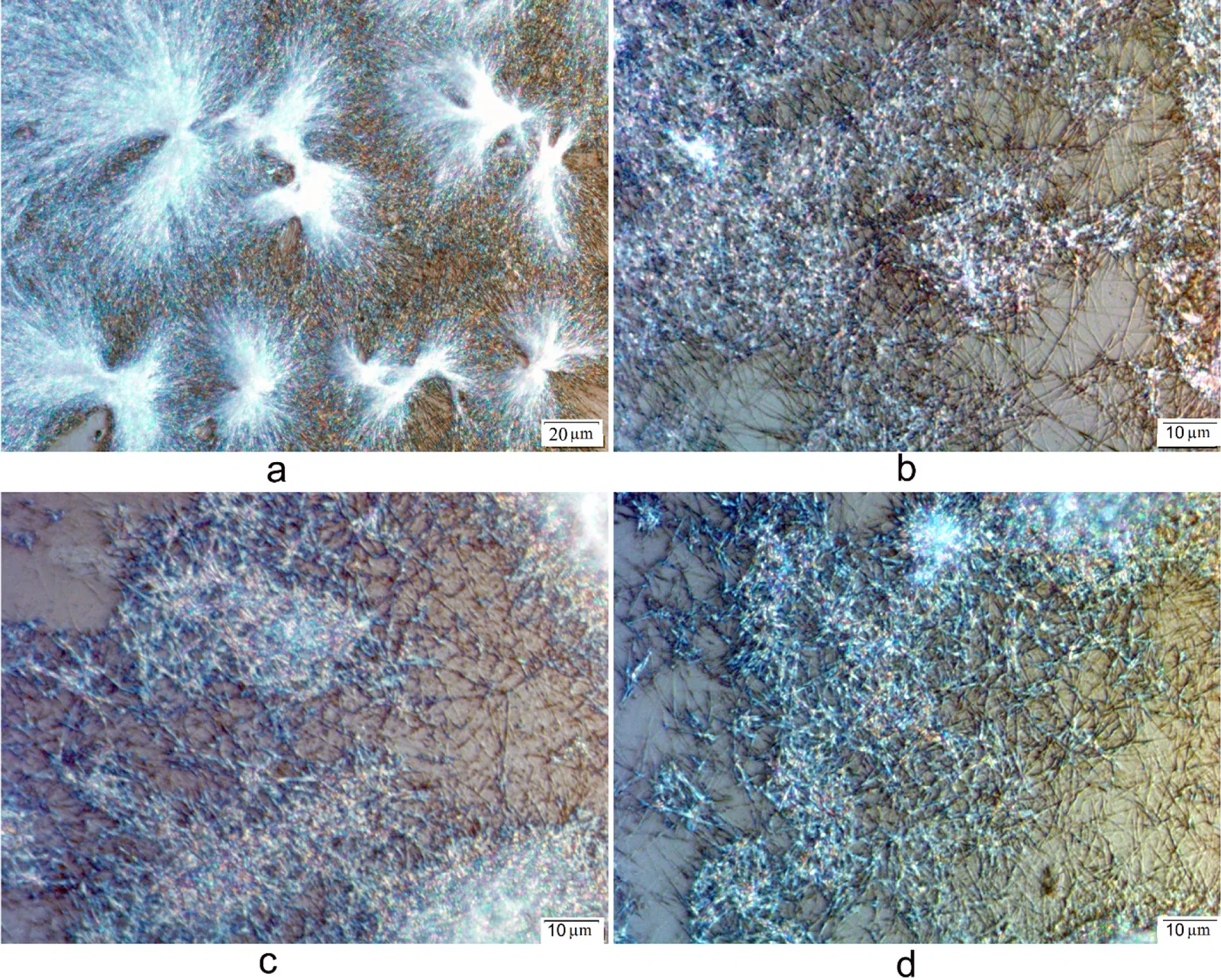
Compound **7** in DMSO:H_2_O (v/v 1:4) at 4.0 mg/mL in the presence of 1.0 equiv metal salts: (a, b) with NiCl_2_, (c) with FeCl_2_, (d) with Zn­(OAc)_2_.

**Figure 4:**
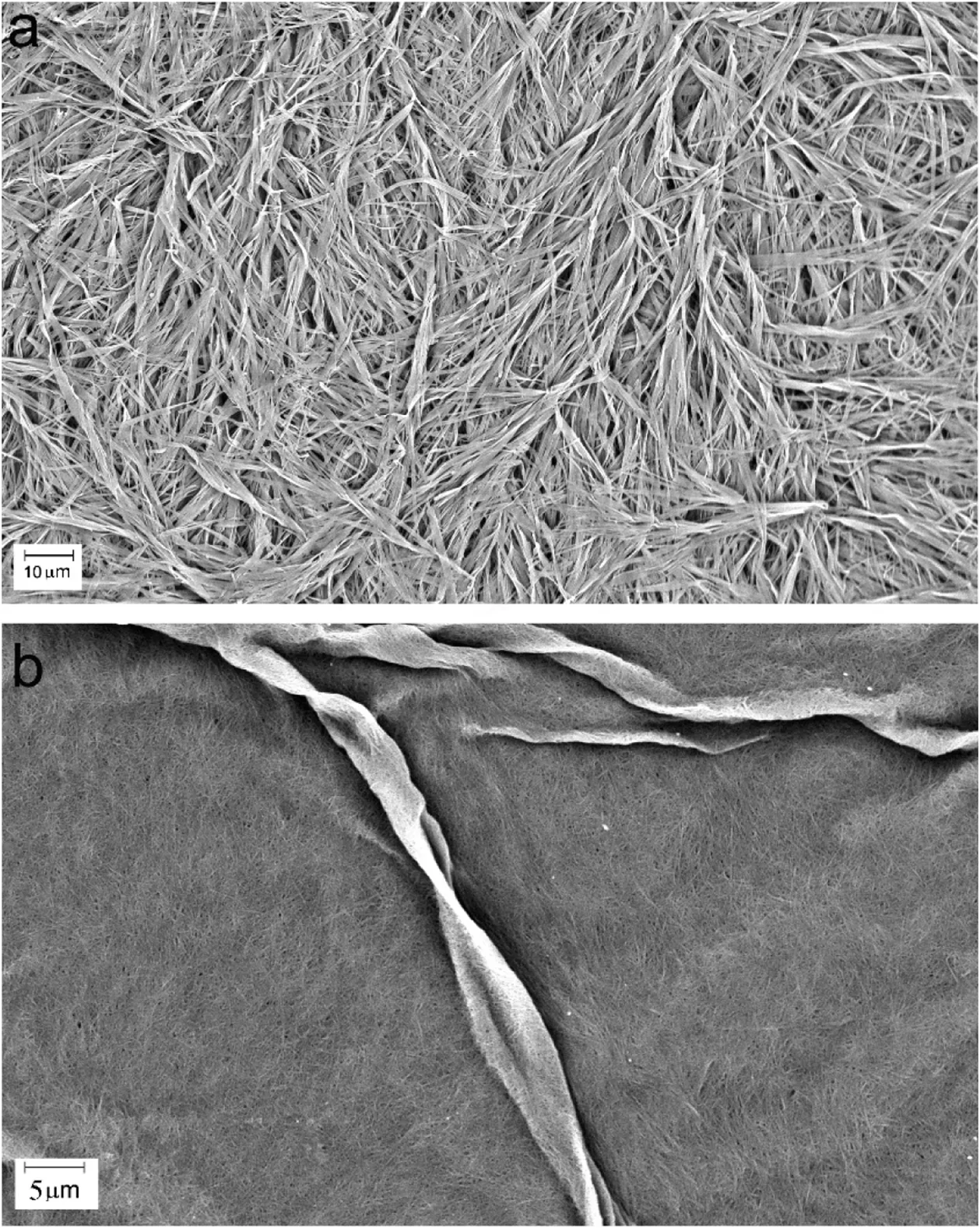
SEM images of gels formed by compounds **5** and **7**. (a) compound **5** in EtOH:H_2_O (v/v 1:2) at 6.7 mg/mL; (b) compound **7** in DMSO:H_2_O (v/v 1:2) at 1.8 mg/mL.

The cyclohexyl derivative compound **7** showed promise as an effective hydrogelator; in order to obtain the minimum amount of DMSO required for the compound to form a hydrogel, we further analyzed the gelation properties of the compound in different ratios of DMSO and water. Compound **7** was able to form a stable gel at DMSO:H_2_O ratio ranging from 1:1 to 1:10 at all tested ratios, indicating the versatility of the gelator. The gelation test results are included in Table S4, and the selected corresponding gel photos are shown in Figure S21. The DMSO:H_2_O (v/v 1:4) solvent system was selected for further gelation testing of the compound in the presence of several metal salts. The selected gel photos are shown in Figures S22–S24. The morphologies of a few of these metallogels are shown in Figure 3. The gel formed by compound **7** in DMSO:H_2_O (v/v 1:4) in the presence of nickel chloride showed more distinctive fiber morphologies (Figure 3a–b). In the same solvent system, the metallogels in the presence of Fe^2+^ and Zn^2+^ appeared very similar in morphology, both exhibiting very fine fiber assemblies consisting of narrow, and crosslinked short fibers (Figure 3c–d).

The morphologies of the dried gels were also characterized by using SEM. Both samples primarily exhibited a dense network of fibers, as shown in Figure 4. The gel formed by compound **5** in EtOH:H_2_O (v/v 1:2) at 6.7 mg/mL exhibited ribbons and loosely packed fibers (Figure 4a). The ribbons are at a length scale of an average hundreds of micrometers, and the branched ribbons were stacked but not highly intertwined. On the other hand, the gels formed by compound **7** exhibited a much smaller and uniform fibrous network (Figure 4b). The gel of **7** in DMSO:H_2_O (v/v 1:2) at 1.8 mg/mL exhibited a very long and uniform intertwined fibrous network, which is a general feature of the gel, and the fibers have small diameters.

These gels were further characterized by measuring their rheological properties. The gels formed by compound **7** showed viscoelastic properties, as shown in Figure 5. The three aqueous gels all exhibited higher G′ than G″ values for the screened frequencies, indicating predominantly elastic, gel-like behavior. The average G'/G″ ratios, calculated over the linear frequency range 0.1 to 10 rad/s, were 8.0, 11.9, and 14.9 for the three gels formed by compound **7** in EtOH:H_2_O (v/v 1:1), EtOH:H_2_O (v/v 1:2), and DMSO:H_2_O (v/v 1:2), respectively.

**Figure 5:**
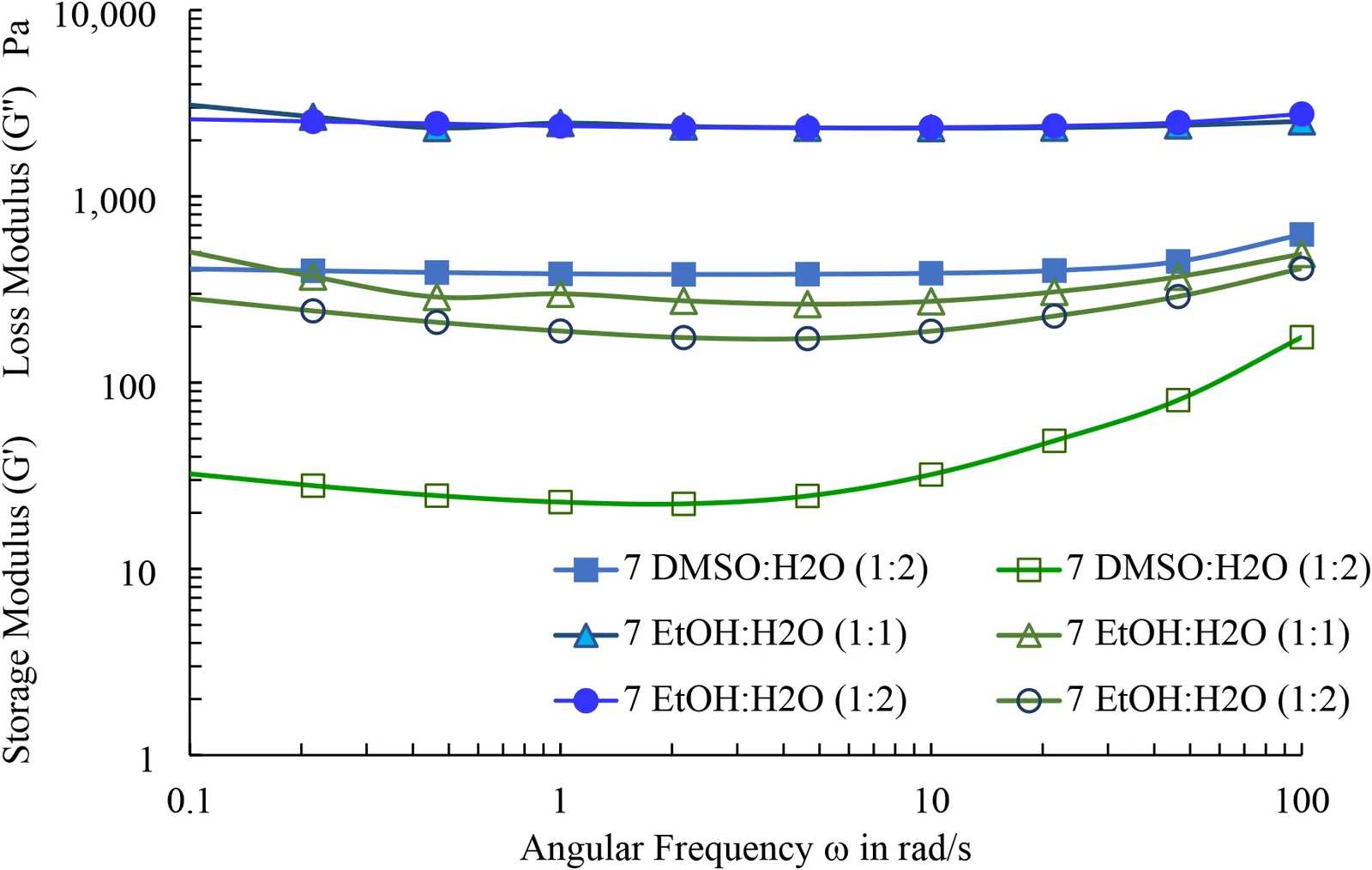
Rheological data for the gels formed by compound **7** in DMSO:H_2_O (v/v 1:2) at 1.8 mg/mL, EtOH:H_2_O (v/v 1:1) at 1.6 mg/mL, and EtOH:H_2_O (v/v 1:2) at 1.8 mg/mL. The strain was 0.5% for all samples. Filled symbols denote G′ (storage modulus) and open symbols denote G″ (loss modulus).

### Metallogel Formation and Chemiluminescence

The stability of the gels formed by compound **7** in the presence of metal salts was characterized by using a rheometer. The results are shown in Figure S25–S29, Tables S6–S9. For all these metal-organic gels, their G’ values were greater than G″ which confirms their viscoelastic behavior. The G'/G" ratios of the gels formed with Zn­(II), Ni­(II), Fe­(II), and Cu­(II) are 25.7, 24.4, 18.8, and 14.9, respectively. The G" values for Zn­(II) and Ni­(II) gels are greater than those of Fe­(II) and Cu­(II) gels.

The formation of metallogels has great potential for applications in various fields. Here, we utilized the metallogels formed in this study to explore their catalytic potential for the chemiluminescence of luminol using a similar procedure reported previously.^36^ The chemiluminescence experiment was carried out for the gels formed by compound **7** and the corresponding metallogels with Zn­(OAc)_2_, Cu­(OAc)_2_, NiCl_2_, and FeCl_2_. These results are shown in ESI Figure S32–36 and Tables S10 and S11. Using luminol and hydrogen peroxide, both the wet gels and dried xerogels formed with Cu­(OAc)_2_ exhibited strong chemiluminescence (CL) signals. In contrast, other metal ions such as Ni^2+^, Zn^2+^, and Fe^2+^ did not produce CL under the same conditions. The dried xerogel containing Cu­(OAc)_2_ showed much-enhanced CL, suggesting that the gel containing Cu^2+^ catalyzed the luminol and H_2_O_2_ reaction. These properties can be utilized for potential applications of detecting the presence of copper ions. In addition, the excellent CL generated by the Cu­(II) gelator complex for luminol/H_2_O_2_ can be utilized to detect dopamine and other biological markers. This hydrogelator/Cu­(OAc)_2_ system exhibited oxidase-mimicking properties for CL enhancement, and the system may be useful for dopamine detection as shown in the literature.^45^ In comparison to the CL in the C-2 carbamate derivatives, in which the xerogels with Cu^2+^, Zn^2+^, and Ni^2+^ all exhibited CL,^36^ for the C-3 carbamate derivative (compound **7**), only the Cu­(II) gels exhibited luminescence, though both wet gels and xerogels were effective.

### Dye Removal for Environmental Remediation

Compound **7** formed stable gels in DMSO–water mixtures at tested volume ratios from 1:1 to 1:10 . To evaluate its potential for dye removal, a gel column was prepared in a glass syringe for the dye removal study. As shown in Figure 6, 3.0 mg of compound **7** was heated and dissolved in 1 mL DMSO: H_2_O (1:2) solution. Then the hot solution was transferred into a gel column and allowed to settle at room temperature for 2 h. 1 mL of DI water was added on top of the column to flush DMSO from the gel column. After that, a toluidine blue dye solution (0.1 mL, 50 mg/L) was loaded onto the column and passed through the gel column. After the elution stopped, 1 mL of water was added to the top of the column and allowed to pass through the column. The total elute was combined (1.4 mL) and diluted to 2.0 mL for UV analysis. At the end of the experiment, the gel remained stable and could be inverted. Absorbance at λ_max_ (630 nm) of toluidine blue was measured using a calibration curve.^60^ Approximately 90% of the dye was removed from the initial solution (Figure 7).

**Figure 6:**
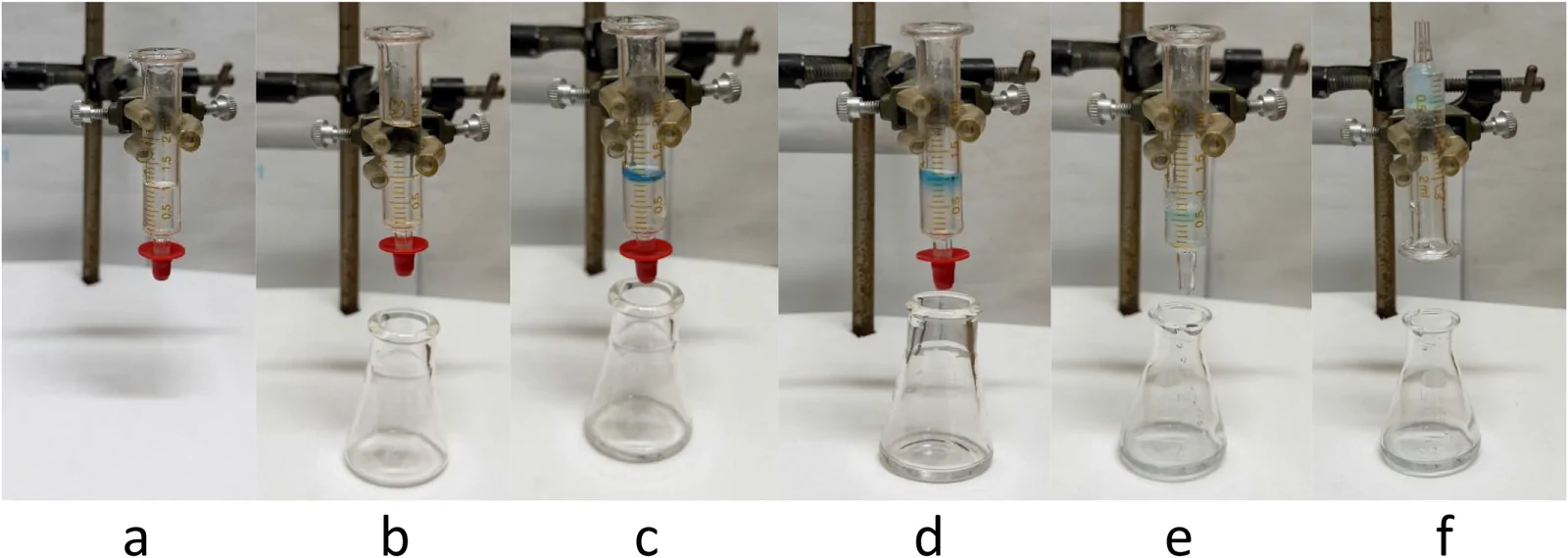
Starting from left: (a) gel column; (b) gel column with 1 mL DI water; (c) gel column after loading with 0.1 mL of 50 mg/L toluidine blue dye; (d) gel column after passing 0.1 mL of toluidine blue dye; (e) gel column with 1 mL water; and (f) inverted gel column with collected aqueous solution.

**Figure 7:**
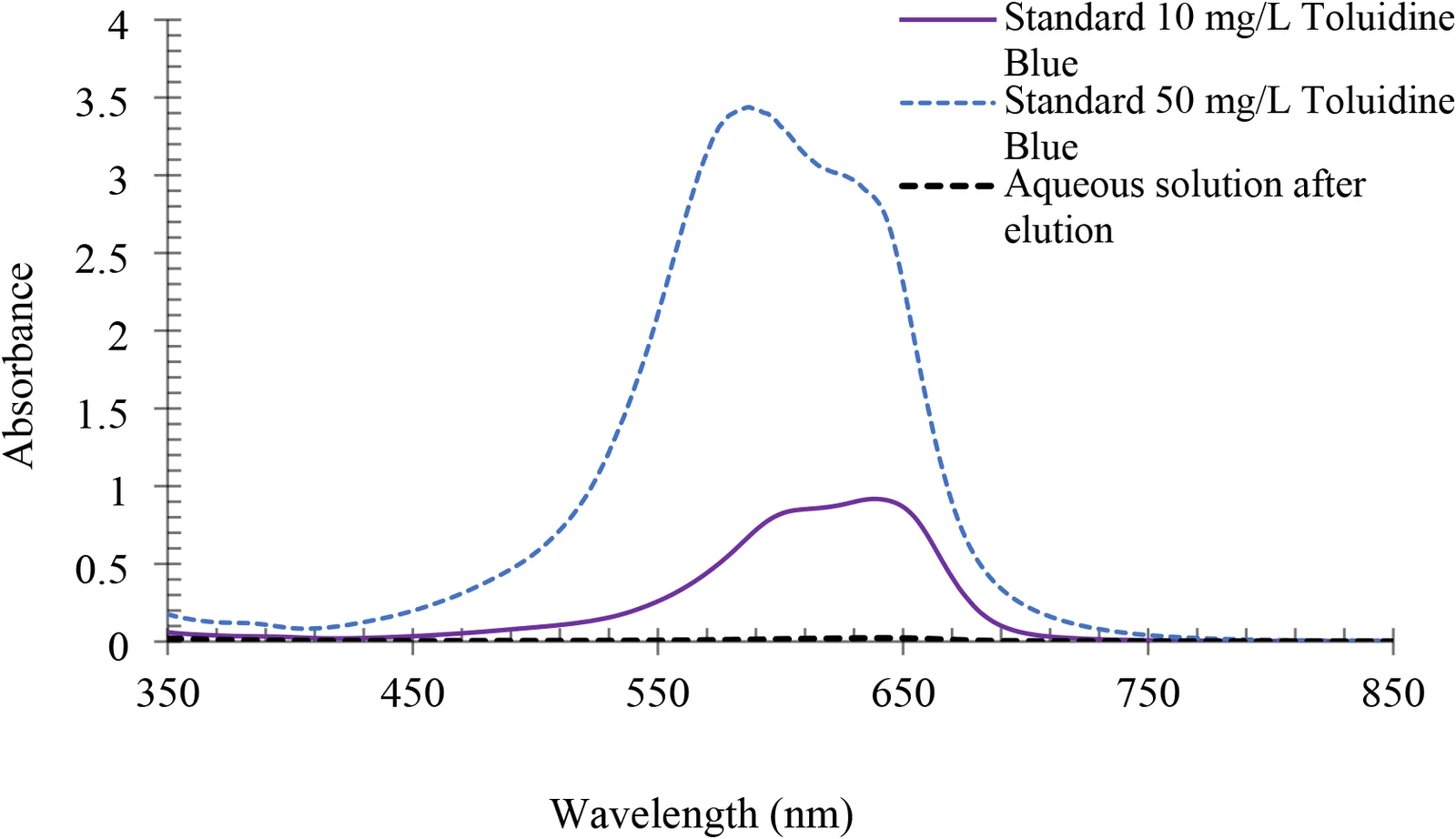
Overlaid UV-vis spectra of the initial toluidine blue solution and the recovered aqueous eluate from the dye removal experiment using a gel formed from 3.0 mg of compound **7** in 1 mL DMSO:H_2_O (1:2).

## Conclusions

A series of 12 3-O-carbamate derivatives of 4,6-O-phenyl ethylidene acetal-protected d-glucosamine was synthesized and characterized. Most of the derivatives were found to be effective organogelators, particularly in vacuum pump oil and glycerol. Several derivatives formed gels in mixtures of ethanol and water or dimethyl sulfoxide and water. Cyclohexyl carbamate **7** was found to be the most effective gelator, forming gels at relatively low gelator concentrations and in the presence of various metal ions. The morphological properties of selected gels were characterized by an optical microscope and scanning electron microscopy. The viscoelastic behavior of the gel and metallogel assemblies was analyzed using rheology. The catalytic potential of these metallogels was evaluated by using the luminol/H_2_O_2_ chemiluminescence system. Cu^2+^ metal–organic gels and xerogels both exhibited enhanced CL for luminol/H_2_O_2_, while the other metal ions did not show these peroxidase-like catalytic properties for luminol. In addition, the hydrogels formed by compound **7** were effective in removing toluidine dye from an aqueous solution.

## Experimental Section

Reagents and solvents were used as they were received from suppliers. All purification was conducted by flash chromatography using 230–400 mesh silica gel. The solvent systems used for chromatography and for gelation tests were all in volume ratios. NMR analysis was conducted using a 400 MHz Bruker NMR spectrometer. The molecular mass was measured using LCMS on the LC1260 system or Shimadzu LCMS-2020 with ESI in positive-ionization mode. Melting point measurements were carried out using a Stuart Automatic Melting point Apparatus SMP40. Fluorescence emission spectra were obtained using the Shimadzu RF-6000 Spectro Fluorophotometer with excitation and emission bandwidths set at 5.0 nm and a scan speed of 600 nm/min. The gelation tests and optical microscopy were carried out following literature report. The instruments used for optical microscopy were the Olympus BX60M optical microscope and Olympus DP73-1–51 high-performance 17MP digital camera with pixel shifting and Peltier cooled. SEM images were obtained from a Thermo Scientific Phenom XL G2 Desktop SEM. The gel sample was placed on the SEM stub, and air-dried for 5–7 days, and stored in a nitrogen-flushed desiccator for about 10 days. The dried samples were coated with silver for about 20 s before analysis. Chemiluminescence data were acquired using the Varioskan LUX multimode microplate reader by Thermo Scientific with a 96-well adapter for plates without lids. The data were obtained in a SkanIt software.

### Metallogel Preparation

In a one-dram vial, compound **7** (2.0 mg, 4.37 × 10^–3^ mmol) and 0.5 mL of metal stock solution were added, followed by 0.5 mL of DMSO:H_2_O (v/v 1:3) for preparing metallogels with 0.5 eq. metal salt with respect to 1.0 eq. gelator at 2.0 mg/mL. The solution was heated to dissolve the mixture and allowed to cool to room temperature for 15–20 min. If gelation occurred, an additional 0.5 eq metal ion solution was added to continue the gelation test.

### Dye Removal Study

The gel column was prepared by using 3 mg of compound **7** in 1 mL of DMSO:H_2_O (1:2) solution. Then, the mixture was heated, and transferred into a column, where it was allowed to settle for 2 h to form a stable gel. To remove DMSO, 1 mL of DI water was added to the top of the column. Then, after passing water, toluidine blue dye 0.1 mL (50 mg/L) was added on top of the gel column and allowed to diffuse for half an hour. The dye then passed through the gel, followed by the addition of 1 mL of water. The eluted liquid (1.4 mL) was collected and diluted to 2 mL for UV measurements. At the end of the experiment, the gel was stable enough to be inverted.

### Chemiluminescence (CL) Measurement

In a one-dram vial, compound **7** (2.0 mg) was added to the selected metal salt (1.0 equiv) in 0.5 mL of DMSO:H_2_O (v/v 1:4) mixture; a gel was prepared in the vial (4.0 mg/mL) and was left to stand at rt for at least overnight. Two sets of each metallogel and a control gel (without metal salt) were prepared. One set of metallogels and the control gel were air-dried for 48 h to form the corresponding xerogels. For chemiluminescence studies, the xerogels were diluted with 0.5 mL Millipore grade water and sonicated to form a suspension. The resulting suspension (100 μL) and a luminol solution (100 μL of a 2.0 mg/mL solution prepared in 10.0 mM NaOH) were added in a 96-well plate. A solution of H_2_O_2_ (0.3% or 3% solution in water) was added, and the luminescence intensity was recorded immediately.

**Synthesis of compound 2:** The compound was prepared by a similar method in the literature.^53^ Compound **1** (1.5 g, 4.44 mmol, 1.0 equiv.) was reacted with carbonyldiimidazole (1.08 g, 6.66 mmol, 1.5 equiv.) in anhy. DCM for 2.5 h. The product was obtained by organic phase separation from the aqueous phase to yield a white solid (1.8 g, 4.17 mmol, 95%). M.P. = 217.0–219.0 °C; ^1^H NMR (400 MHz, CDCl_3_) *δ* 8.08 (s, 1H), 7.35 (m, 1H), 7.15–7.11 (m, 5H), 7.06 (m, 1H), 5.88 (d, *J* = 9.7 Hz, 1H), 5.30 (t, *J* = 10.1 Hz, 1H), 4.71 (d, *J* = 3.6 Hz, 1H), 4.67 (t, *J* = 5.2 Hz, 1H), 4.53–4.46 (m, 1H), 4.20–4.15 (m, 1H), 3.84–3.77 (m, 1H), 3.59–3.52 (m, 2H), 3.41 (s, 3H), 2.96–2.84 (m, 2H), 1.92 (s, 3H). ^13^C NMR (100 MHz, CDCl_3_) *δ* 170.0, 148.8, 137.3, 135.7, 130.7, 129.6, 128.1, 126.7, 117.3, 102.8, 99.1, 78.6, 75.0, 68.4, 62.8, 55.4, 51.5, 40.6, 23.2.

Compounds **3–14** were synthesized following the literature procedure.^53^ The detailed synthesis of compound **3** is given here. For all others, only the numbers of reactants and the product characterizations are listed below.

**Synthesis of compound 3:** In a 50 mL round-bottom flask, compound **2** (75 mg, 0.17 mmol, 1.0 equiv) and DCE (3.0 mL) were added. To this mixture, propyl amine (30 μL, 0.35 mmol, 2.0 equiv.) was added, and the reaction was allowed to stir at 30 °C for 4 h. The reaction was monitored using TLC and ^1^H NMR spectroscopy. After completion of the reaction, the solvent was removed using a rotavap to afford a light yellow solid crude product. The crude was purified by column chromatography to afford a light yellow solid (61 mg, 0.14 mmol, 85%) as the desired product. (R_f_ value in 5% MeOH/DCM = 0.35) M.P. = 187.0–189.0 °C; ^1^H NMR (400 MHz, CDCl_3_) *δ* 7.29–7.18 (m, 5H), 6.05–5.87 (m, 1H), 5.15–5.03 (m, 1H), 4.80–4.65 (m, 3H), 4.24–4.08 (m, 2H), 3.79–3.69 (m, 1H), 3.49 (t, *J* = 10.4 Hz, 1H), 3.43–3.34 (m, 4H), 3.21–3.08 (m, 2H), 2.98 (dd, *J* = 14.0, 4.8 Hz, 1H), 2.90 (dd, *J* = 14.0, 5.6 Hz, 1H), 1.96 (s, 3H), 1.54–1.48 (m, 2H), 0.93 (t, *J* = 7.3 Hz, 3H); ^13^C NMR (100 MHz, CDCl_3_) *δ* 170.2, 156.5, 136.2, 129.8, 128.1, 126.5, 102.7, 99.0, 78.9, 70.7, 68.5, 62.9, 55.3, 53.0, 42.8, 40.9, 23.2, 23.1, 11.1. LC-MS (ESI+) calcd for C_21_H_31_N_2_O_7_ [M + H]^+^ 423.2 found 423.

**Synthesis of compound 4:** Compound **2** (75 mg, 0.17 mmol, 1.0 equiv) and butyl amine (34 μL, 0.35 mmol, 2.0 equiv) were used as reactants. The crude product was purified by column chromatography to afford a light yellow solid (63 mg, 0.14 mmol, 85%) as the desired product. (R_f_ value in 5% MeOH/DCM = 0.40) M.P. = 191.0 – 194.0 °C; ^1^H NMR (400 MHz, CDCl_3_) *δ* 7.29–7.19 (m, 5H), 6.05–5.93 (m, 1H), 5.14–5.04 (m, 1H), 4.76–4.63 (m, 3H), 4.24–4.08 (m, 2H), 3.79–3.70 (m, 1H), 3.49 (t, *J* = 10.4 Hz, 1H), 3.44–3.34 (m, 4H), 3.25–3.11 (m, 2H), 2.98 (dd, *J* = 14.0, 4.8 Hz, 1H), 2.90 (dd, *J* = 14.0, 5.6 Hz, 1H), 1.96 (s, 3H), 1.54–1.42 (m, 2H), 1.39–1.31 (m, 2H), 0.93 (t, *J* = 7.3 Hz, 3H);^13^C NMR (100 MHz, CDCl_3_) *δ* 170.1, 156.5, 136.2, 129.8, 128.1, 126.5, 102.7, 99.0, 78.9, 70.7, 68.5, 62.9, 55.3, 53.0, 40.86, 40.82, 32.0, 23.2, 19.8, 13.7. LC-MS (ESI+) calcd for C_22_H_33_N_2_O_7_ [M + H]^+^ 437.2 found 437.

**Synthesis of compound 5:** Compound **2** (75 mg, 0.17 mmol, 1.0 equiv.) and isobutyl amine (35 μL, 0.35 mmol, 2.0 equiv.) were used as reactants. The crude product was purified by column chromatography to afford a white solid (57 mg, 0.13 mmol, 77%) as the desired product. (R_f_ value in 5% MeOH/DCM = 0.30) M.P. = 187.0 – 190.0 °C; ^1^H NMR (400 MHz, CDCl_3_) *δ* 7.31–7.21 (m, 5H), 6.05–5.93 (m, 1H), 5.15–5.05 (m, 1H), 4.84–4.76 (m, 1H), 4.74–4.67 (m, 2H), 4.25–4.09 (m, 2H), 3.80–3.71 (m, 1H), 3.51 (t, *J* = 10.4 Hz, 1H), 3.46–3.36 (m, 4H), 3.13–2.86 (m, 4H), 1.96 (s, 3H), 1.82–1.71 (m, 1H), 0.96–0.89 (m, 6H); ^13^C NMR (100 MHz, CDCl_3_) *δ* 169.1, 155.7, 135.2, 128.8, 127.1, 125.5, 101.7, 98.0, 77.9, 69.6, 67.5, 61.9, 54.3, 52.0, 47.5, 39.9, 27.8, 22.2, 18.8. LC-MS (ESI+) calcd for C_22_H_33_N_2_O_7_ [M + H]^+^ 437.2 found 437.

**Synthesis of compound 6:** Compound **2** (100 mg, 0.23 mmol, 1.0 equiv.) and hexyl amine (36 μL, 0.27 mmol, 1.2 equiv.) were used as the reactants. The crude product was purified by column chromatography to afford a white solid (93.4 mg, 0.20 mmol, 88%) as the desired product. (R_f_ value in 5% MeOH/DCM = 0.62) M.P. = 171.0 – 173.0 °C; ^1^H NMR (400 MHz, CDCl_3_) *δ* 7.29–7.19 (m, 5H), 6.02–5.92 (m, 1H), 5.13–5.04 (m, 1H), 4.77–4.63 (m, 3H), 4.24–4.07 (m, 2H), 3.79–3.69 (m, 1H), 3.49 (t, *J* = 10.4 Hz, 1H), 3.43–3.32 (m, 4H), 3.25–3.09 (m, 2H), 2.98 (dd, *J* = 14.0, 4.8 Hz, 1H), 2.89 (dd, *J* = 14.0, 5.6 Hz, 1H), 1.96 (s, 3H), 1.56–1.43 (m, 2H), 1.36–1.23 (m, 6H), 0.92–0.86 (m, 3H); ^13^C NMR (100 MHz, CDCl_3_) *δ* 170.2, 156.5, 136.2, 129.8, 128.1, 126.5, 102.7, 99.0, 78.9, 70.7, 68.5, 62.9, 55.3, 53.0, 41.2, 40.9, 31.5, 29.8, 26.4, 23.2, 22.5, 14.0. LC-MS (ESI+) calcd for C_24_H_37_N_2_O_7_ [M + H]^+^ 465.2 found 465.

**Synthesis of compound 7:** Compound **2** (70 mg, 0.16 mmol, 1.0 equiv.) and cyclohexyl amine (22 μL, 0.19 mmol, 1.2 equiv.) were used as the reactants. The crude product was purified by column chromatography to afford a white solid (44 mg, 0.09 mmol, 60%) as the desired product. (R_f_ value in 5% MeOH/DCM = 0.37) M.P. = 211.0–212.0 °C; ^1^H NMR (400 MHz, CDCl_3_) *δ* 7.29–7.18 (m, 5H), 6.05–5.96 (m, 1H), 5.15–5.00 (m, 1H), 4.75–4.60 (m, 3H), 4.24–4.07 (m, 2H), 3.79–3.70 (m, 1H), 3.57–3.32 (m, 6H), 2.98 (dd, *J* = 14.0, 4.8 Hz, 1H), 2.89 (dd, *J* = 14.0, 5.6 Hz, 1H), 1.95 (s, 3H), 1.95–1.85 (m, 2H), 1.70–1.50 (m, 3H), 1.40–1.10 (m, 5H); ^13^C NMR (100 MHz, CDCl_3_) *δ* 170.1, 155.6, 136.2, 129.8, 128.2, 126.5, 102.7, 99.0, 78.9, 70.5, 68.5, 62.9, 55.3, 53.1, 50.0, 40.9, 33.2, 33.1, 25.4, 24.7, 24.6, 23.2. LC-MS (ESI+) calcd for C_24_H_35_N_2_O_7_ [M + H]^+^ 463.2 found 463.

**Synthesis of compound 8:** Compound **2** (100 mg, 0.23 mmol, 1.0 equiv.) and ethanol amine (14 μL, 0.27 mmol, 1.2 equiv.) were used as reactants. The crude product was purified by column chromatography to afford a white solid (86.6 mg, 0.20 mmol, 89%) as the desired product. (R_f_ value in 6% MeOH/DCM = 0.62) M.P. = 194.0 – 197.0 °C; ^1^H NMR (400 MHz, CDCl_3_) *δ* 7.30–7.19 (m, 5H), 6.02–5.92 (m, 1H), 5.23–5.00 (m, 2H), 4.72–4.63 (m, 2H), 4.26–4.09 (m, 2H), 3.79–3.61 (m, 3H), 3.54–3.35 (m, 6H), 3.30–3.18 (m, 2H), 2.98 (dd, *J* = 14.0, 4.8 Hz, 1H), 2.89 (dd, *J* = 14.0, 5.6 Hz, 1H), 1.97 (s, 3H); ^13^C NMR (100 MHz, CDCl_3_) *δ* 171.0, 157.0, 136.1, 129.7, 128.2, 126.6, 121.6, 102.8, 99.0, 78.7, 71.4, 68.5, 62.8, 61.6, 55.3, 52.8, 43.7, 40.8, 23.2. LC-MS (ESI+) calcd for C_20_H_29_N_2_O_8_ [M + H]^+^ 425.2 found 425.

**Synthesis of compound 9:** Compound **2** (50 mg, 0.12 mmol, 1.0 equiv) and benzyl amine (15 μL, 0.14 mmol, 1.2 equiv) were used as reactants. The crude product was purified by column chromatography to afford a light-yellow solid (45.4 mg, 0.09 mmol, 80%) as the desired product. (R_f_ value in 5% MeOH/DCM = 0.60) M.P. = 197.0 – 200.0 °C; ^1^H NMR (400 MHz, CDCl_3_) *δ* 7.37–7.17 (m, 10H), 6.02–5.88 (m, 1H), 5.18–5.07 (m, 2H), 4.73–4.63 (m, 2H), 4.46–4.30 (m, 2H), 4.26–4.17 (m, 1H), 4.16–4.09 (m, 1H), 3.80–3.70 (m, 1H), 3.49 (t, *J* = 10.4 Hz, 1H), 3.44–3.34 (m, 4H), 2.98 (dd, *J* = 14.0, 4.8 Hz, 1H), 2.90 (dd, *J* = 14.0, 5.6 Hz, 1H), 1.89 (s, 3H); ^13^C NMR (100 MHz, CDCl_3_) *δ* 170.2, 156.6, 138.2, 136.1, 129.8, 128.7, 128.2, 127.6, 127.35, 126.5, 102.7, 99.0, 78.9, 71.1, 68.5, 62.9, 55.3, 52.9, 45.1, 40.9, 23.2. LC-MS (ESI+) calcd for C_25_H_31_N_2_O_7_ [M + H]^+^ 471.2 found 471.

**Synthesis of compound 10:** Compound **2** (75 mg, 0.17 mmol, 1.0 equiv.) and 4-methoxybenzyl amine (34 μL, 0.26 mmol, 1.5 equiv.) were used as the reactants. The crude product was purified by column chromatography to afford a light-yellow solid (78 mg, 0.15 mmol, 92%) as the desired product. (R_f_ value in 5% MeOH/DCM = 0.49) M.P. = 207.0 – 209.0 °C; ^1^H NMR (400 MHz, CDCl_3_) *δ* 7.26–7.16 (m, 7H), 6.90–6.82 (d, *J* = 8.5 Hz, 2H), 6.00–5.92 (m, 1H), 5.18–5.00 (m, 2H), 4.72–4.63 (m, 2H), 4.38–4.15 (m, 3H), 4.15–4.08 (m, 1H), 3.82–3.70 (m, 4H), 3.49 (t, *J* = 10.4 Hz, 1H), 3.45–3.34 (m, 4H), 2.98 (dd, *J* = 14.0, 4.8 Hz, 1H), 2.90 (dd, *J* = 14.0, 5.6 Hz, 1H), 1.90 (s, 3H); ^13^C NMR (100 MHz, CDCl_3_) *δ* 170.2, 159.1, 156.5, 136.2, 130.3, 129.8, 128.8, 128.2, 126.5, 114.1, 102.7, 99.0, 78.9, 71.0, 68.5, 62.9, 55.3, 55.26, 52.9, 45.0, 40.9, 23.2. LC-MS (ESI+) calcd for C_26_H_33_N_2_O_8_ [M + H]^+^ 501.2 found 501.

**Synthesis of compound 11:** Compound **2** (70 mg, 0.16 mmol, 1.0 equiv.) and 4-fluoro benzylamine (22 μL, 0.19 mmol, 1.2 equiv.) were used as the reactants. The crude product was purified by column chromatography to afford a white solid (54 mg, 0.11 mmol, 69%) as the desired product. (R_f_ value in 5% MeOH/DCM = 0.40) M.P. = 215.0 – 217.0 °C; ^1^H NMR (400 MHz, CDCl_3_) *δ* 7.26–7.17 (m, 7H), 7.06–6.94 (m, 2H), 5.98–5.88 (m, 1H), 5.17–5.06 (m, 2H), 4.72–4.63 (m, 2H), 4.41–4.17 (m, 3H), 4.16–4.09 (m, 1H), 3.79–3.70 (m, 1H), 3.49 (t, *J* = 10.4 Hz, 1H), 3.44–3.32 (m, 4H), 2.98 (dd, *J* = 14.0, 4.8 Hz, 1H), 2.90 (dd, *J* = 14.0, 5.6 Hz, 1H), 1.88 (s, 3H); ^13^C NMR (100 MHz, CDCl_3_) *δ* 169.1, 161.2 (d, ^1^ *J*_CF_ = 245.8 Hz, 162.4, 160.0), 155.5, 135.1, 133.1 (d, ^4^ *J*_CF_ = 3.3 Hz, 133.1, 133.0), 128.7, 128.1 (d, ^3^ *J*_CF_ = 8.1 Hz, 128.1, 128.0), 127.2, 125.5, 114.5 (d, ^2^ *J*_CF_ = 21.5 Hz, 114.6, 114.4), 101.7, 98.0, 77.8, 70.2, 67.5, 61.9, 54.3, 51.8, 43.4, 39.8, 22.2. LC-MS (ESI+) calcd for C_25_H_30_FN_2_O_7_ [M + H]^+^ 489.2 found 489.

**Synthesis of compound 12:** Compound **2** (80 mg, 0.19 mmol, 1.0 equiv.) and 2-picolyl amine (24 μL, 0.24 mmol, 1.3 equiv.) were used as the reactants. The crude product was purified by column chromatography to afford a white solid (66.0 mg, 0.14 mmol, 74%) as the desired product. (R_f_ value in 5% MeOH/DCM = 0.38) M.P. = 192.0 – 194.0 °C; ^1^H NMR (400 MHz, CDCl_3_) *δ* 8.58 (∼d, *J* = 4.7 Hz, 1H), 7.78–7.70 (m, 1H), 7.38–7.16 (m, 7H), 6.01 (d, *J* = 8.8 Hz, 1H), 5.91 (t, *J* = 5.4 Hz, 1H), 5.15 (∼t, *J* = 10.0 Hz,1H), 4.75–4.68 (m, 2H), 4.62–4.50 (m, 2H), 4.28–4.20 (m, 1H), 4.18–4.11 (m, 1H), 3.82–3.73 (m, 1H), 3.55–3.44 (m, 2H), 3.38 (s, 3H), 2.99 (dd, *J* = 14.0, 4.8 Hz, 1H), 2.90 (dd, *J* = 14.0, 5.6 Hz, 1H), 1.93 (s, 3H). ^13^C NMR (100 MHz, CDCl_3_) *δ* 170.2, 156.7, 156.5, 148.8, 137.2, 136.1, 129.7, 128.2, 126.5, 122.5, 121.7, 102.7, 99.0, 78.9, 71.1, 68.5, 62.9, 55.3, 52.9, 45.9, 40.8, 23.2. LC-MS (ESI+) calcd for C_24_H_30_N_3_O_7_ [M + H]^+^ 472.2 found 472.2.

**Synthesis of compound 13**: Compound **2** (100 mg, 0.23 mmol, 2.0 equiv.) and 1,6-hexanediamine (15 μL, 0.12 mmol, 1.0 equiv.) were used as reactants. The crude product was purified by column chromatography to afford a white solid (73 mg, 0.09 mmol, 72%) as the desired product. (R_f_ value in 5% MeOH/DCM = 0.63) M.P. = 243.0 – 244.0 °C; ^1^H NMR (400 MHz, CDCl_3_) *δ* 7.29–7.17 (m, 10H), 6.24–6.13 (m, 2H), 5.14–5.03 (m, 2H), 4.87–4.79 (m, 2H), 4.74–4.63 (m, 4H), 4.25–4.06 (m, 4H), 3.80–3.71 (m, 2H), 3.54–3.45 (m, 2H), 3.45–3.34 (m, 8H), 3.29–3.04 (m, 4H), 2.97 (dd, *J* = 14.0, 4.8 Hz, 2H), 2.89 (dd, *J* = 14.0, 5.8 Hz, 2H), 1.96 (s, 6H), 1.55–1.24 (m, 8H); ^13^C NMR (100 MHz, CDCl_3_) *δ* 170.3, 156.4, 136.2, 129.8, 128.1, 126.5, 102.7, 99.1, 79.1, 70.6, 68.5, 62.8, 55.3, 52.9, 40.9, 40.6, 29.8, 25.9, 23.2. LC-MS (ESI+) calcd for C_42_H_59_N_4_O_14_ [M + H]^+^ 843.4 found 843.5.

**Synthesis of compound 14:** Compound **2** (100 mg, 0.23 mmol, 2.0 equiv) and m-xylene amine (18 μL, 0.12 mmol, 1.0 equiv) were used as the reactants. The crude product was purified by column chromatography to afford a white solid (81 mg, 0.09 mmol, 67%) as the desired product. (R_f_ value in 5% MeOH/DCM = 0.55) M.P. = 276.0 – 278.0 °C; ^1^H NMR (400 MHz, CDCl_3_) *δ* 7.36 (m, 1H), 7.27–7.22 (m, 11H), 7.17–7.12 (m, 2H), 6.51–6.45 (m, 2H), 5.84–5.78 (m, 2H), 5.20–5.15 (m, 2H), 4.77–4.55 (m, 6H), 4.37–4.28 (m, 2H), 4.21–4.08 (m, 4H), 3.85–3.73 (m, 2H), 3.59–3.44 (m, 4H), 3.39 (m, 6H), 2.96–2.89 (m, 4H), 1.74 (s, 6H); ^13^C NMR (100 MHz, CDCl_3_) *δ* 170.8, 156.7, 139.5, 136.1, 129.9, 128.7, 128.1, 126.5, 125.9, 125.0, 102.6, 99.4, 79.1, 71.2, 68.6, 62.7, 55.6, 52.6, 44.5, 40.8, 22.8. LC-MS (ESI+) calcd for C_44_H_55_N_4_O_14_ [M + H]^+^ 863.4 found 863.5.

## Supplementary Material



## Notes

The ^1^H and ^13^C NMR spectra for compounds **2–14**; the 2D-COSY and HSQC NMR spectra for compounds **5** and **7**; rheological properties of selected gels of compound **7** and hydrogels in the presence of metal ions; further gelation tests of compound **7** in DMSO/water and metallogel formation; photographs of gels; FTIR spectra of compound **7**, and chemiluminescence experimental details are provided in the ESI (PDF)
