# Supplementary material for: Carbamate-Functionalized N‑Acetyl-D-Glucosamine Derivatives for Gel Formation and Chemiluminescent Properties of their Metallogels
Source: ACS Omega. 2026 Sep 16;11(38):57464–75. doi: 10.1021/acsomega.6c05849 (PMC13628620; doi:10.1021/acsomega.6c05849)
Supplement: Supplementary file 1 [file ao6c05849_si_001.pdf]

Supplementary Materials

Carbamate Functionalized N-Acetyl-D-Glucosamine Derivatives for Gel Formation and Chemiluminescent Properties of their Metallogels

Pooja Sharma, Pramod Aryal, Richard Chen, Alice Mao, Guijun Wang*

Department of Chemistry and Biochemistry, Old Dominion University, Norfolk, VA 23529, g1wang@odu.edu

Content Page

I. NMR spectra of compounds **2-14** S3

- ^{1}H and ^{13}C NMR spectra of **2-14** S3
- 2D NMR spectra of compounds **5** and **7** S16

II. Rheological properties of selected gels of compound **7** S20

III. Gelation test of compound **7** in DMSO/water and metallogels formation S25

IV. Rheological properties of metallogels formed by compound **7** S29

V. Dye absorption studies S36

VI. FTIR spectra of compound **7** S37

VII. Chemiluminescent experiments S39

General Methods

Reagents and solvents were used as received from suppliers such as Sigma-Aldrich, VWR, Fisher, and Ambeed. All purifications were performed by flash chromatography using 230–400 mesh silica gel. The solvent systems used for chromatography and gelation tests are all expressed as volume ratios. NMR analysis was conducted using a 400 MHz Bruker NMR spectrometer. The molecular mass was measured by LC-MS on the LC-1260 system or the Shimadzu LCMS-2020 using ESI in positive ionization mode. Melting point measurements were carried out using a Stuart automatic melting point apparatus, SMP40. Fluorescence emission spectra were obtained using a Shimadzu RF-6000 Spectrofluorophotometer with excitation and emission bandwidths set at 5.0 nm and a scan speed of 600 nm/min. The gelation tests and optical microscopy were carried out as described in the literature. The instrument used for optical microscopy is an Olympus BX60M optical microscope and the Olympus DP73-1-51 high-performance 17MP digital camera with pixel shifting and Peltier cooling. SEM images were obtained from Thermo Scientific Phenom XL G2 Desktop SEM. The gel sample was placed on the SEM button and air-dried for 5-7 days, and stored in a nitrogen-flushed desiccator for about 10 days. The dried samples were coated with silver for about 20 s before analysis. Chemiluminescence data were acquired using the Varioskan LUX multimode microplate reader (Thermo Scientific) with a 96-well adapter for plates without lids. The data was obtained in SkanIt software.

I. NMR spectra of the synthesized compounds

1. ^{1}H and ^{13}C NMR spectra of **2-14**

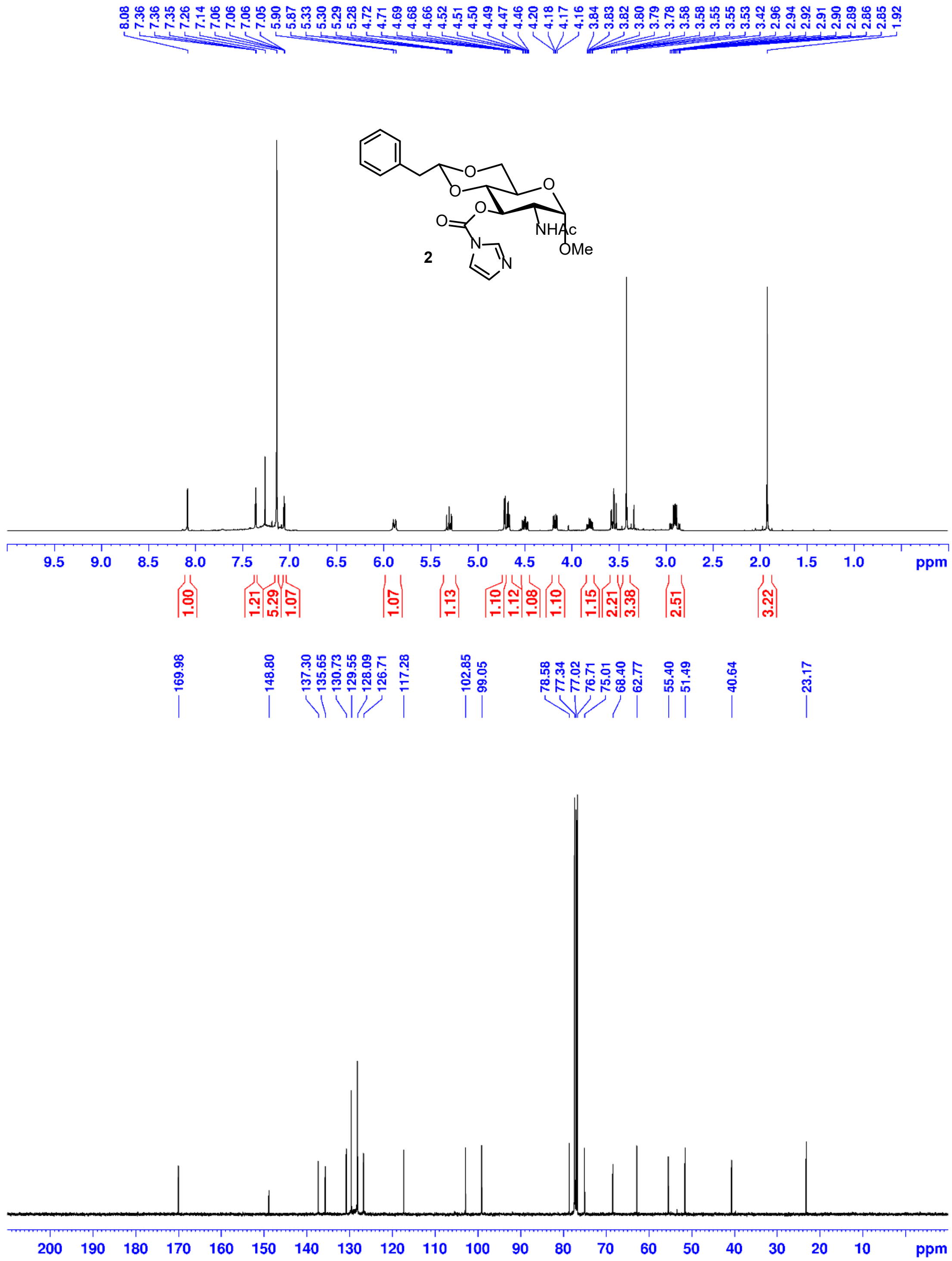

Figure S1. ^{1}H NMR and ^{13}C NMR spectra for compound **2** in $CDCl_3$

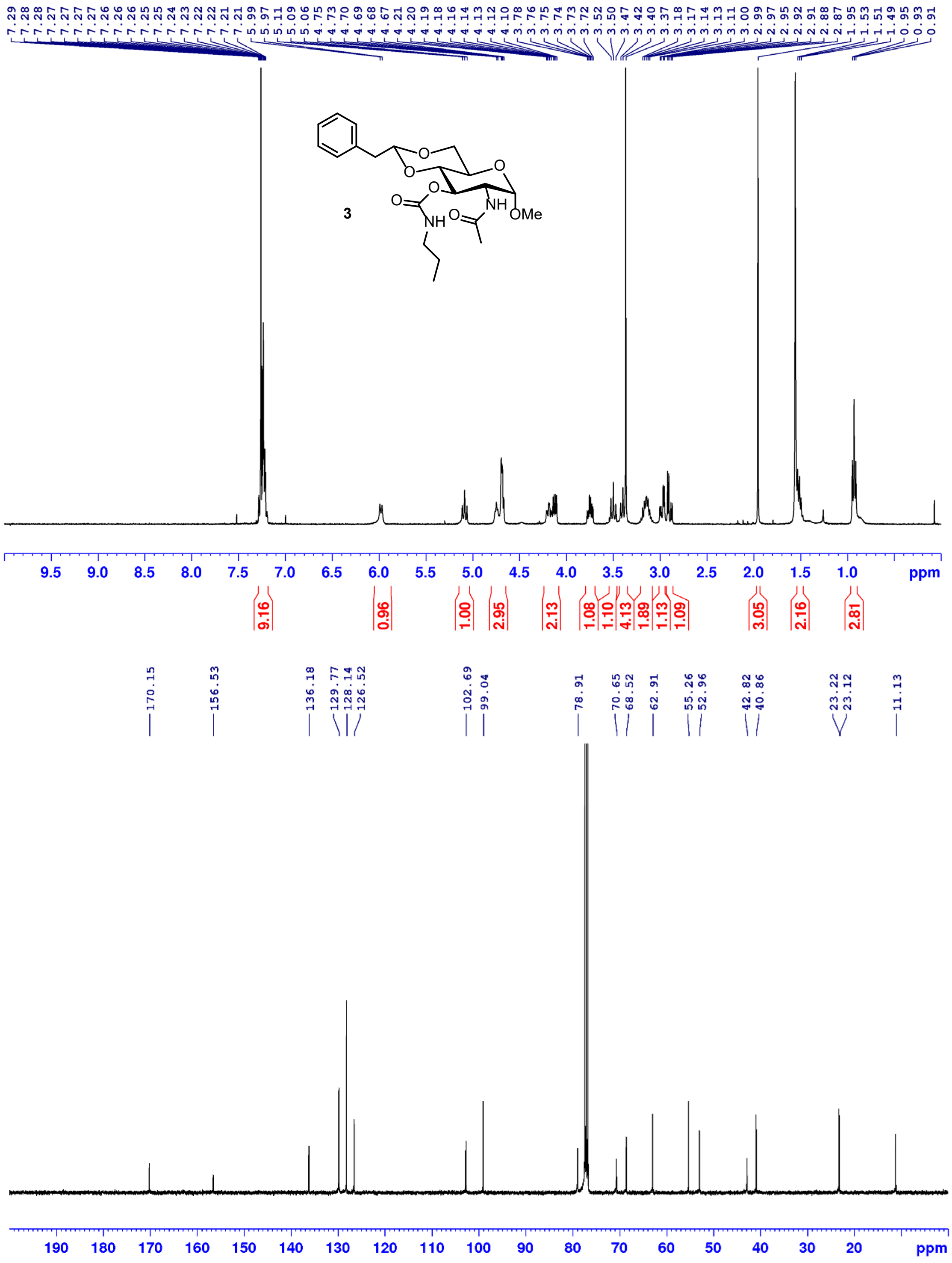

Figure S2. ^{1}H NMR and ^{13}C NMR spectra for compound **3** in $CDCl_3$

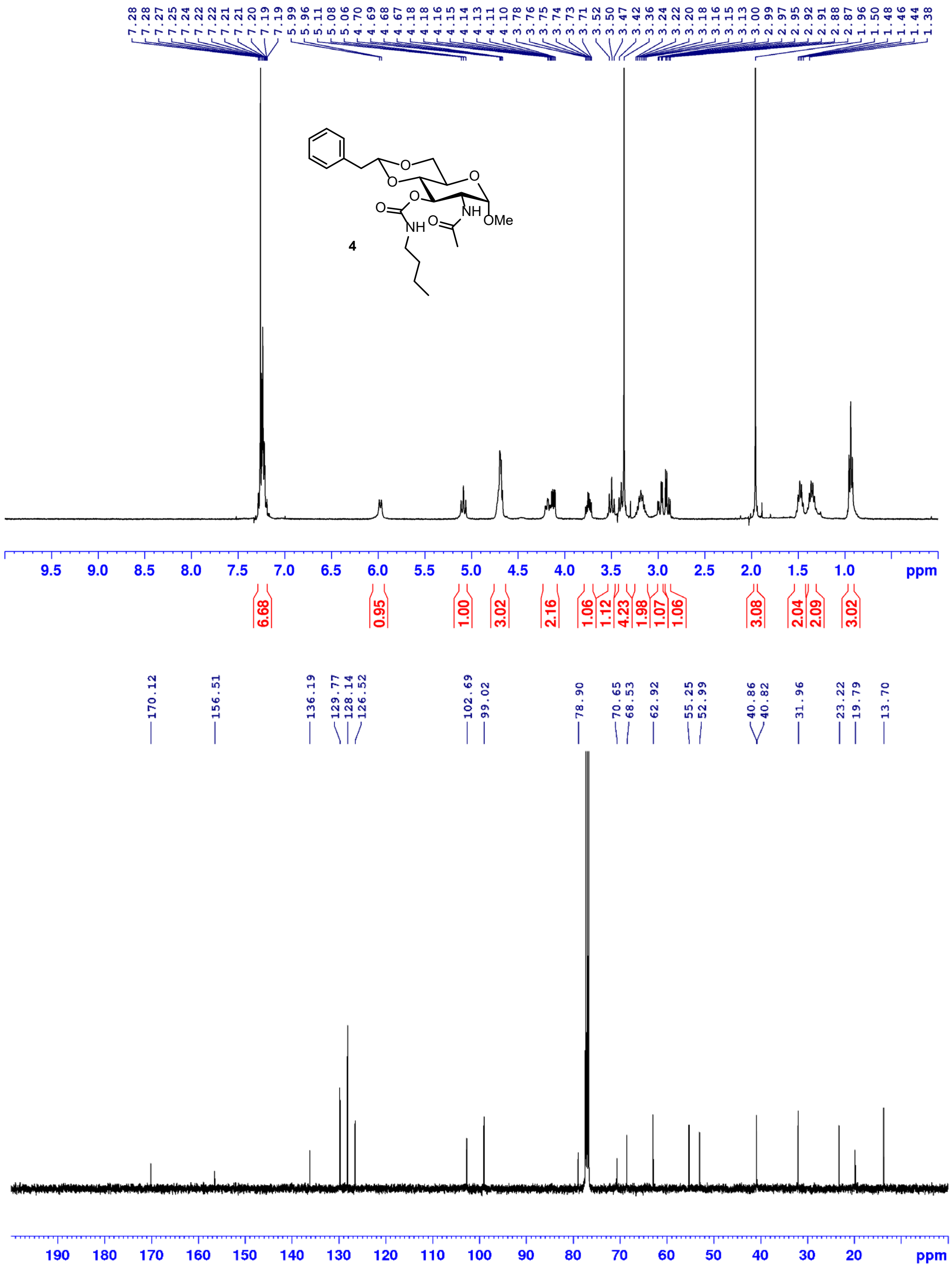

Figure S3. ^{1}H NMR and ^{13}C NMR spectra for compound **4** in $CDCl_3$

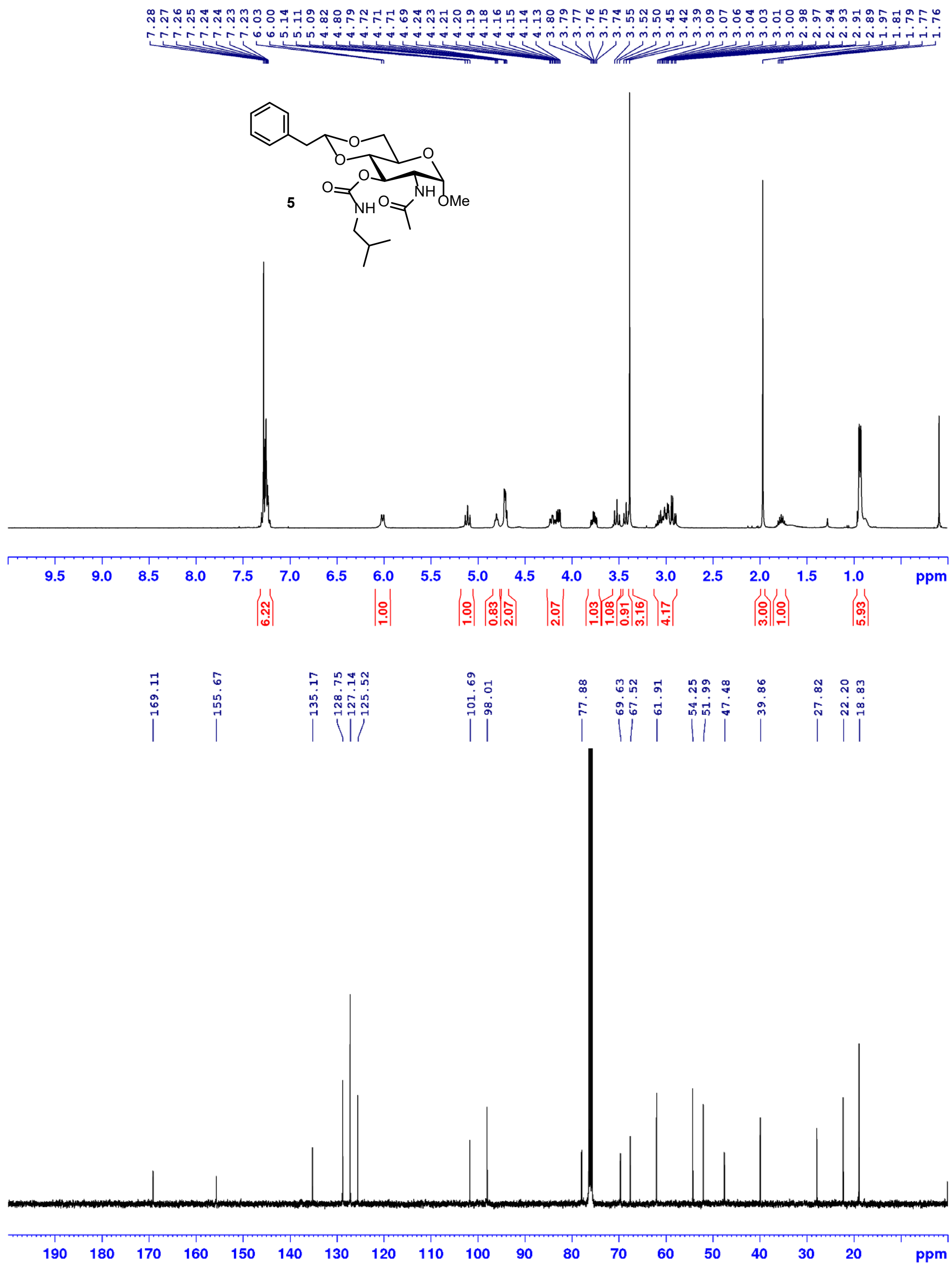

Figure S4. ^{1}H NMR and ^{13}C NMR spectra for compound **5** in $CDCl_3$

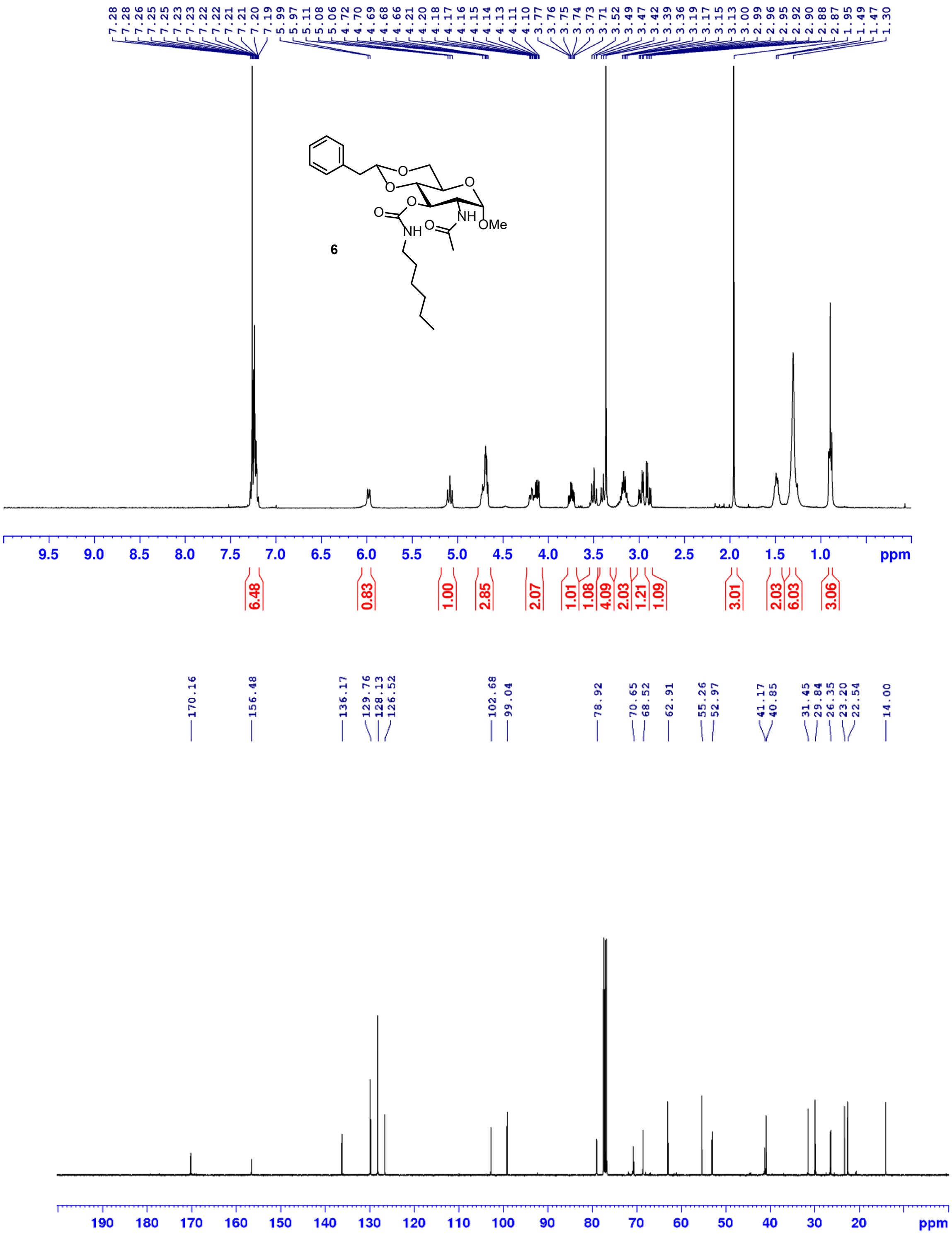

Figure S5. ^{1}H NMR and ^{13}C NMR spectra for compound **6** in $CDCl_3$

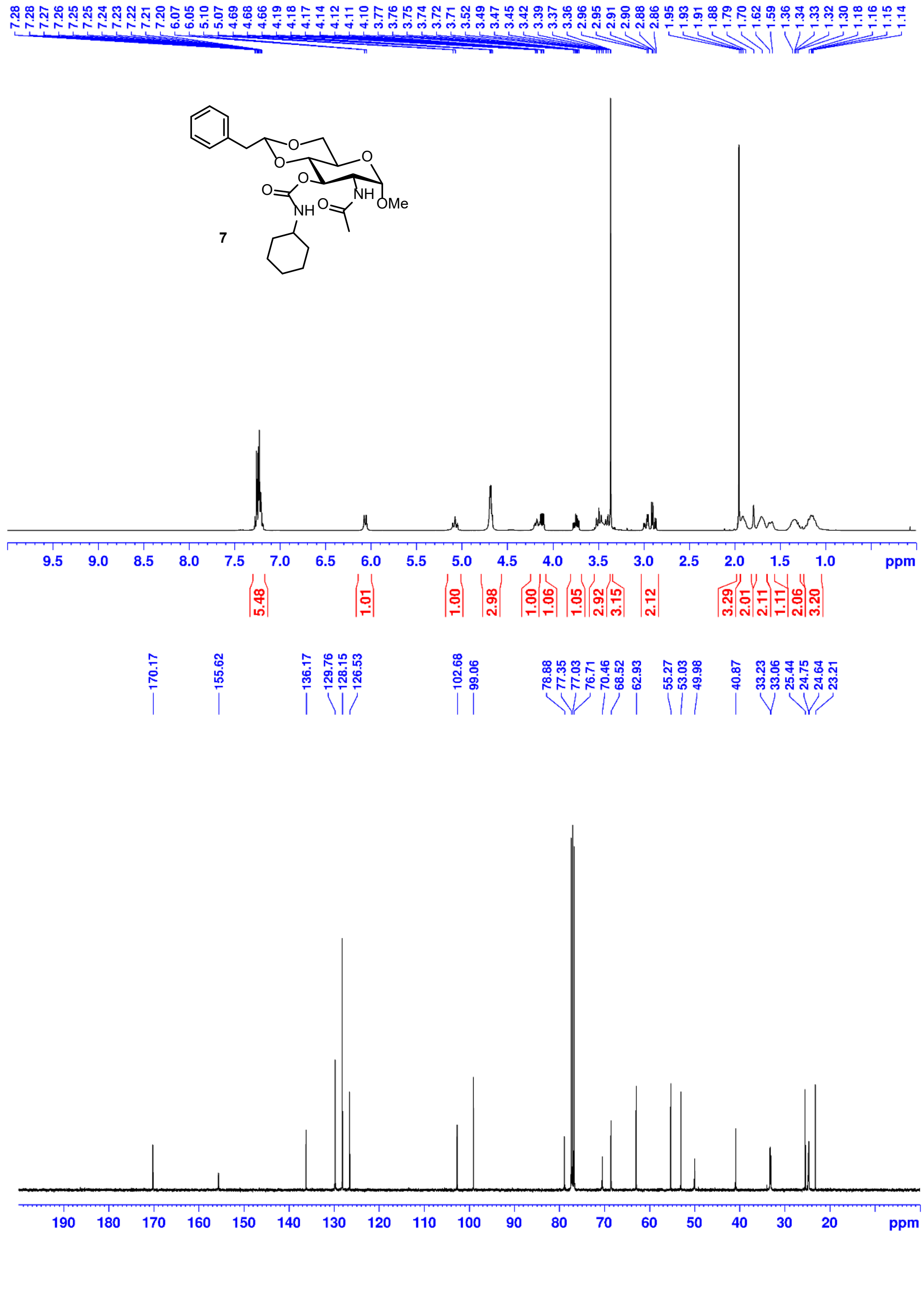

Figure S6. ^{1}H NMR and ^{13}C NMR spectra for compound **7** in $CDCl_3$

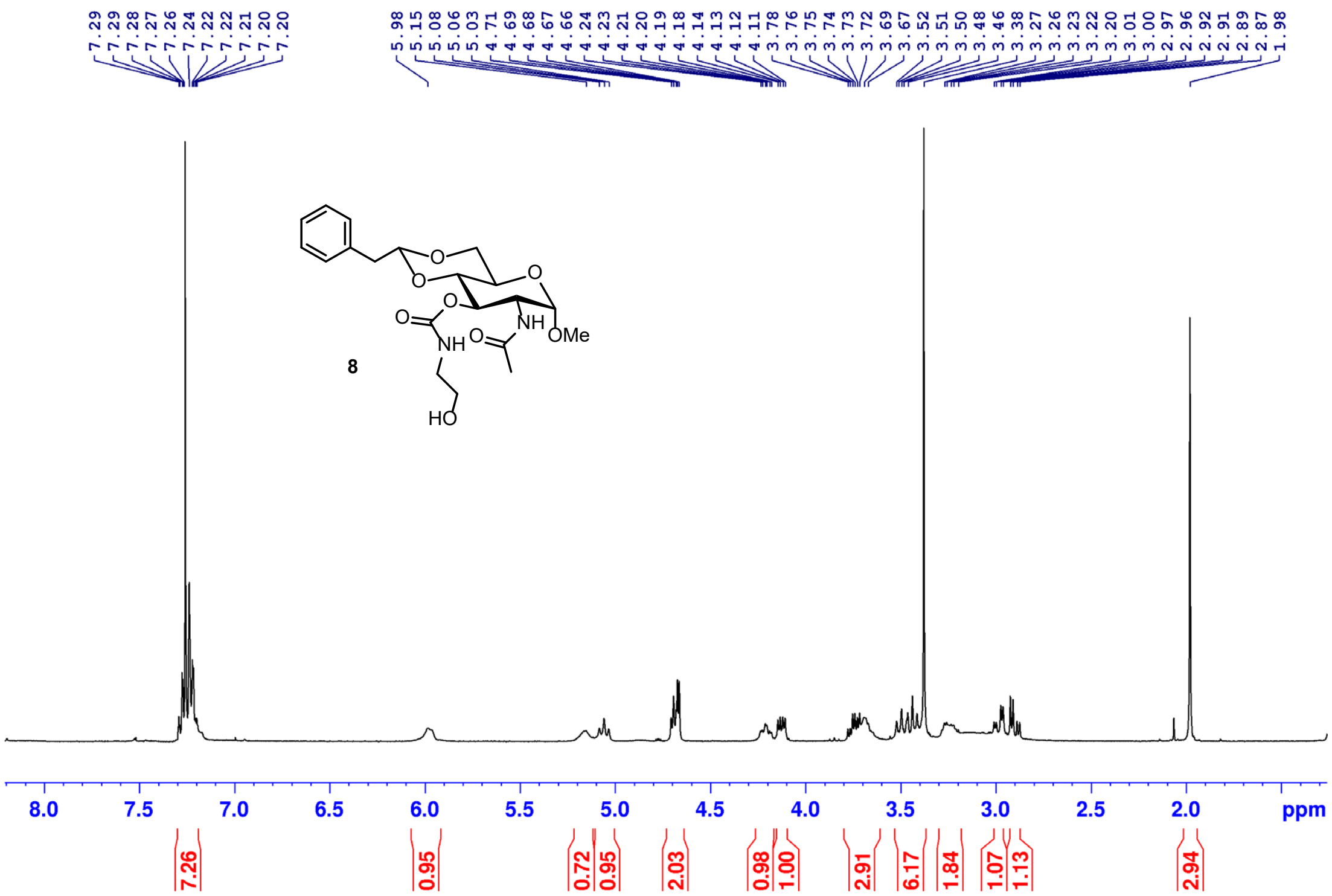

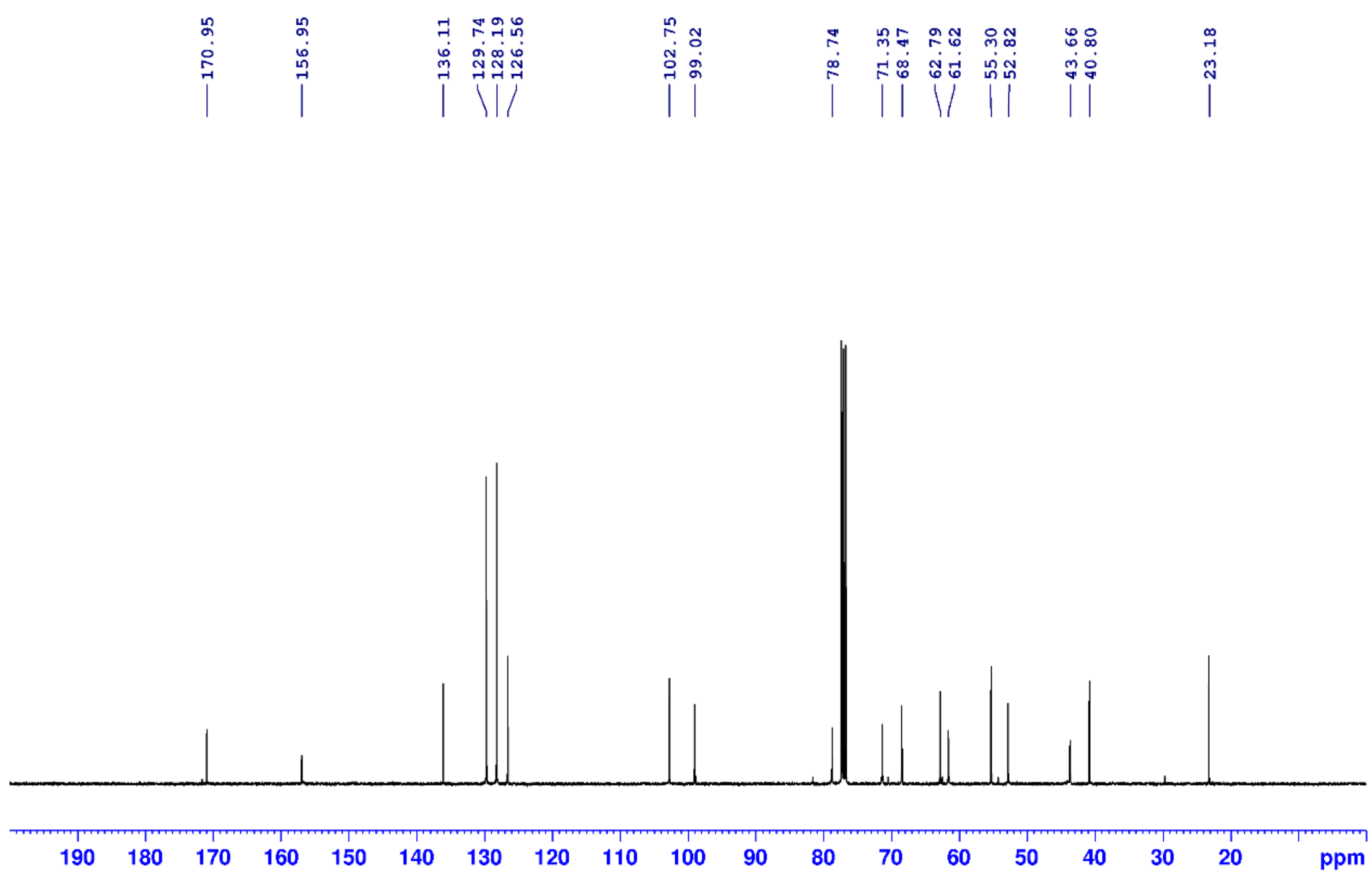

Figure S7. ^{1}H NMR and ^{13}C NMR spectra for compound **8** in $CDCl_3$

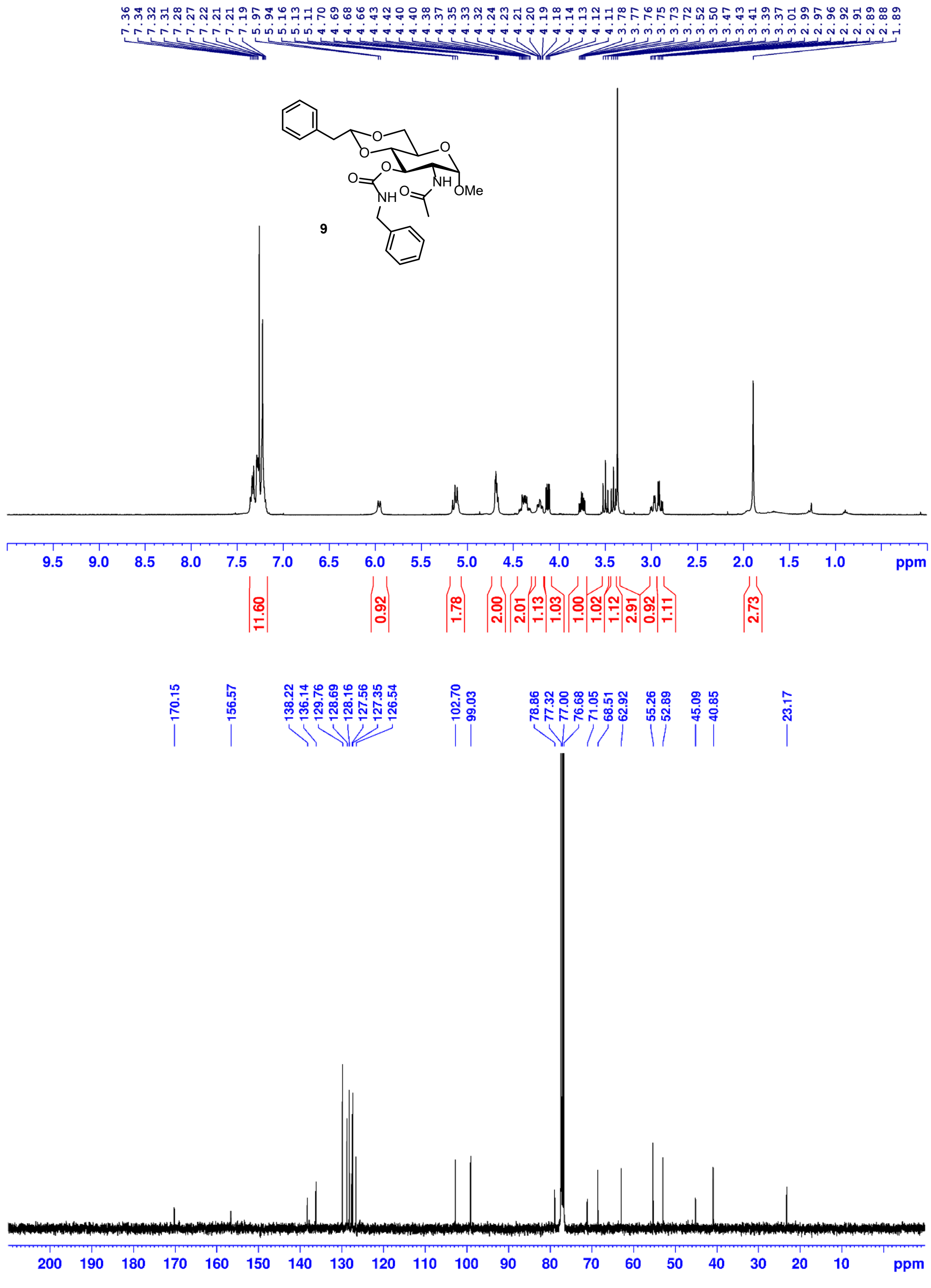

Figure S8. ^{1}H NMR and ^{13}C NMR spectra for compound **9** in $CDCl_3$

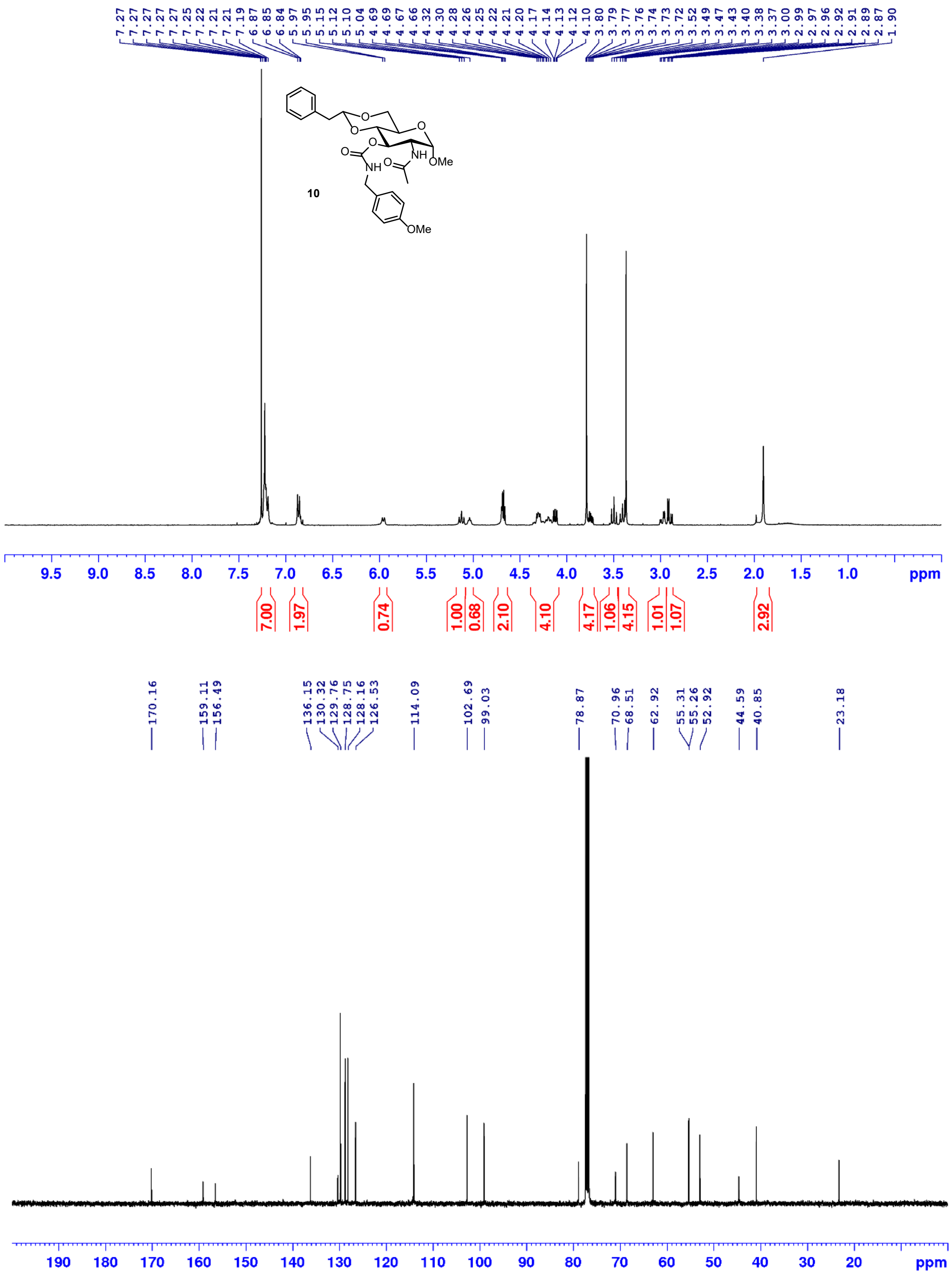

Figure S9. ^{1}H NMR and ^{13}C NMR spectra for compound **10** in $CDCl_3$

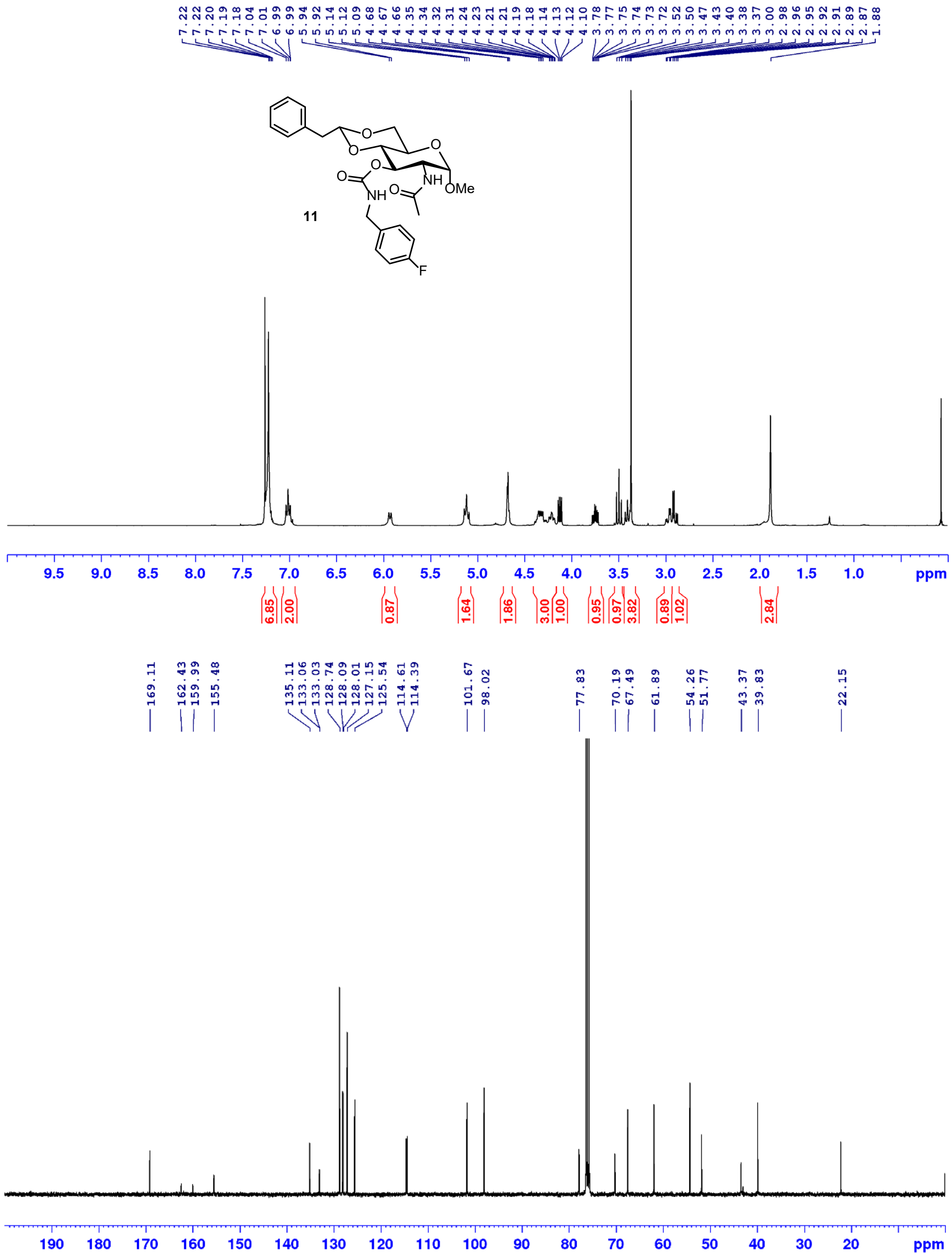

Figure S10. ^{1}H NMR and ^{13}C NMR spectra for compound **11** in $CDCl_3$

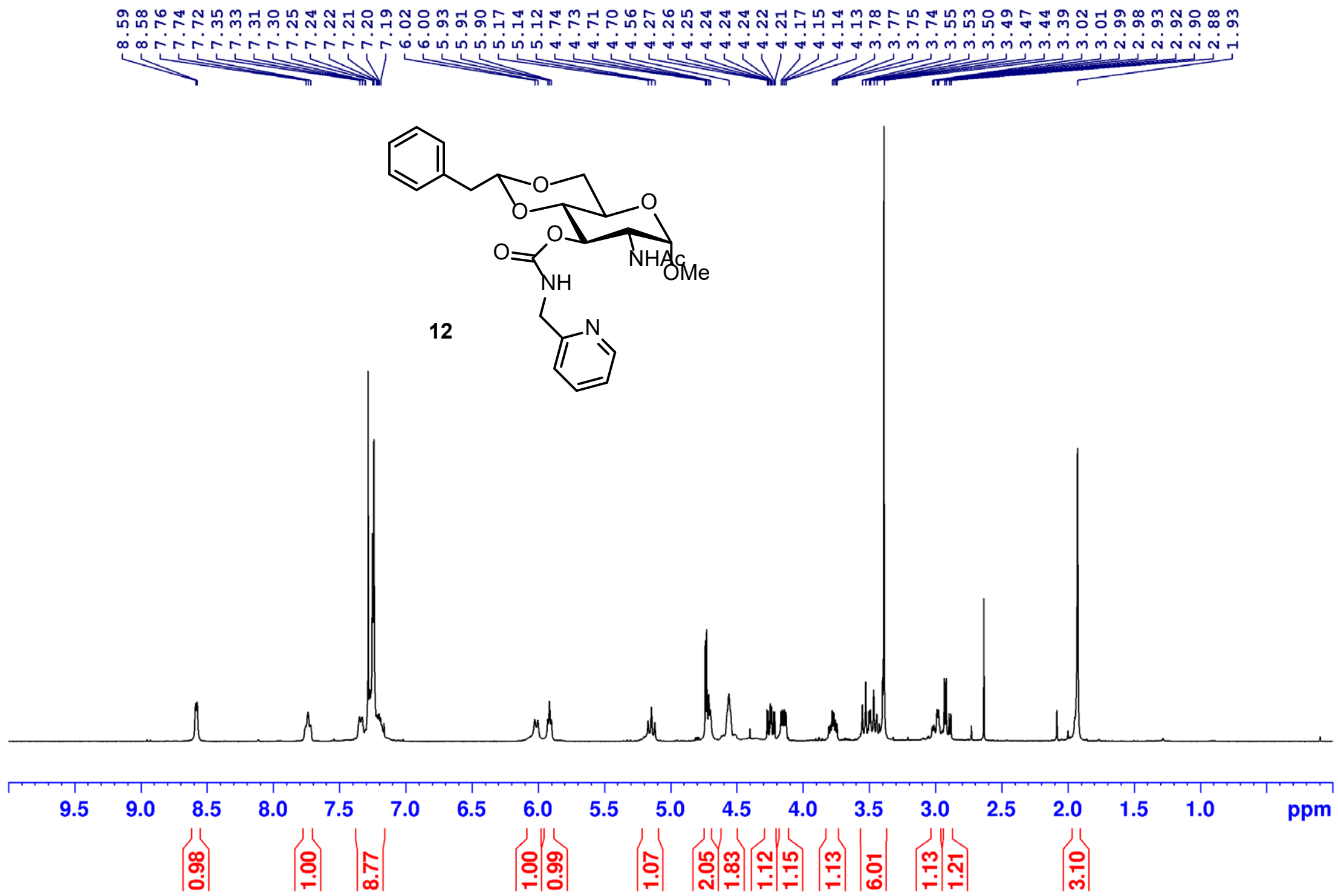

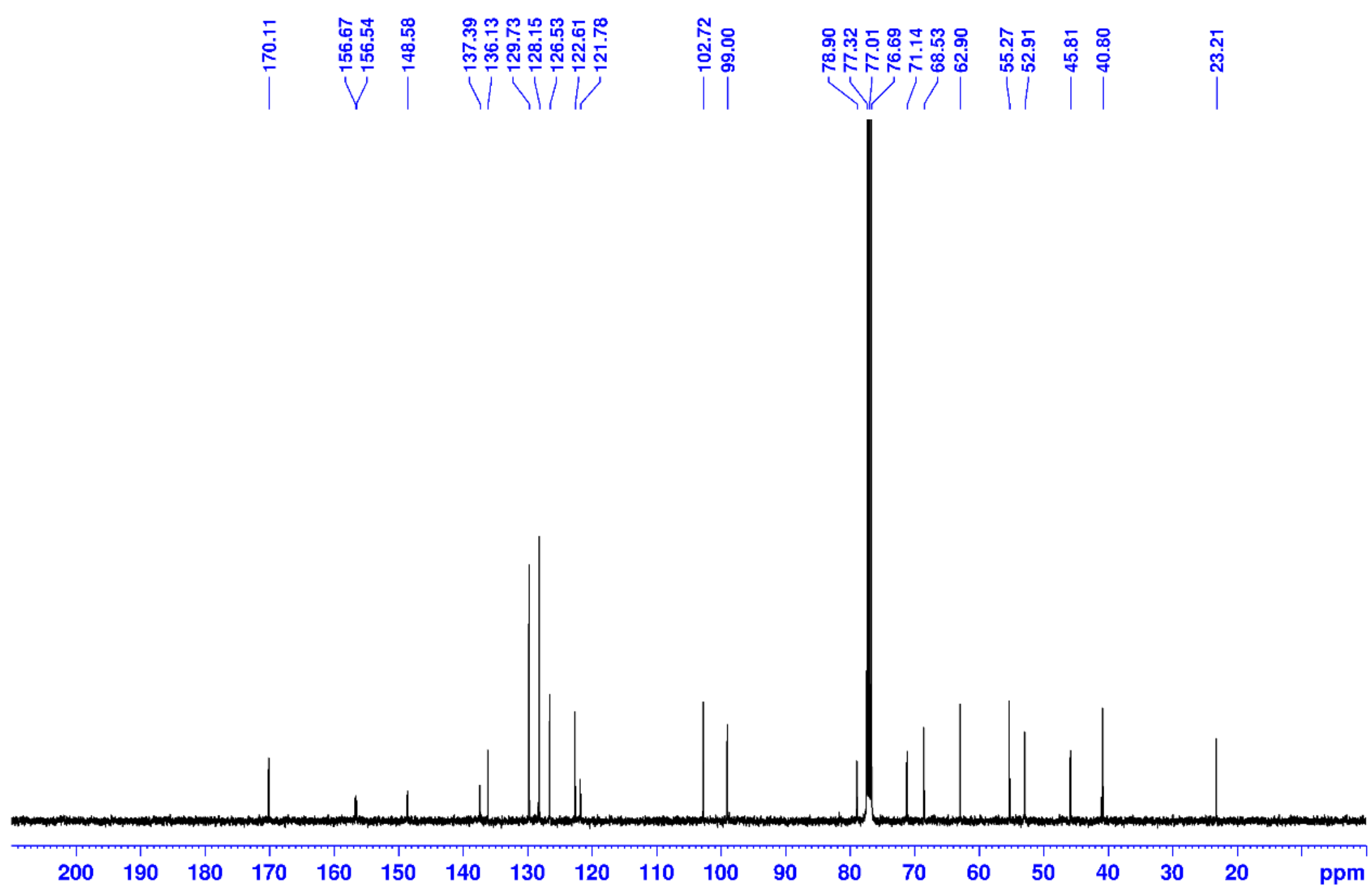

Figure S11. ^{1}H NMR and ^{13}C NMR spectra for compound **12** in $CDCl_3$

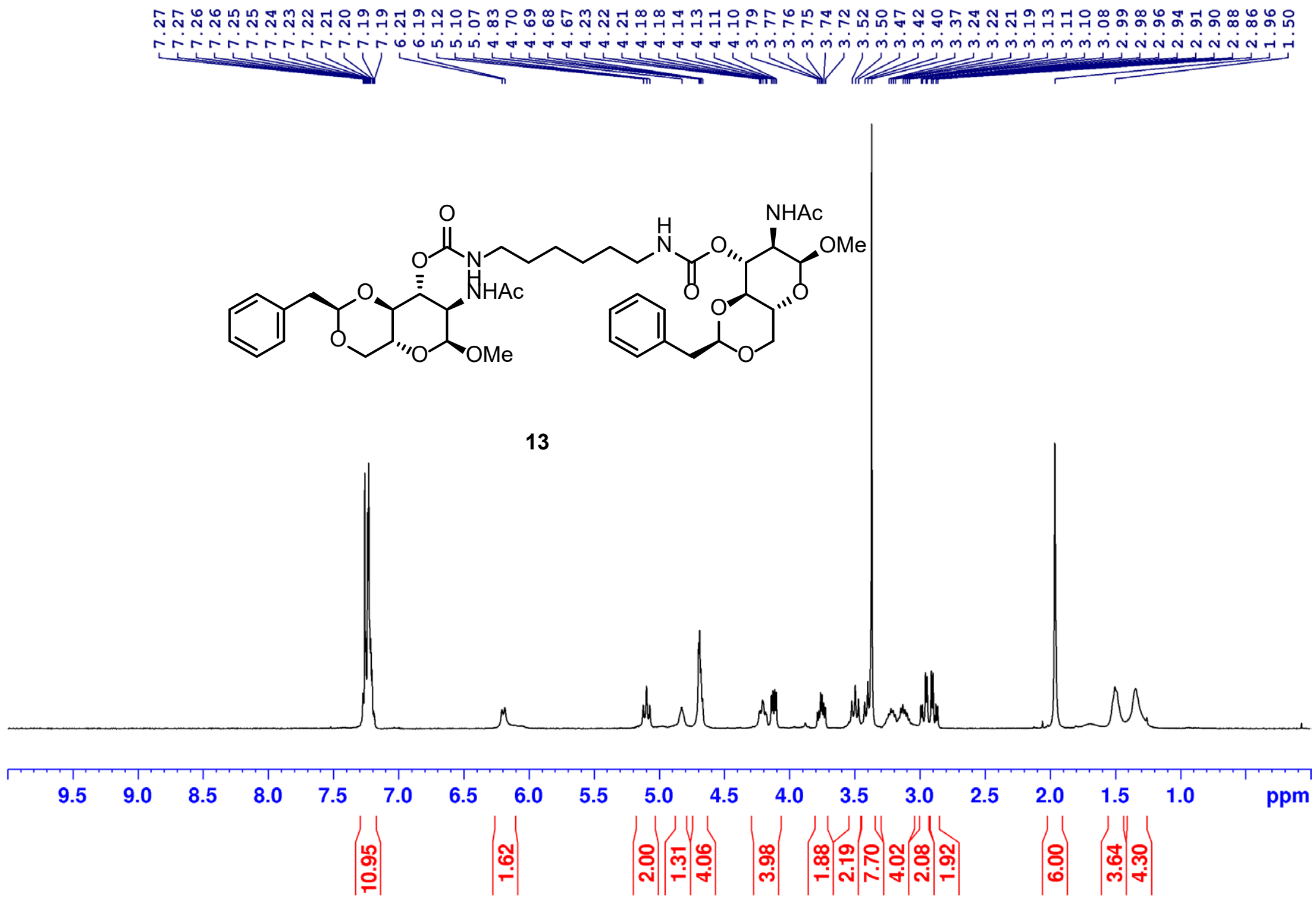

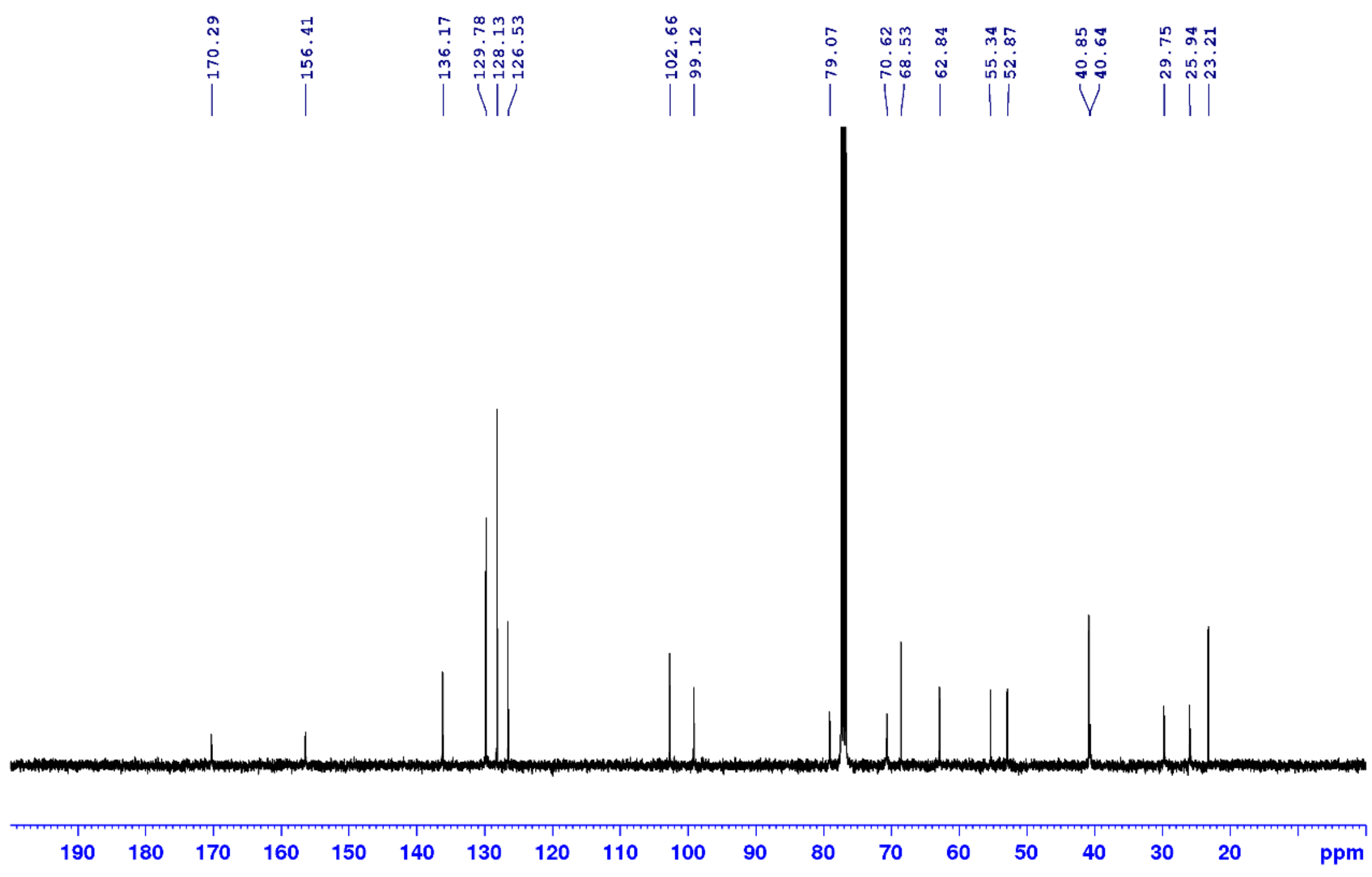

Figure S12. ^{1}H NMR and ^{13}C NMR spectra for compound **13** in $CDCl_3$

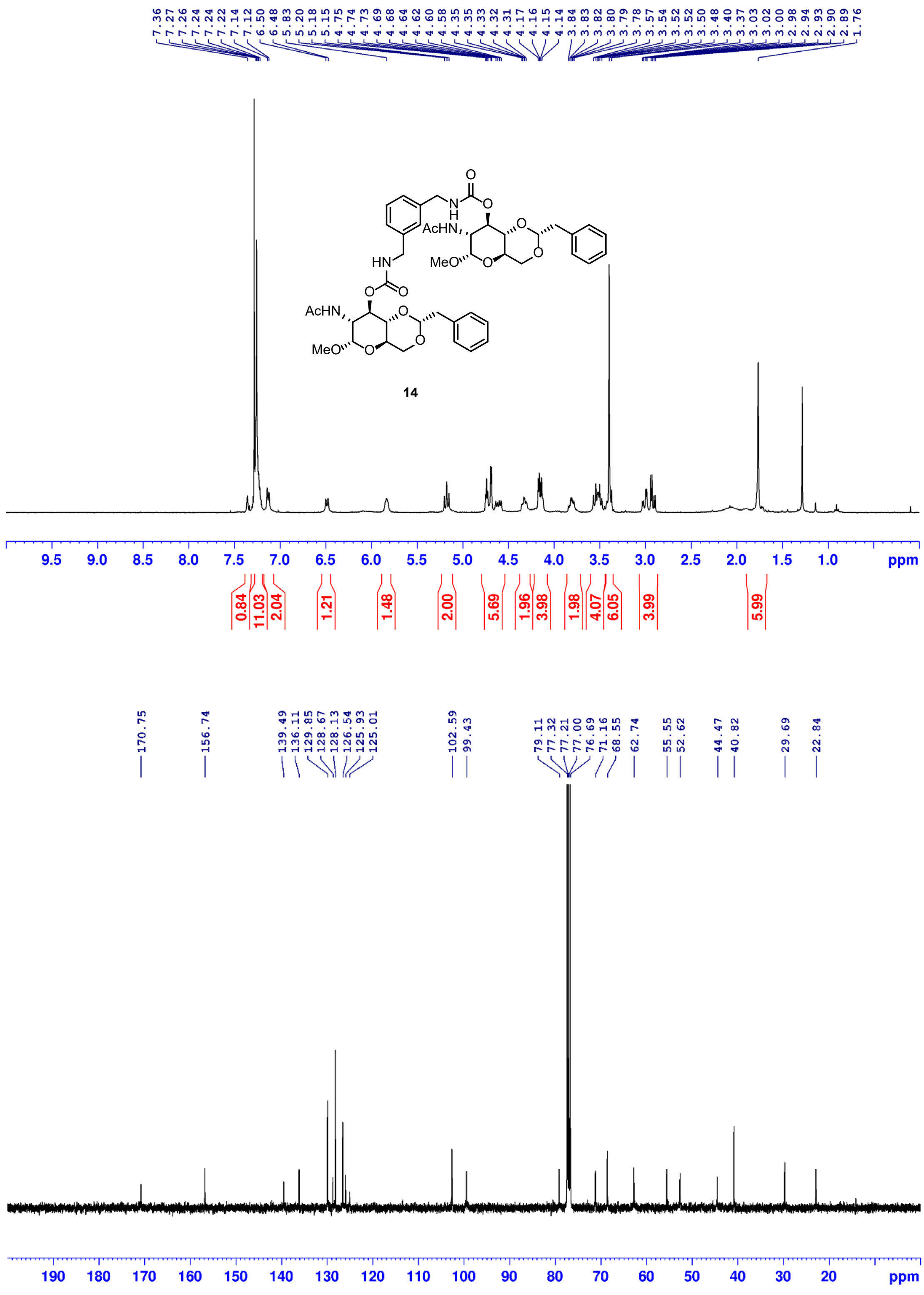

Figure S13. ^{1}H NMR and ^{13}C NMR spectra for compound **14** in $CDCl_3$

2. 2D NMR spectra of compounds **5** and **7**

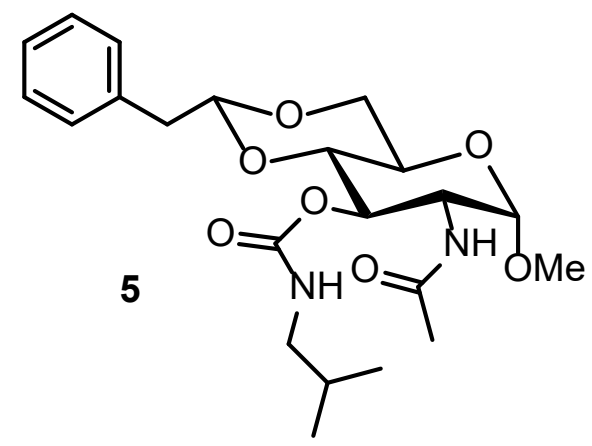

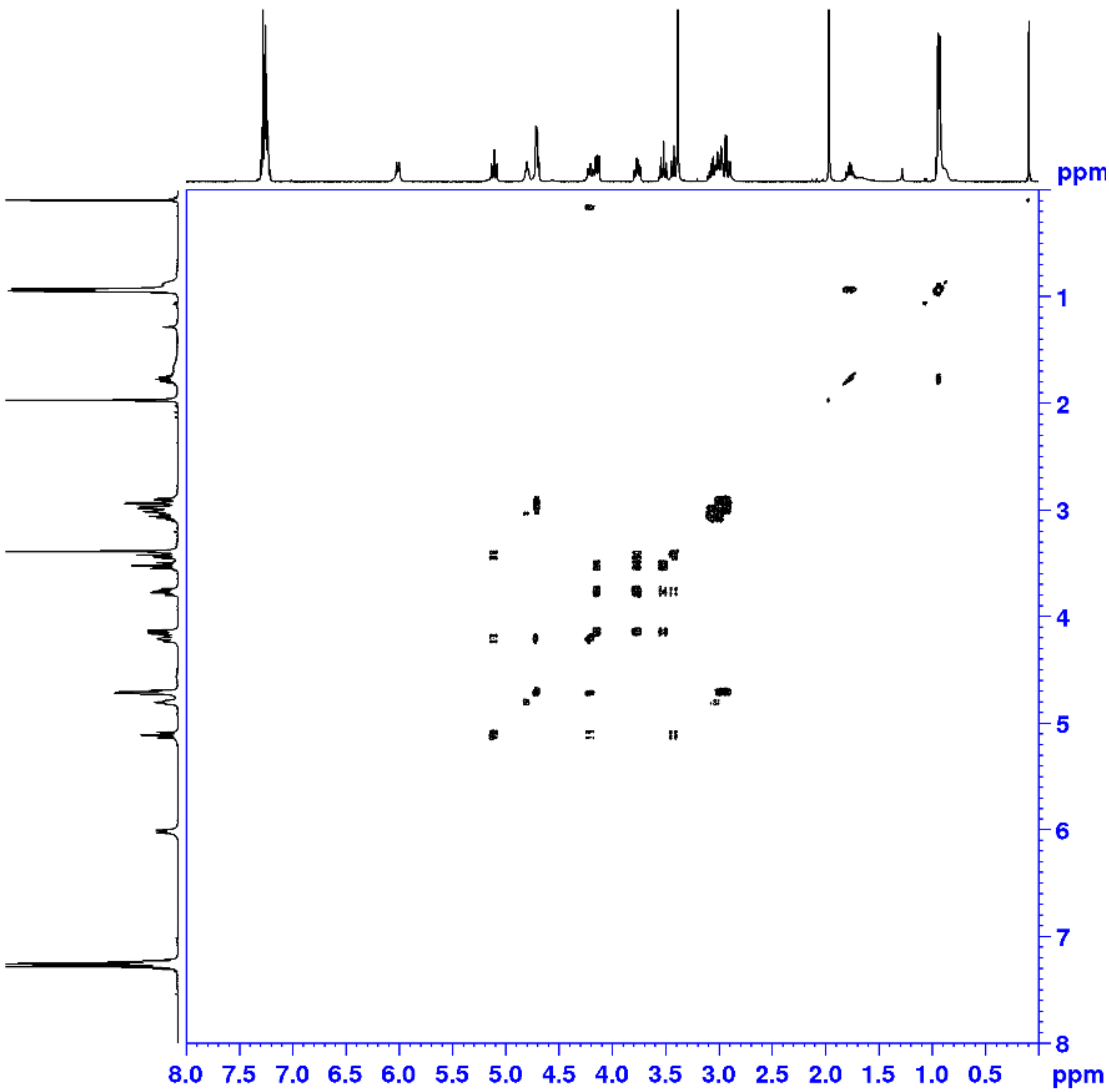

Figure S14. COSY NMR spectra for compound **5** in $CDCl_3$

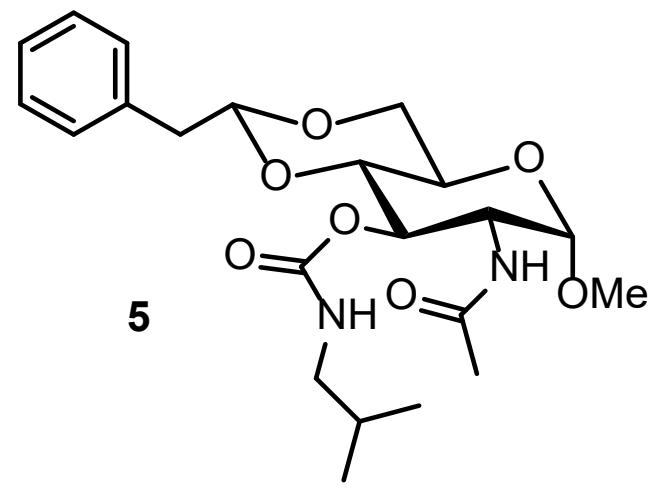

5

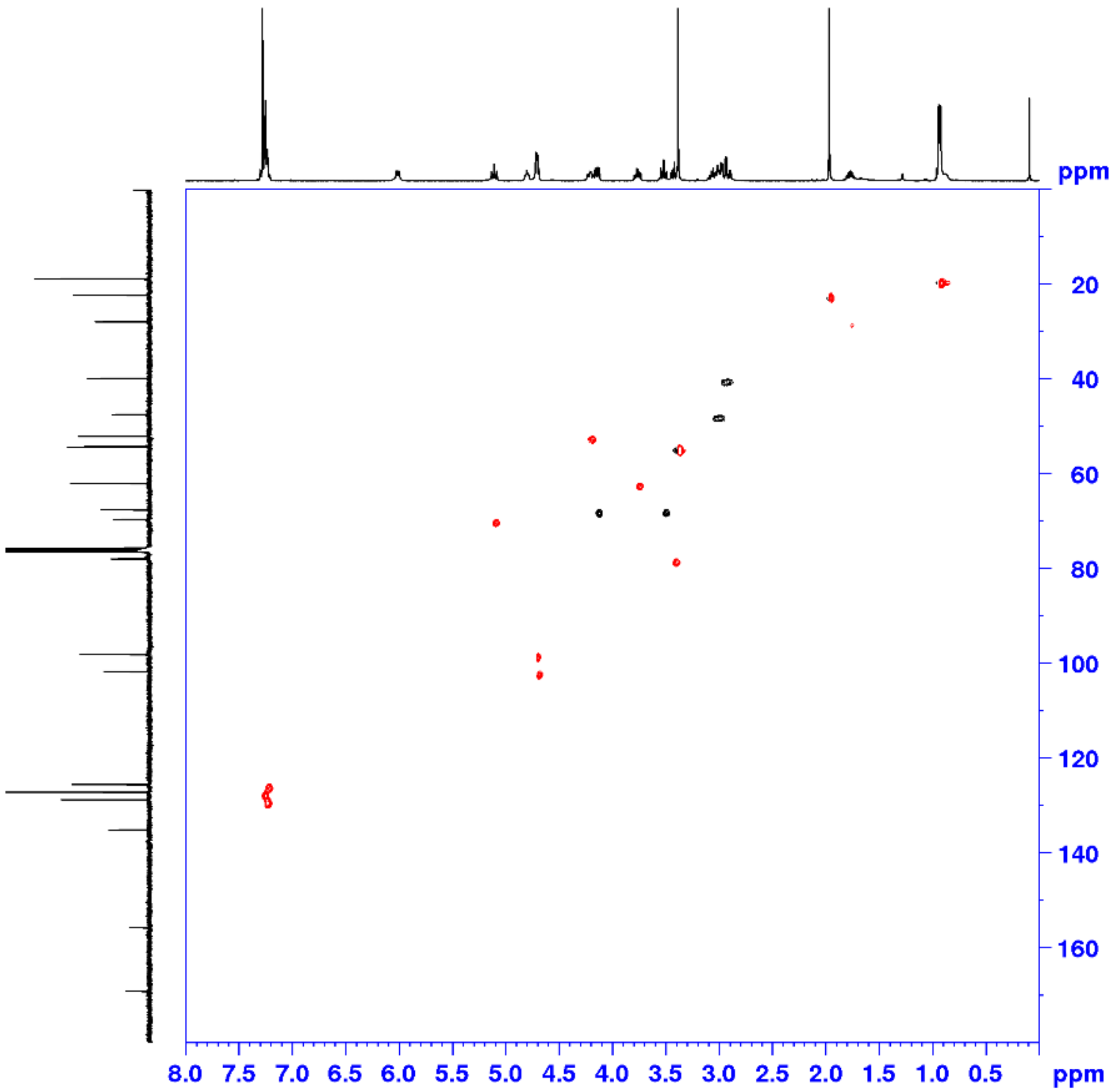

Figure S15. HSQC NMR spectra for compound **5** in $CDCl_3$

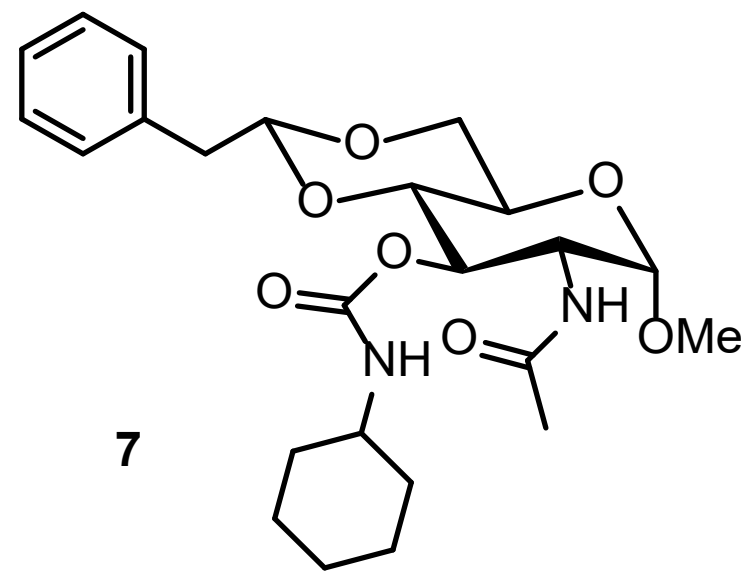

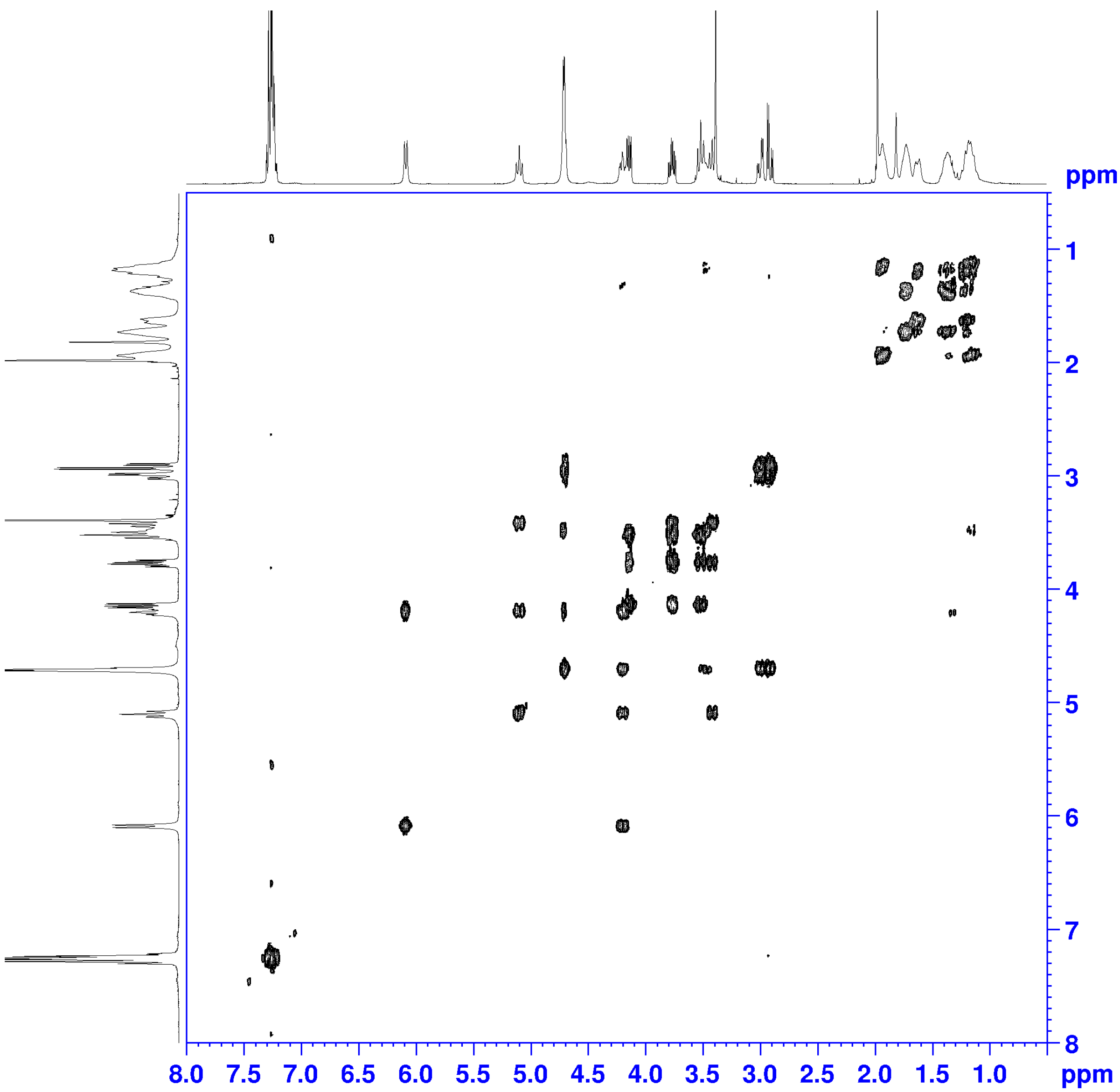

Figure S16. COSY NMR spectra for compound **7** in $CDCl_3$

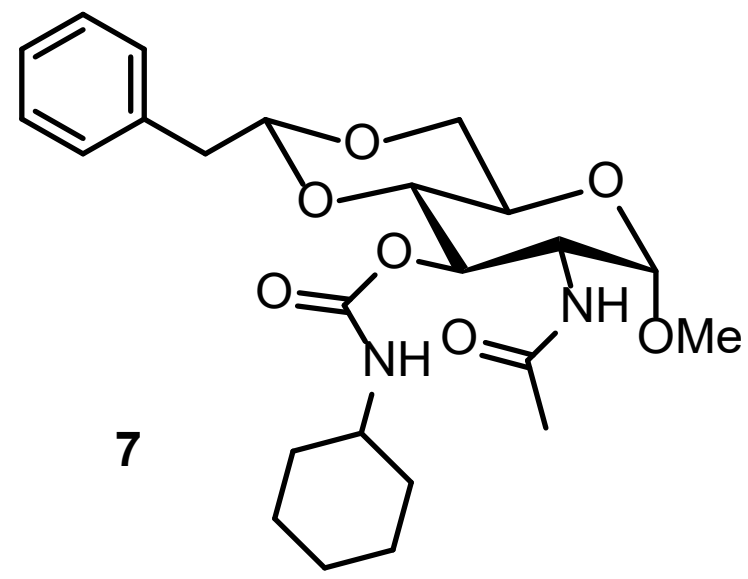

7

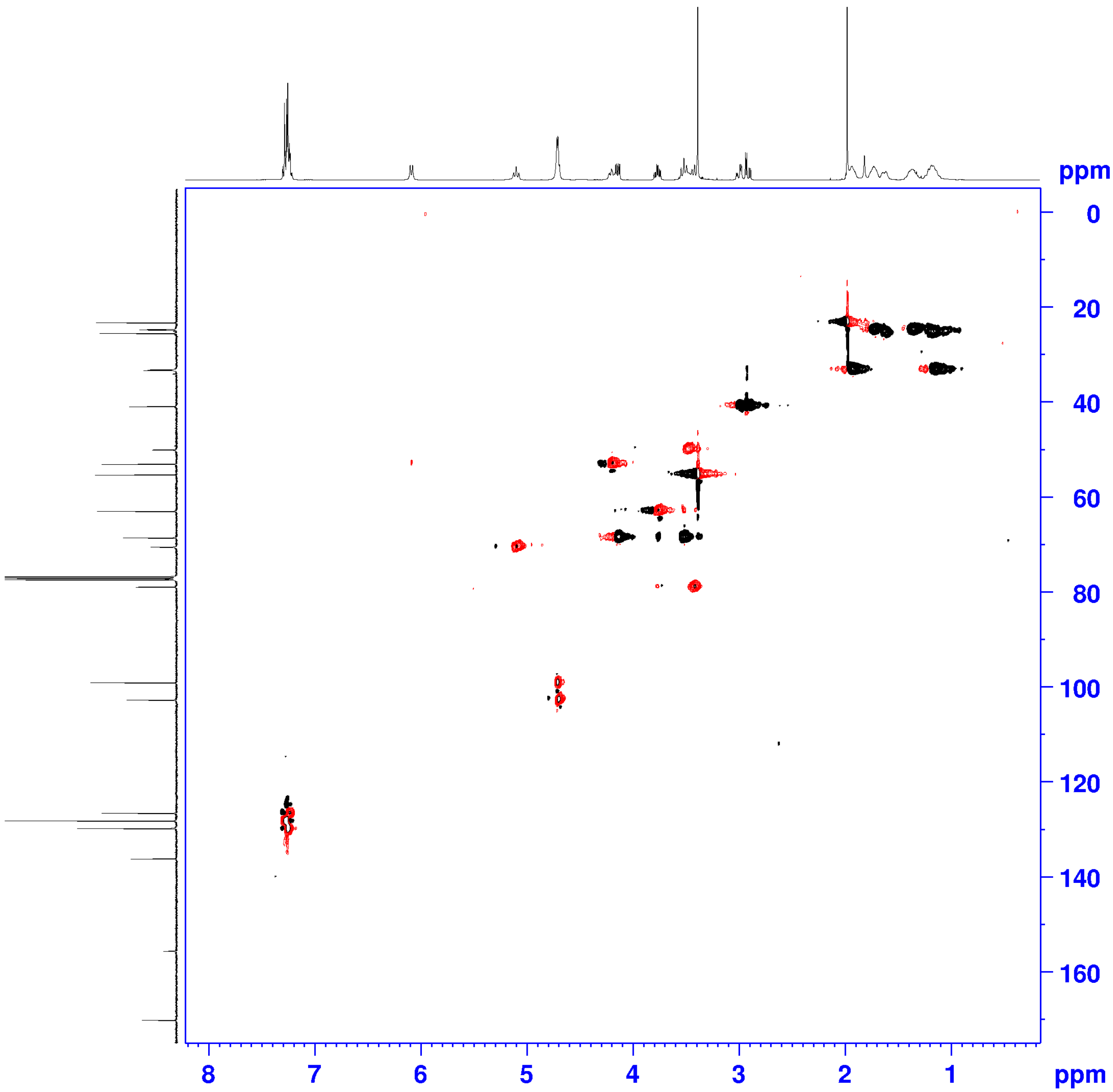

Figure S17. COSY and HSQC NMR spectra for compound **7** in $CDCl_3$

II. Rheological properties of selected gels

The amplitude and frequency sweep data are included for each gel in the following section. The storage modulus and loss modulus, as well as the ratio of G'/G", are included in a table following the graphs.

1. Rheology data for compound **7** in DMSO: H_2O (1:2) at 1.8 mg/mL

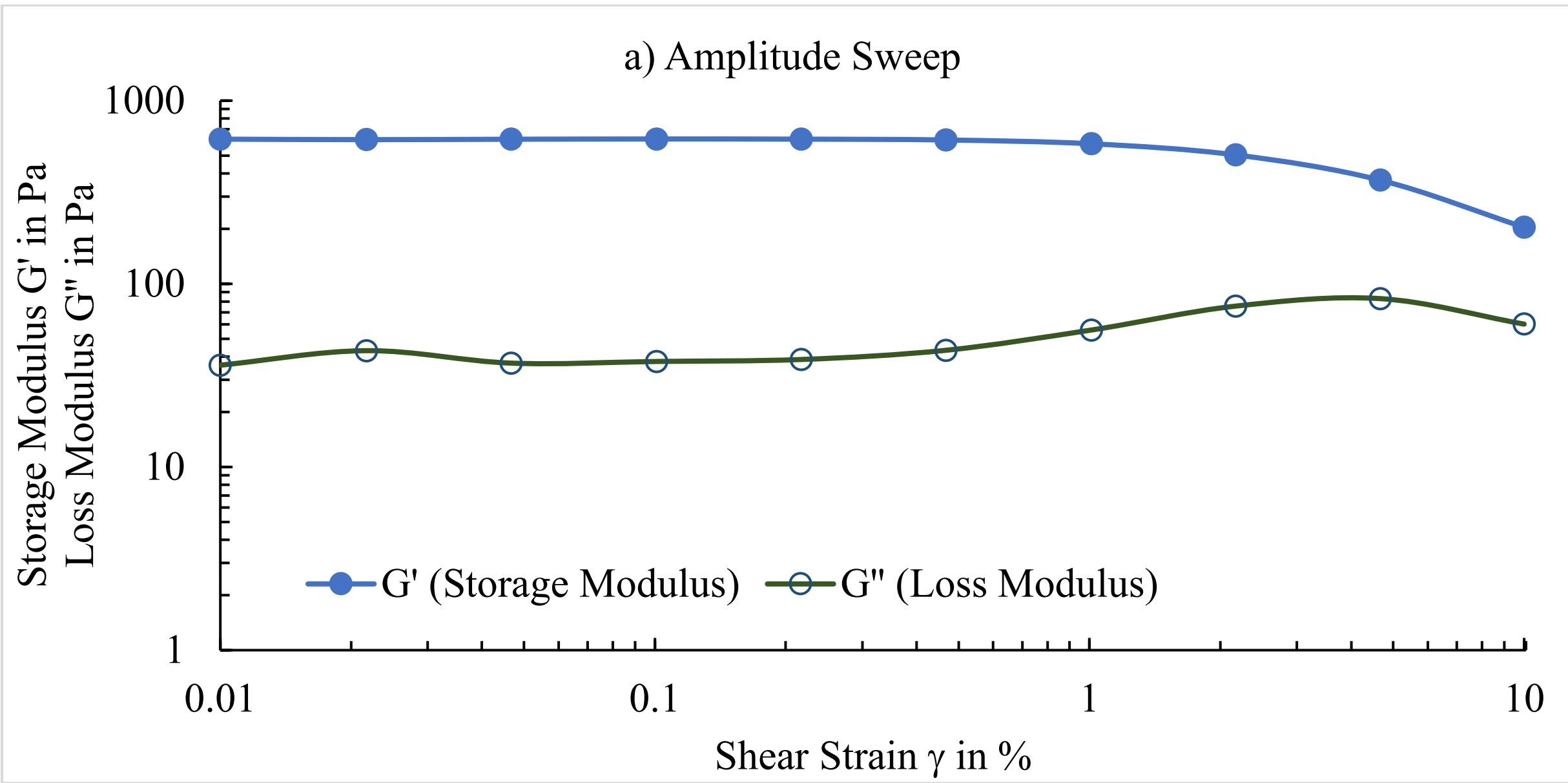

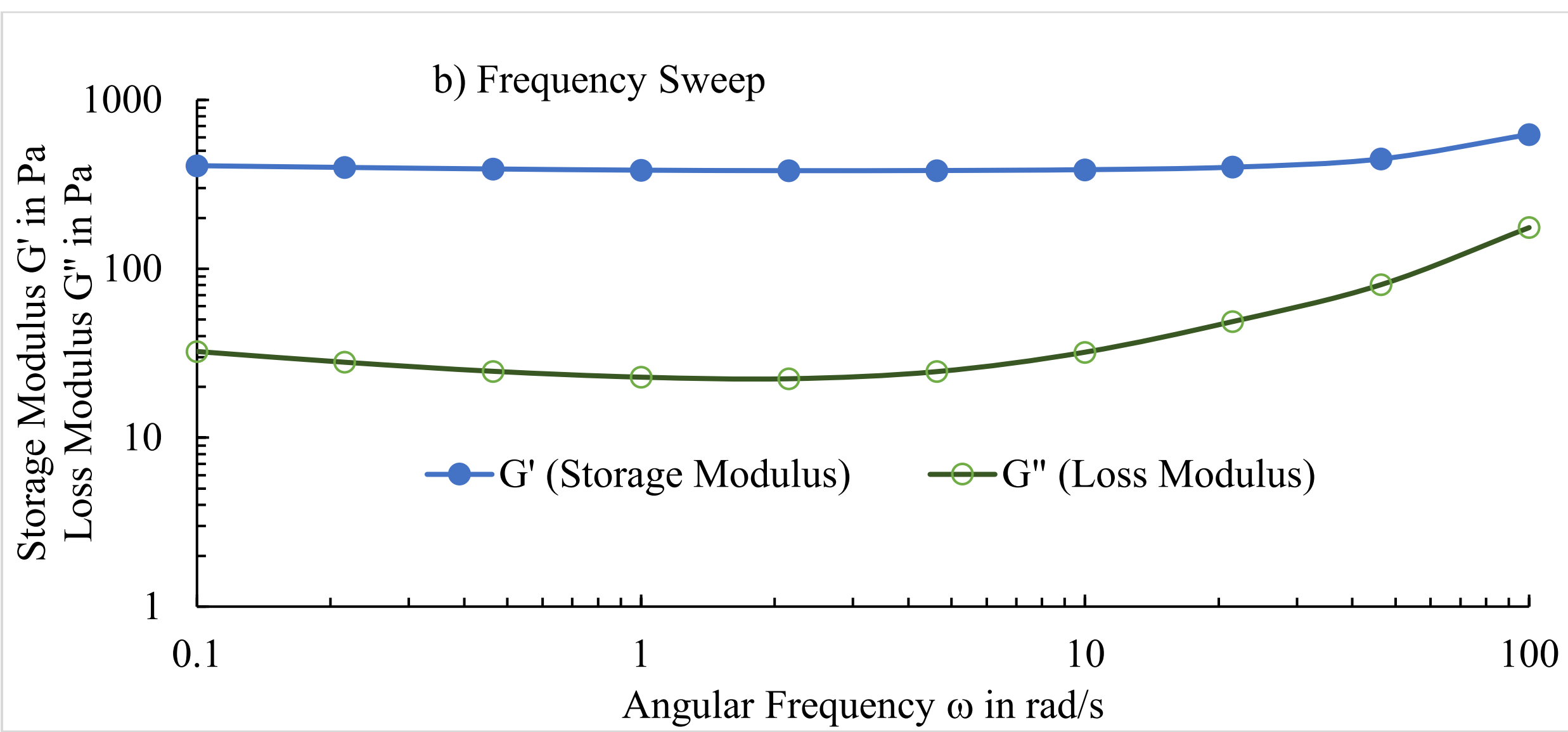

Figure S18. Amplitude sweep (a) and frequency sweep (b) for the gel formed by compound **7** at 1.8 mg/mL in DMSO: H_2O (1:2).

2. Rheology data for compound **7** in EtOH: H_2O (1:2) at 1.8 mg/mL

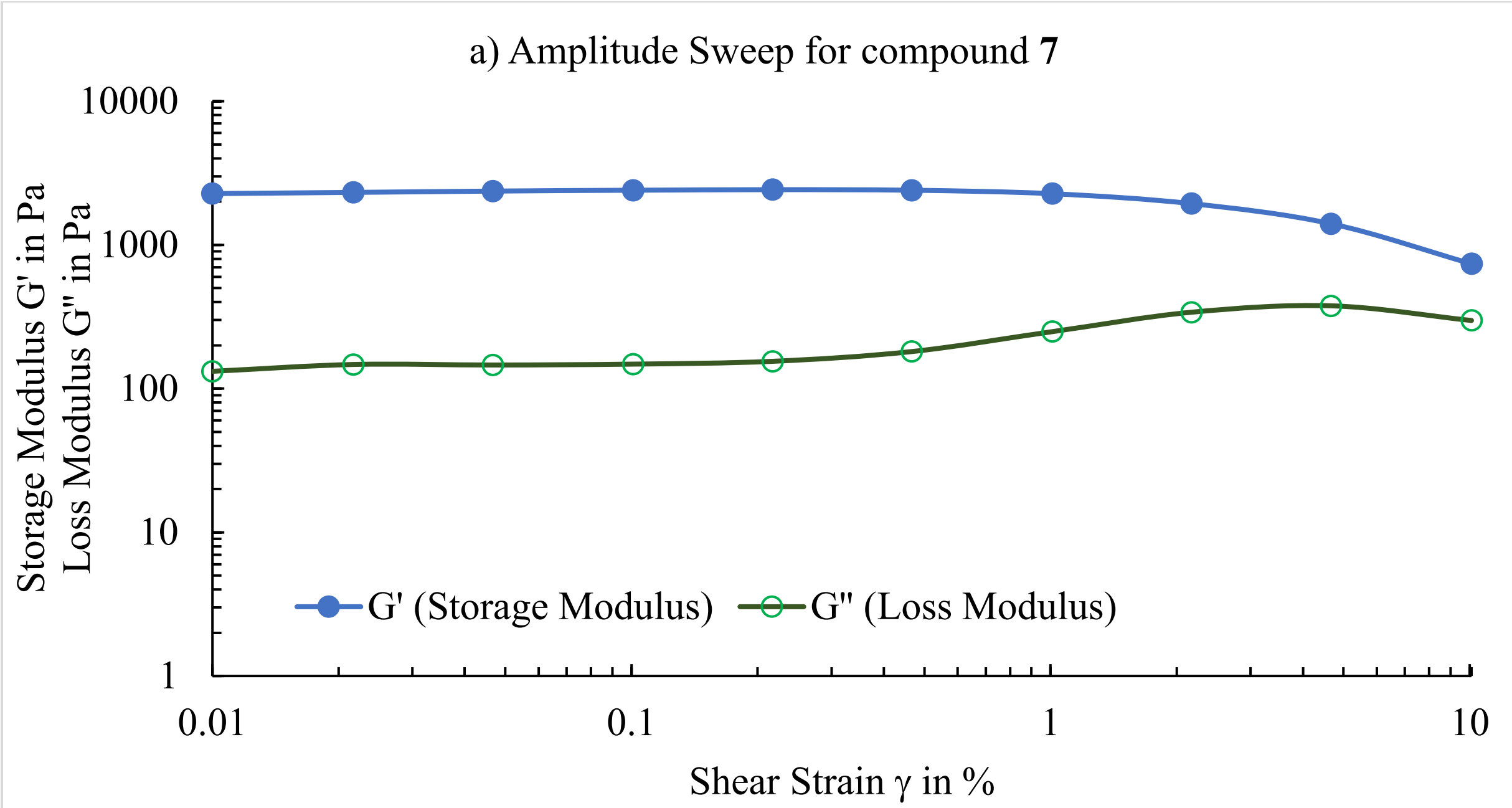

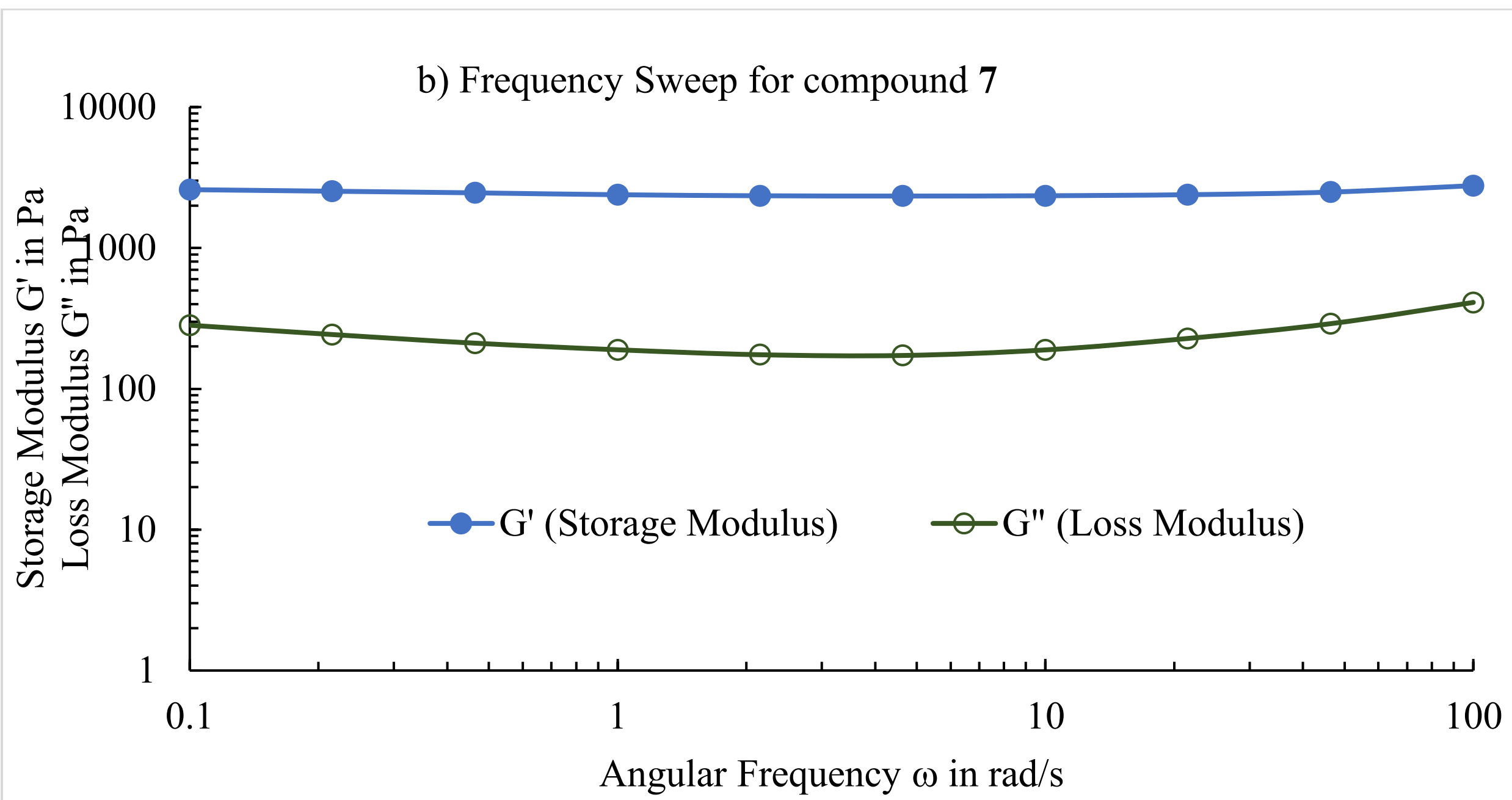

Figure S19. Amplitude sweep (a) and frequency sweep (b) for the gel formed by compound **7** at 1.8 mg/mL in EtOH: H_2O (1:2).

3. Rheology data for compound **7** in EtOH: H_2O (1:1) at 1.6 mg/mL

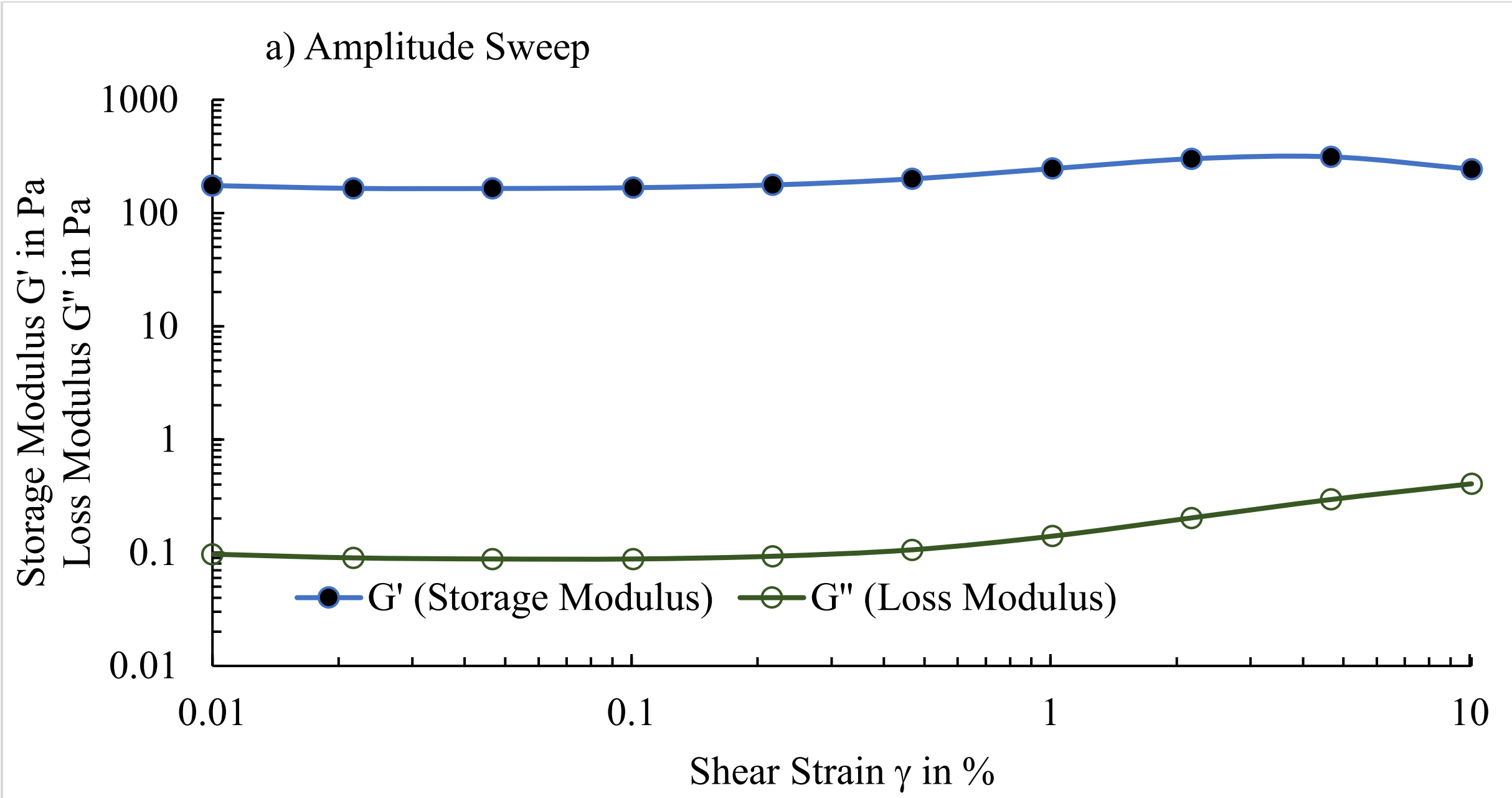

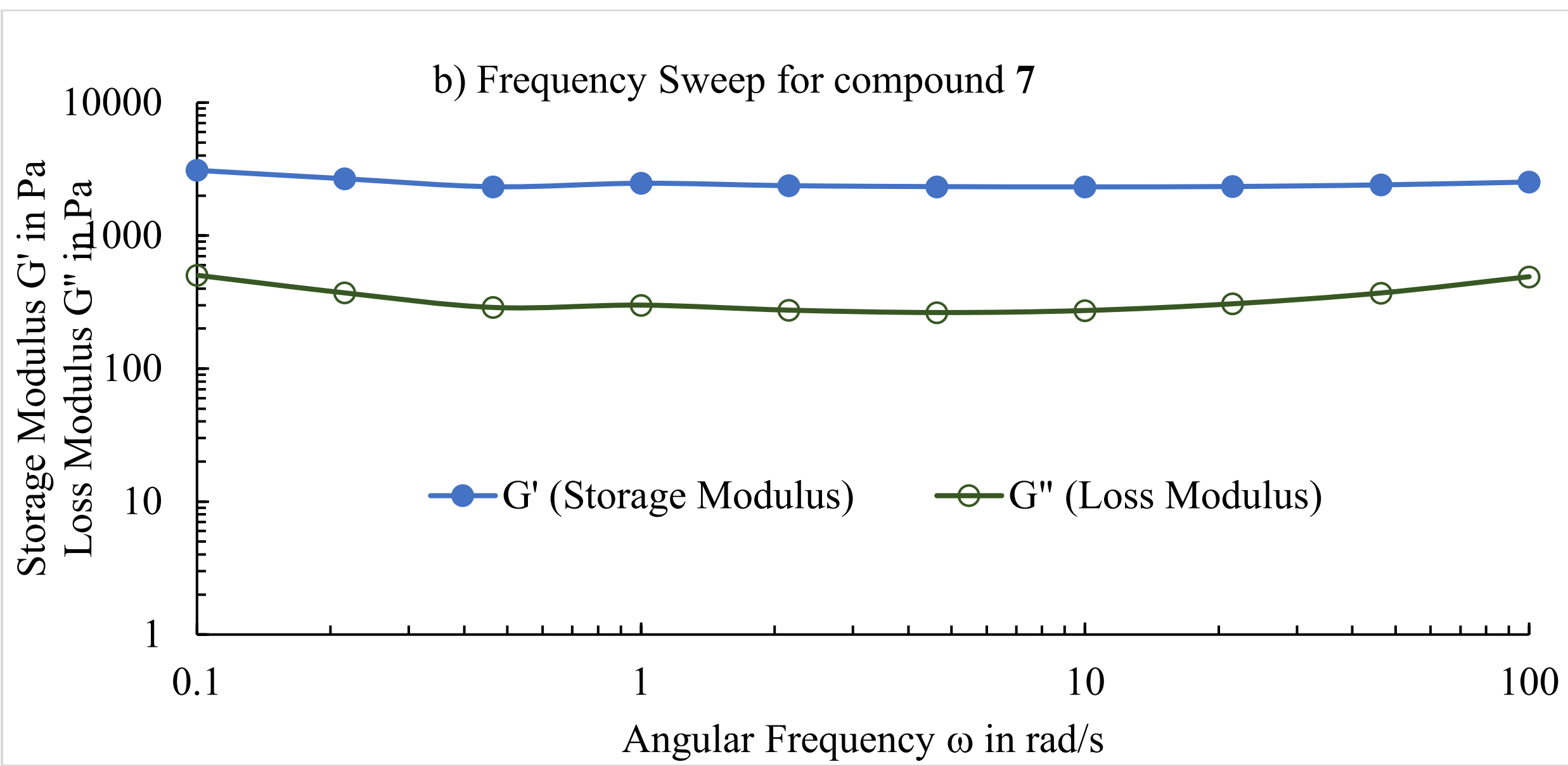

Figure S20. Amplitude sweep (a) and frequency sweep (b) for the gel formed by compound **7** at 1.6 mg/mL in EtOH: H_2O (1:1).

Table S1. The G' and G", and the ratio of G'/G" for the gel prepared from compound **7** at 1.8 mg/mL in DMSO: H_2O (1:2). The average was calculated using the range of 0.1-10 [rad/s]

ω [rad/s]	G' (Storage Modulus)	G" (Loss Modulus)	G'/G"
100.00	624.18	175.38	3.56
46.40	447.39	80.562	5.55
21.50	399.82	48.719	8.21
10.00	386.38	32.1	12.04
4.64	382.07	24.632	15.51
2.15	381.25	22.341	17.07
1.00	383.84	22.831	16.81
0.46	390.13	24.735	15.77
0.22	398.45	27.986	14.24
0.10	407.95	32.391	12.59
Average (G'/G") between 0.1 to 10 [rad/s]			14.9

Table S2. The G' and G", and ratio of G'/G" for the gel prepared from compound **7** in EtOH: H_2O (1:2) at 1.8 mg/mL

ω [rad/s]	G' (Storage Modulus)	G" (Loss Modulus)	G'/G"
100.00	2768.8	410.33	6.75
46.40	2491	289.92	8.59
21.50	2387.8	227.67	10.49
10.00	2347.1	188.86	12.43
4.64	2336.4	172.4	13.55
2.15	2346.2	174.53	13.44
1.00	2386.5	189.23	12.61
0.46	2460.7	210.8	11.67
0.22	2526.8	242.82	10.41
0.10	2593.2	282.71	9.17
Average (G'/G") 0.1 to 10 [rad/s]			11.9

Table S3. The G' and G", and ratio of G'/G" for the gel prepared from compound **7** in EtOH: H_2O (1:1) at 1.6 mg/mL

ω [rad/s]	G' (Storage Modulus)	G" (Loss Modulus)	G'/G"
100.00	2526.9	490.67	5.15
46.40	2404.9	370.33	6.49
21.50	2338.8	306.96	7.62
10.00	2323.5	273.21	8.50
4.64	2334.4	263.84	8.85
2.15	2373	275.03	8.63
1.00	2474.3	300	8.25
0.46	2329.9	288.14	8.09
0.22	2675.2	370.88	7.21
0.10	3094	502.99	6.15
Average (G'/G") 0.1 to 10 [rad/s]			8.0

III. Gelation test of compound 7 in DMSO/water and metallogels formation

1. Gelation test in DMSO/water mixtures

Since compound **7** was the best-performing gelator with low minimum gelation concentrations in aqueous mixtures of DMSO, a gelation test experiment in different ratios of DMSO: water was performed in order to obtain the minimum amount of DMSO required to form a hydrogel. Compound 7 (2.0 mg) was added to a vial, then 0.1 mL DMSO was added, and the gelator formed a homogenous solution. To this solution, 0.1 mL of water was added, resulting in a spontaneous gel. Continuing with the process by adding 0.1 mL of water sequentially, gel formation was tested again by a similar method. From this point onward, stable gels formed after reheating the gel and the analyte. The results are shown in Table S4, and the corresponding gel photos are shown in Figure S21.

Table S4. Gelation properties of compound **7** in different mixtures of DMSO and water

**DMSO:H_2O**	**Gelation properties**	**Spontaneous or require heating to reform?**	**Gelator Concentration (mg/mL)**
1:1	Go	Spontaneous	10
1:2	Go	Heat	6.7
1:3	Go	Heat	5.0
1:4	Go	Heat	4.0
1:5	Go	Heat	3.3
1:6	Go	Heat	2.9
1:7	Go	Heat	2.5
1:8	Go	Heat	2.2
1:9	Go	Heat	2.0
1:10	Go	Heat	1.8
1:11	UG	Heat	1.7
1:12	UG	Heat	1.5

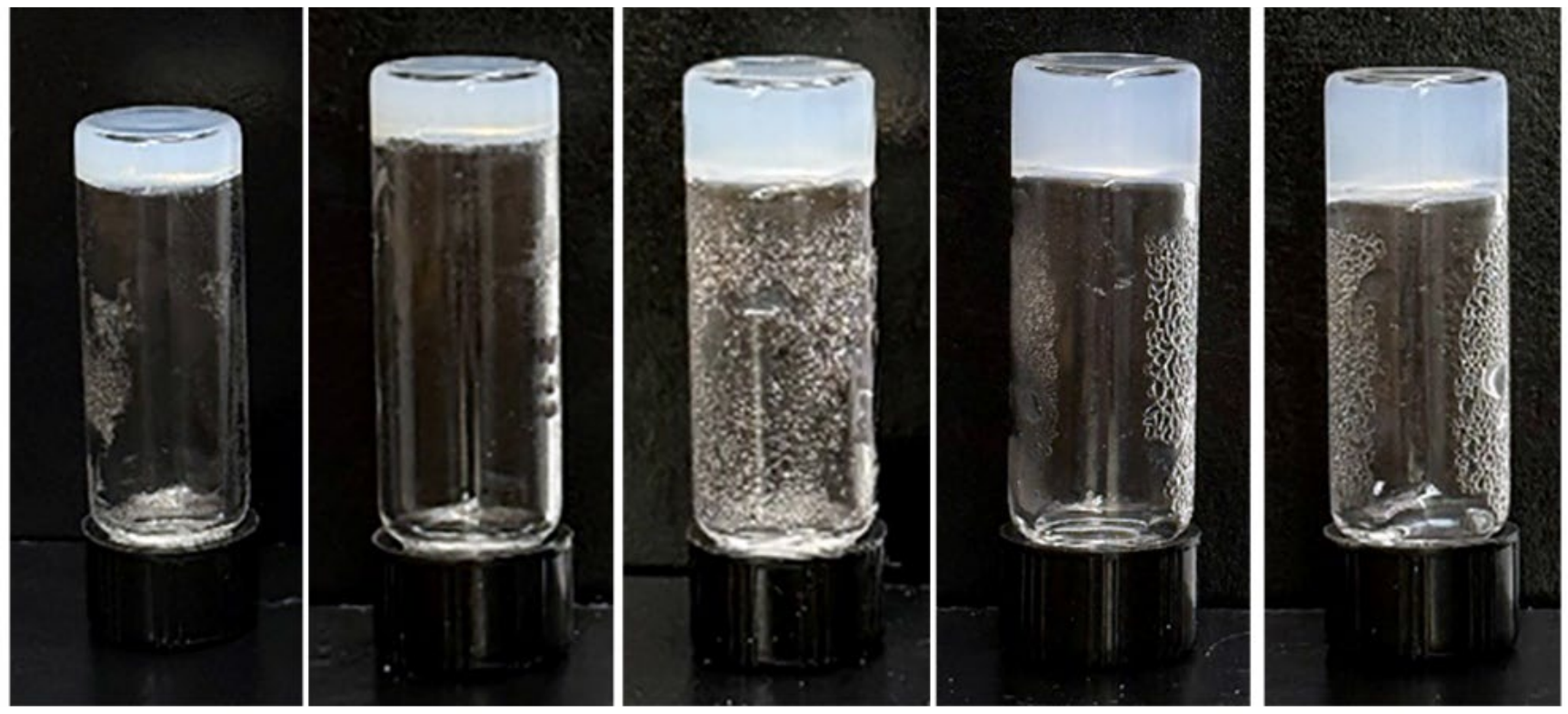

Figure S21. Selected gel photos of the gels formed at different DMSO and water volume ratios, DMSO:H_2O from 1:2 to 1:12.

2. Metallogel Test for Compound **7**

A stock metal salt solution was prepared for each of the following: $Zn(OAc)_2 \cdot 2H_2O$, $Hg(OAc)_2$, $Cu(OAc)_2 \cdot H_2O$, $FeCl_2$, and $NiCl_2 \cdot 6H_2O$. The solution was prepared at 0.5 equiv (2.0 mg, 1.0 eq) of compound **7** in Millipore water. Then 0.25 mL (0.5 eq) of the metallic stock solution was added to a 1 dram vial, followed by 2.0 mg of compound **7** and 0.5 mL DMSO:H_2O (v/v 1:3). The mixture was heated to dissolve the gelator and the salt. The samples were then left to cool at rt for 5-10 minutes, and observation of gelation was then recorded. All samples formed stable gels except $Hg(OA)_2$. These are shown in Figure S5. For those samples that stable gels were formed, another 0.25 mL of the metallic stock solution was added to the vial and reform the gel, the resulting gels are shown in Figure S6. The metal ion to gelator ratio is 1:1 molar ratio, the final solvent of the gel is composed of DMSO:H_2O (v/v 1:7).

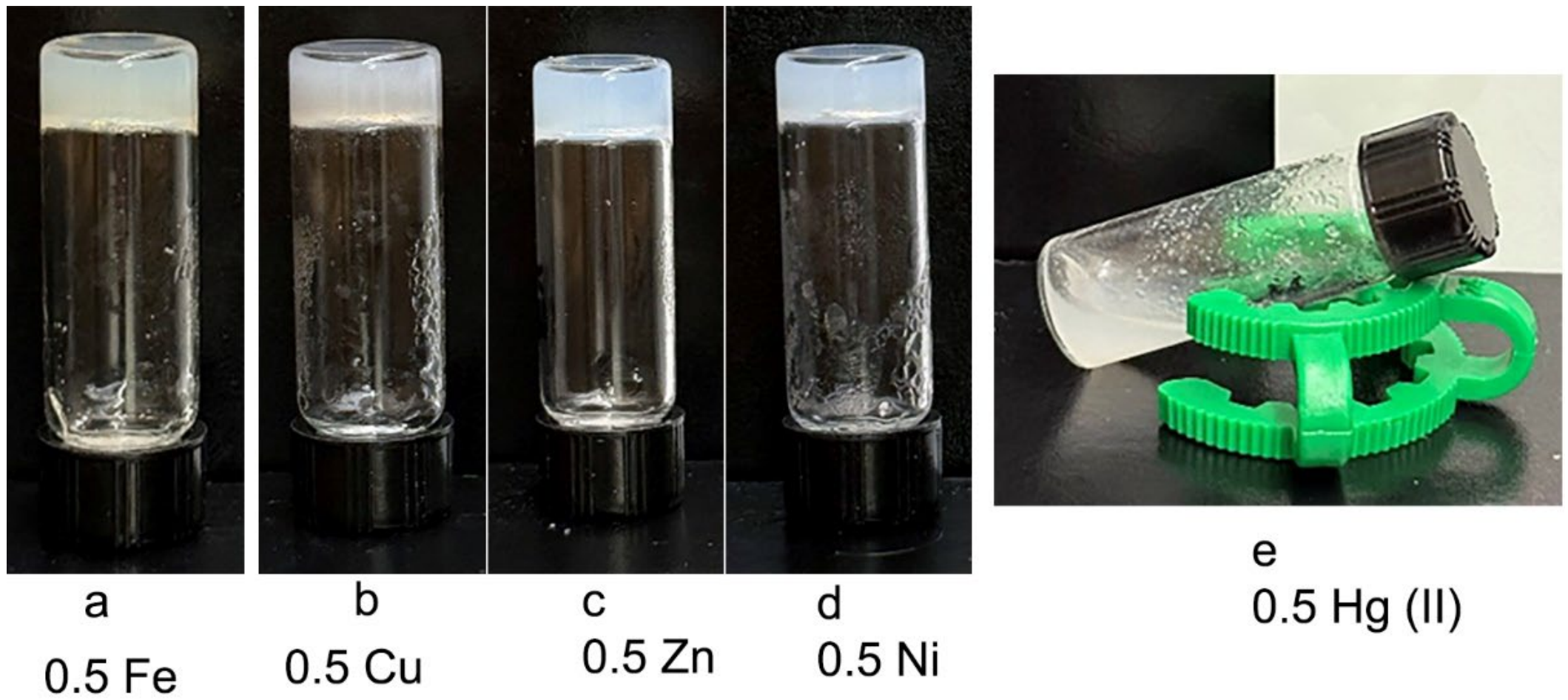

Figure S22. Compound **7** and 0.5 equivalent of metal ions, final solvent for all samples: DMSO 0.125 mL + 0.625 mL H_2O, 0.25 mL water+0.50 mL of DMSO/H_2O 1:3, the metal salts are a) $FeCl_2$, b) $Cu(OAc)_2 \cdot H_2O$, c) $Zn(OAc)_2 \cdot 2H_2O$, d) $NiCl_2 \cdot 6H_2O$, e) $Hg(OAc)_2$

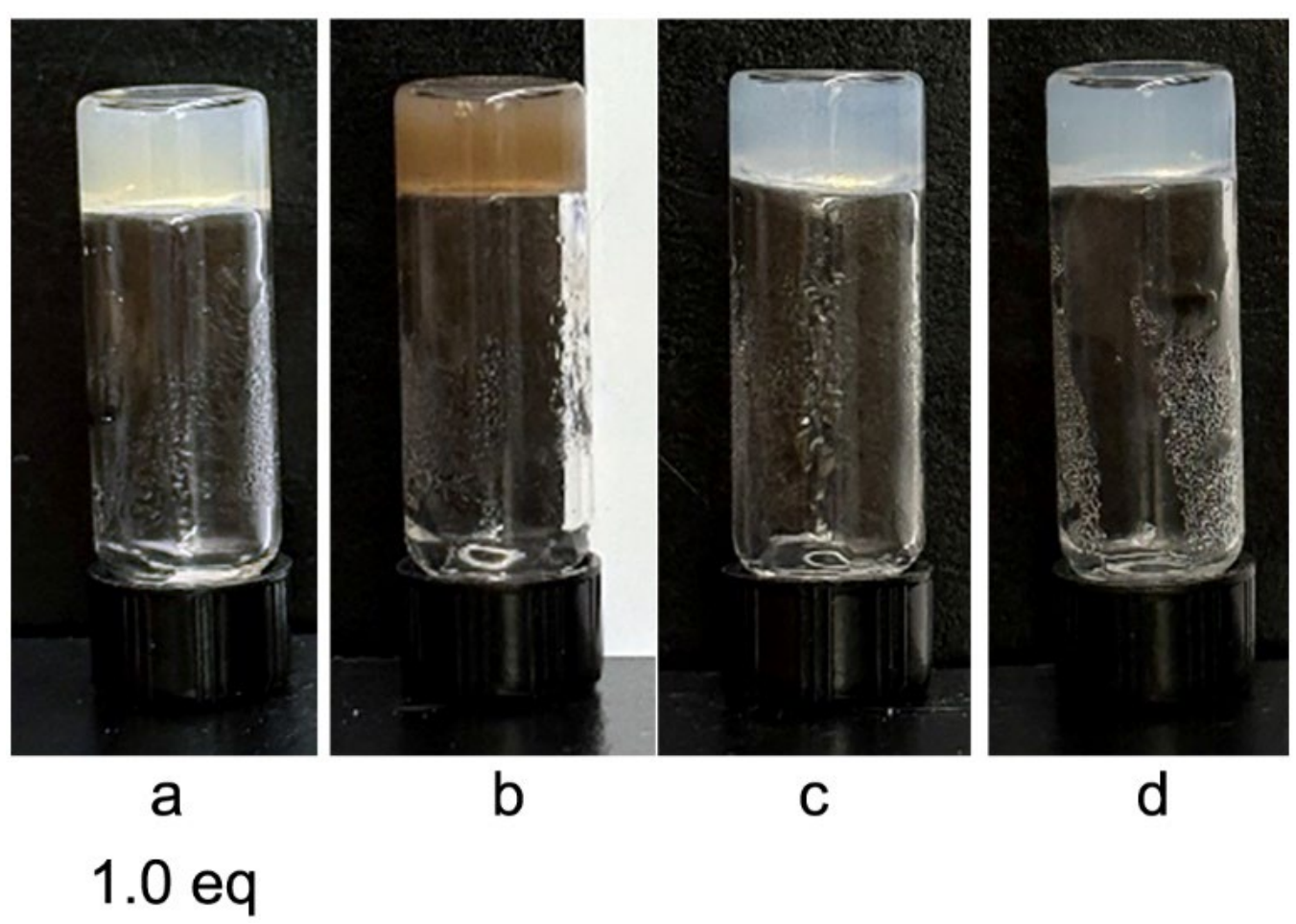

Figure S23. Compound **7** and 1.0 equivalent of metal ions, final solvent for all samples: DMSO 0.125 mL + 0.875 mL H_2O, 0.50 mL water+0.50 mL of DMSO/H_2O 1:3, the metal salts are a) $FeCl_2$, b) $Cu(OAc)_2 \cdot H_2O$, c) $Zn(OAc)_2 \cdot 2H_2O$, d) $NiCl_2 \cdot 6H_2O$.

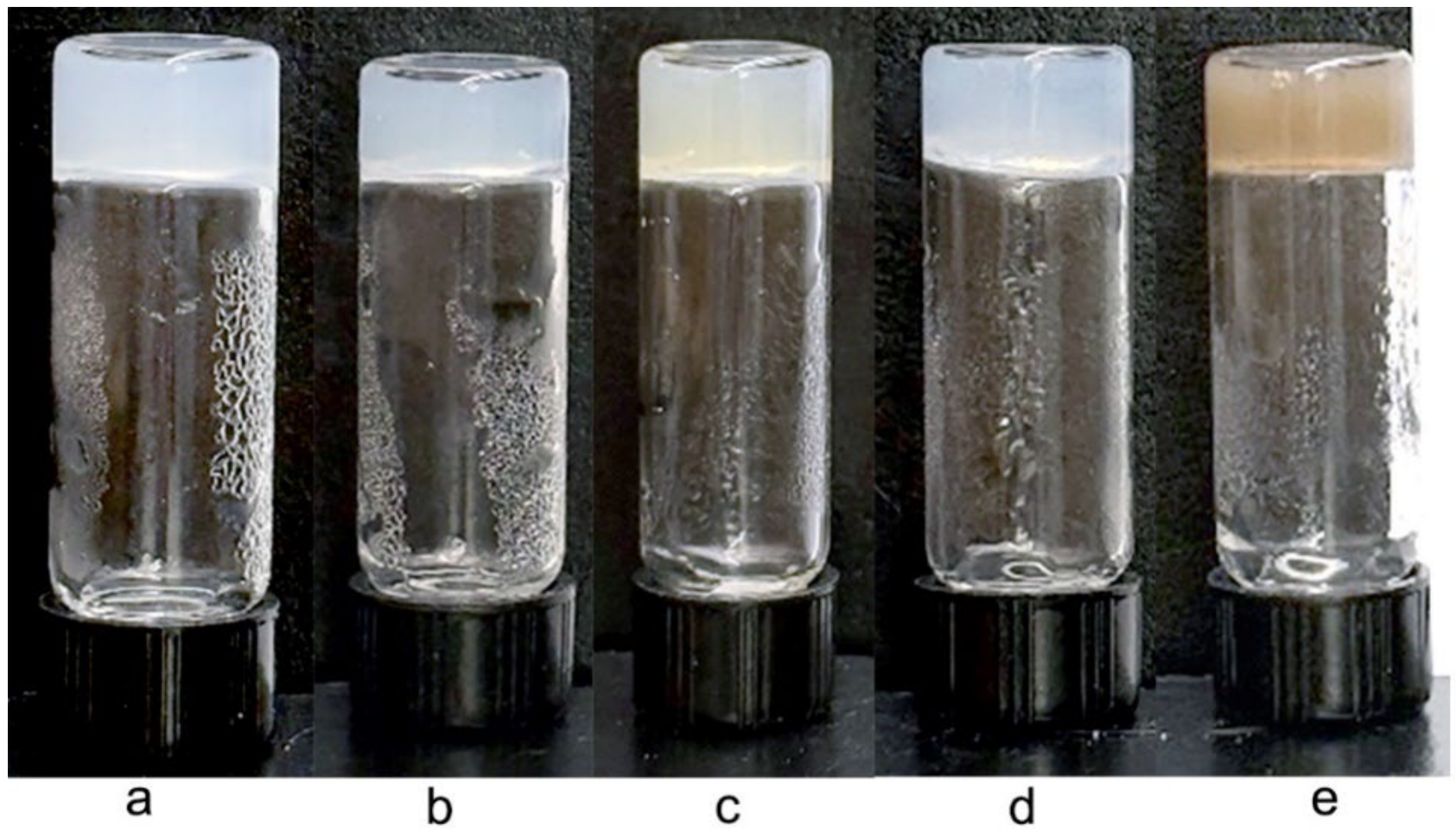

Figure S24. Gels formed by compound **7** in different solvents, (a) DMSO:H_2O (v/v 1:10), the gelator concentration is 1.8 mg/mL; (b)-(e) are compound **7** in the presence of one equivalent metal ions, the gelator concentrations are all 2.0 mg/mL in solvent DMSO:H_2O (v/v 1:7); (b) $NiCl_2$, (c) $FeCl_2$, (d) $Zn(OAc)_2$, (e) $Cu(OAc)_2$.

3. Swelling ratio of the compounds

2 mg of compound **7** was placed inside the syringe, and the volume was measured. The volume was about 10 mL. The same estimated volume was used for all compounds in the series to calculate the swelling ratio. Minimum gelation concentration (MGC) values were utilized to calculate the swelling ratio of the compounds. MGC was measured as mg/mL.

Table S5. Swelling ratio estimates of different compounds based on their MGC values.

MGC	G20.0	G10.0	G6.67	G5.0	G4.0	G3.3	G1.8	G1.6
Swelling Ratio	10	20	29.9	40	50	60	111	125

IV. Rheological properties of metallogels formed by compound 7

The rheological behavior of gels was analyzed using the Anton Paar MCR 302 rheometer equipped with RheoCompass software. The cone geometry featured a 25 mm Peltier plate, with a 0.1 mm gap for measurements. Experiments were conducted at a controlled temperature of 25.0 °C. Initially, an amplitude sweep was performed, varying the oscillation strain from 0.1% to 10%. Subsequently, a frequency sweep was performed over 0.1 to 100.0 rad/s at a constant strain of 0.5%. The results were presented as storage modulus (G′) and loss modulus (G″) as functions of angular frequency.

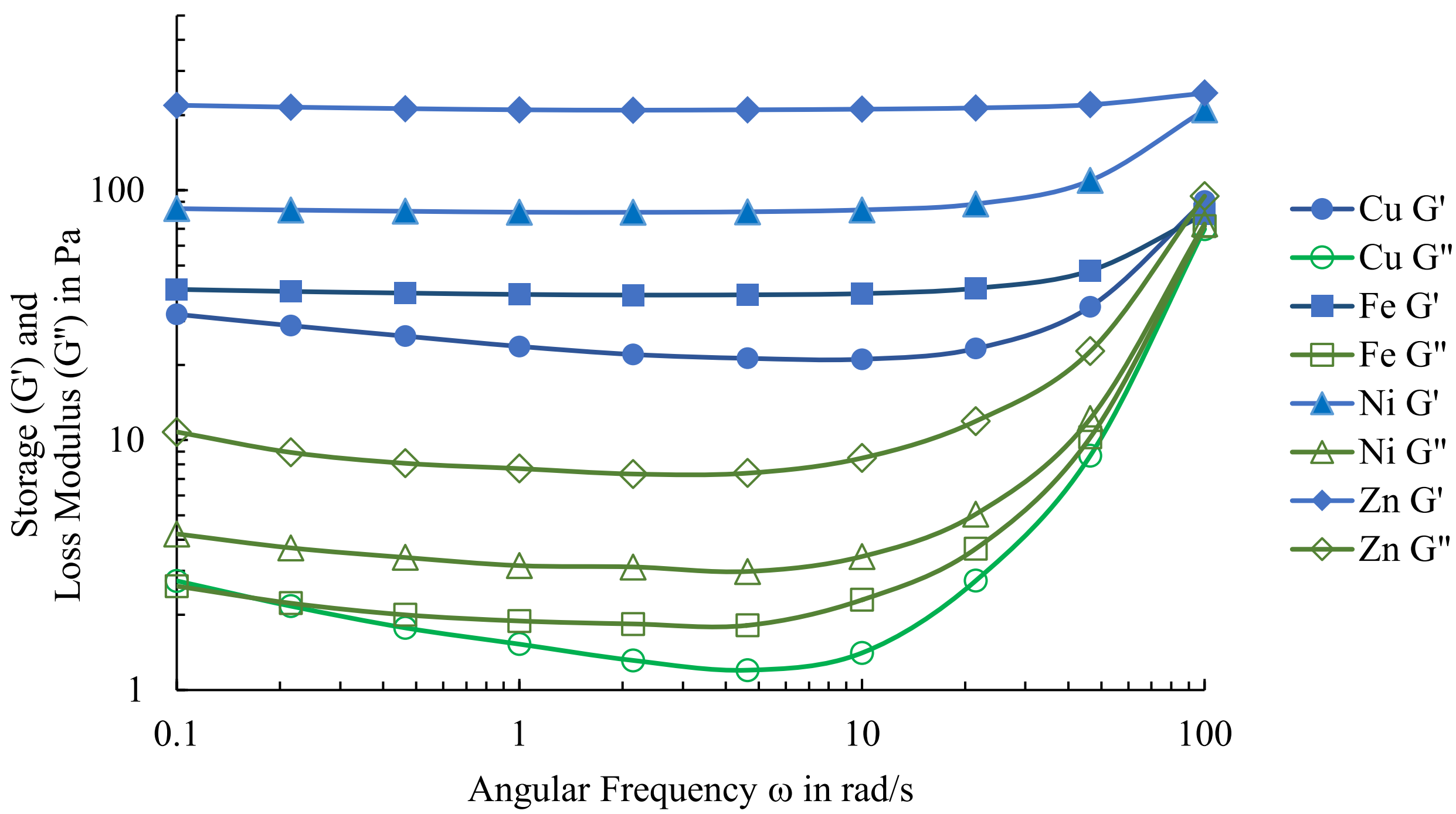

Figure S25. The rheological data for the metallogels are obtained using gels formed by compound **7** in DMSO:H_2O (1:4) at 4 mg/mL in the presence of one equiv of Cu, Fe, Ni, and Zn salts.

1. Rheology data of the gel formed by compound **7** in the presence of Cu (II).

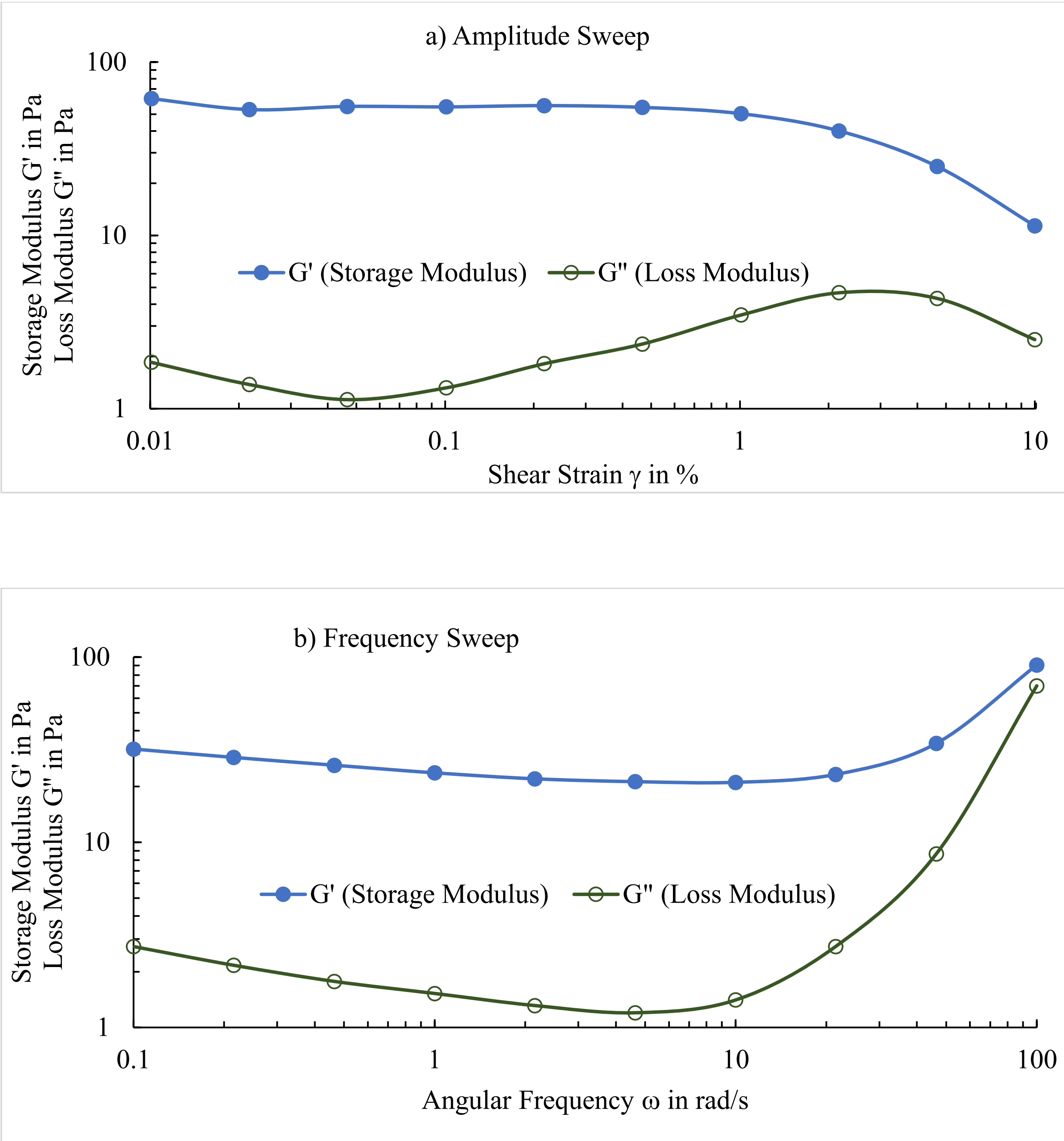

Figure S26. Amplitude sweep (a) and frequency sweep (b) for the gel formed by compound **7** the presence of Cu (II).

2. Rheology data of the gel formed by compound **7** in the presence of Fe (II).

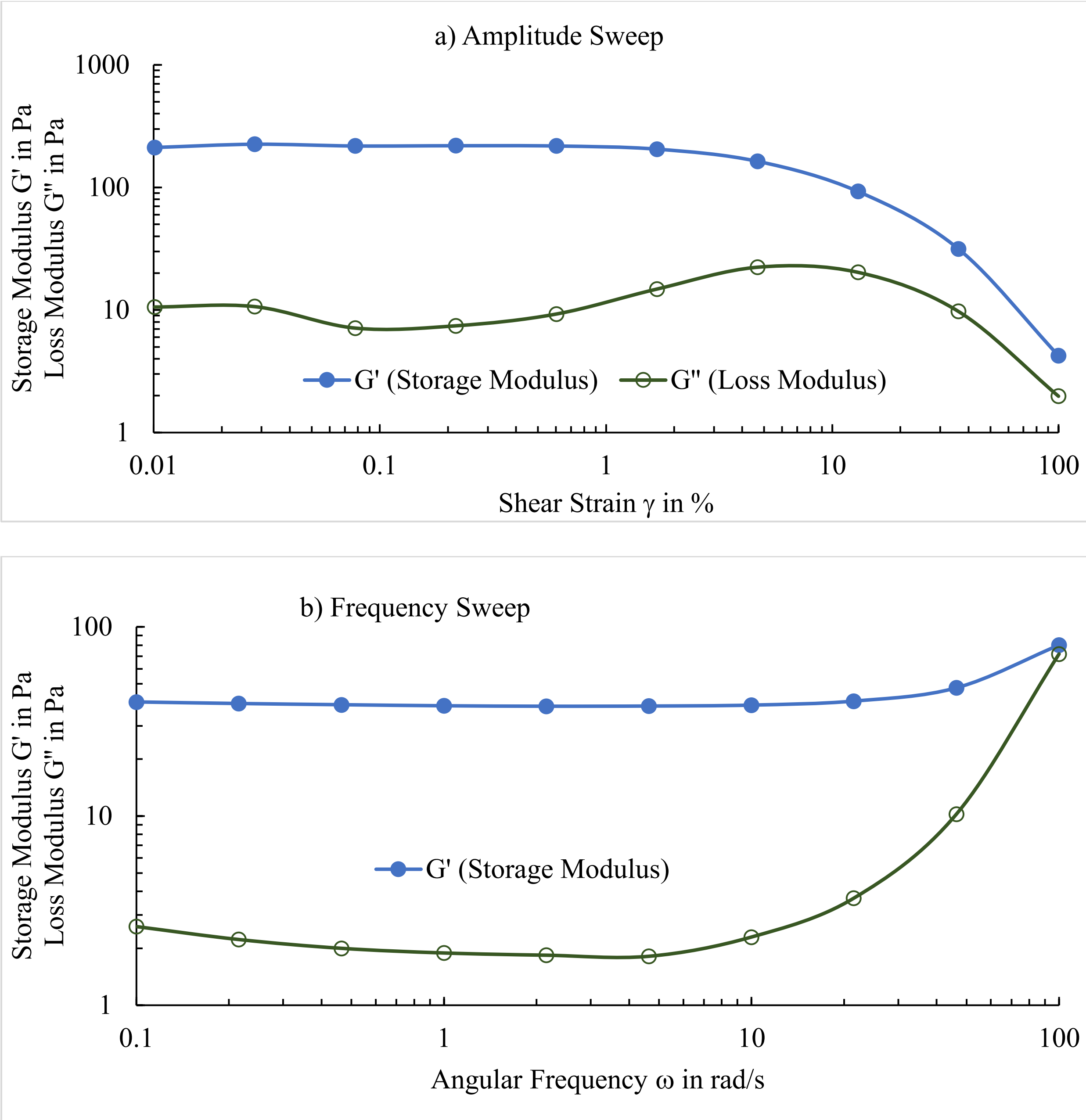

Figure S27. Amplitude sweep (a) and frequency sweep (b) for the gel formed by compound **7** the presence of Fe (II).

3. Rheology data of the gel formed by compound **7** in the presence of Ni (II).

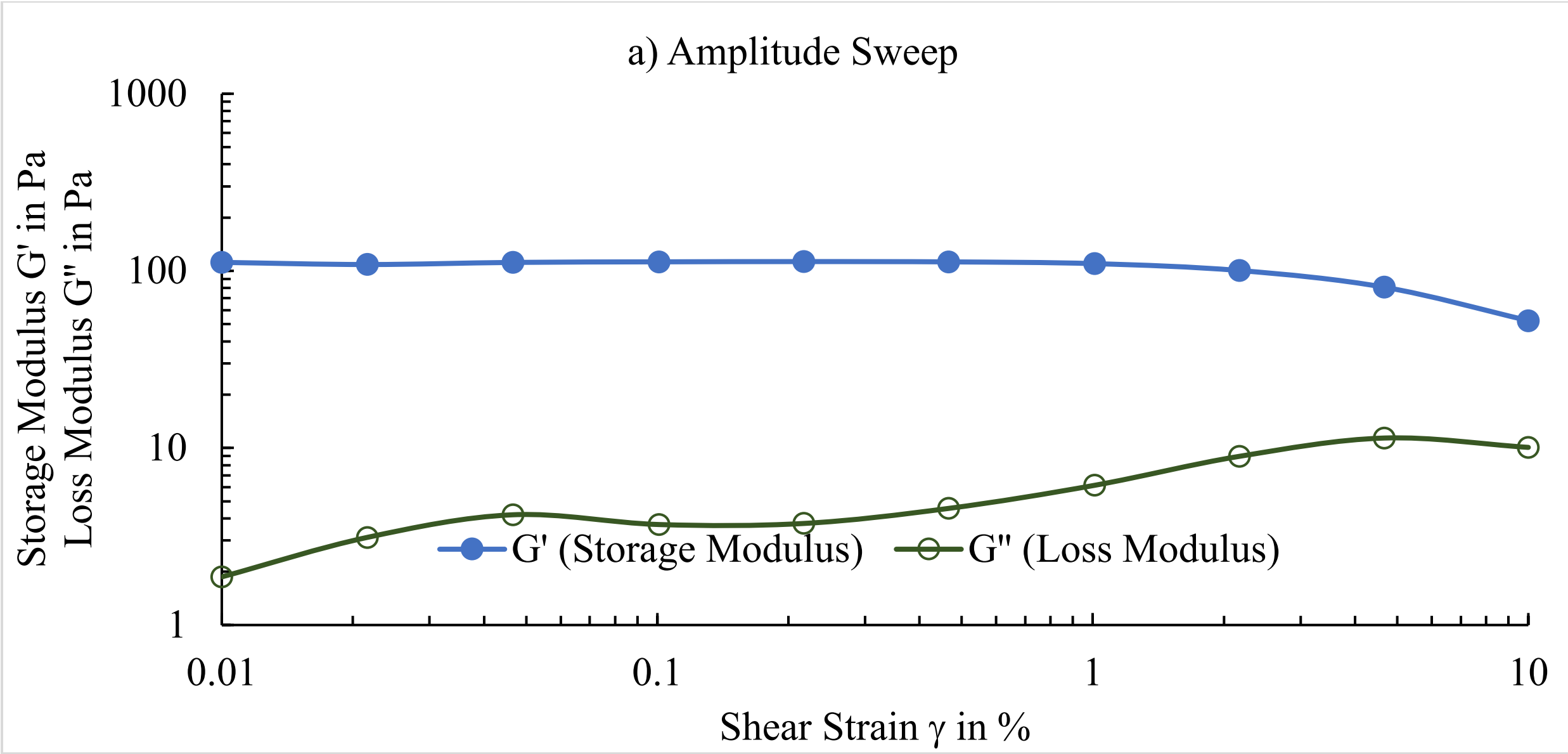

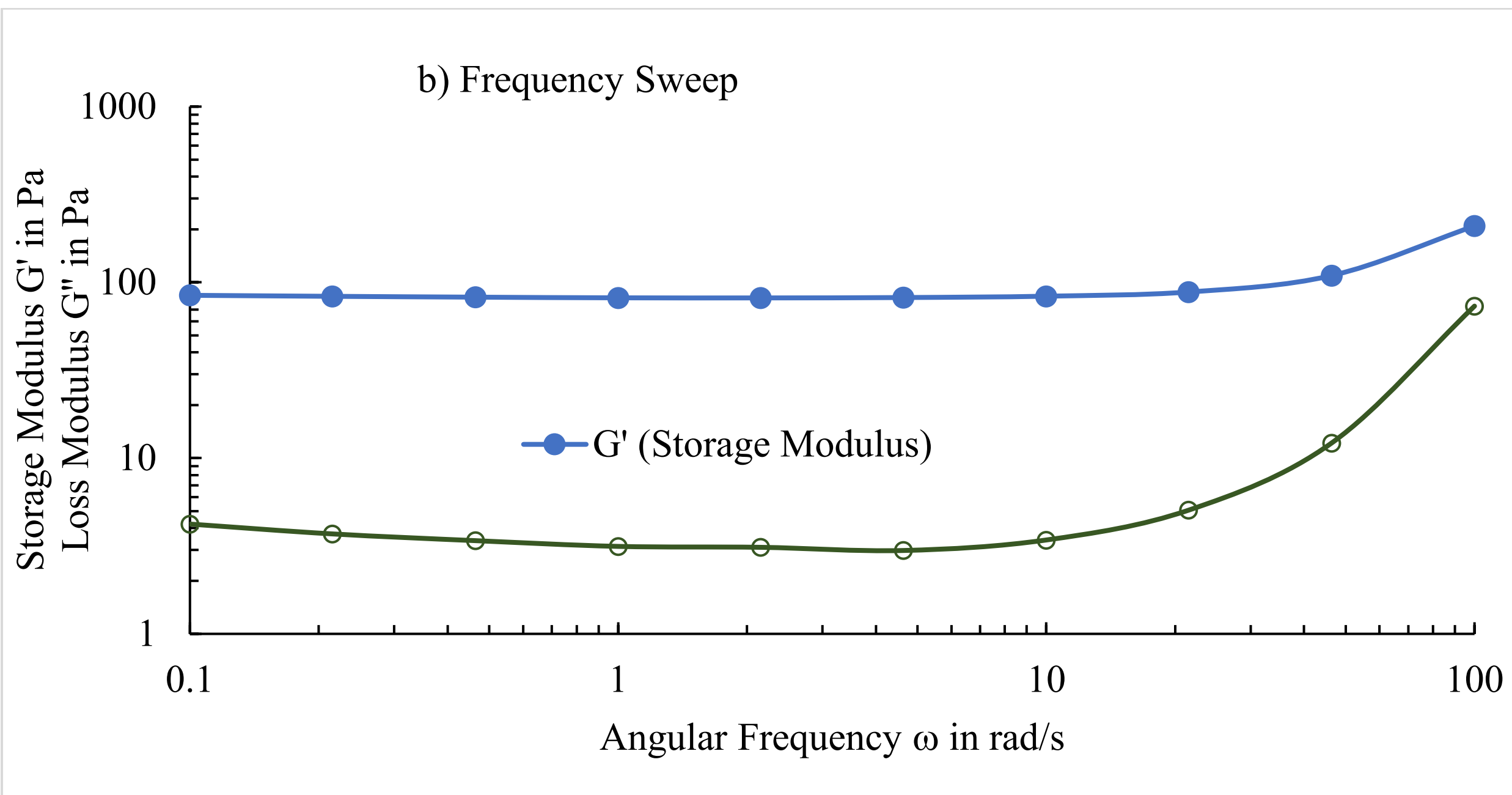

Figure S28. Amplitude sweep (a) and frequency sweep (b) for the gel formed by compound **7** the presence of Ni (II).

4. Rheology data of the gel formed by compound **7** in the presence of Zn (II).

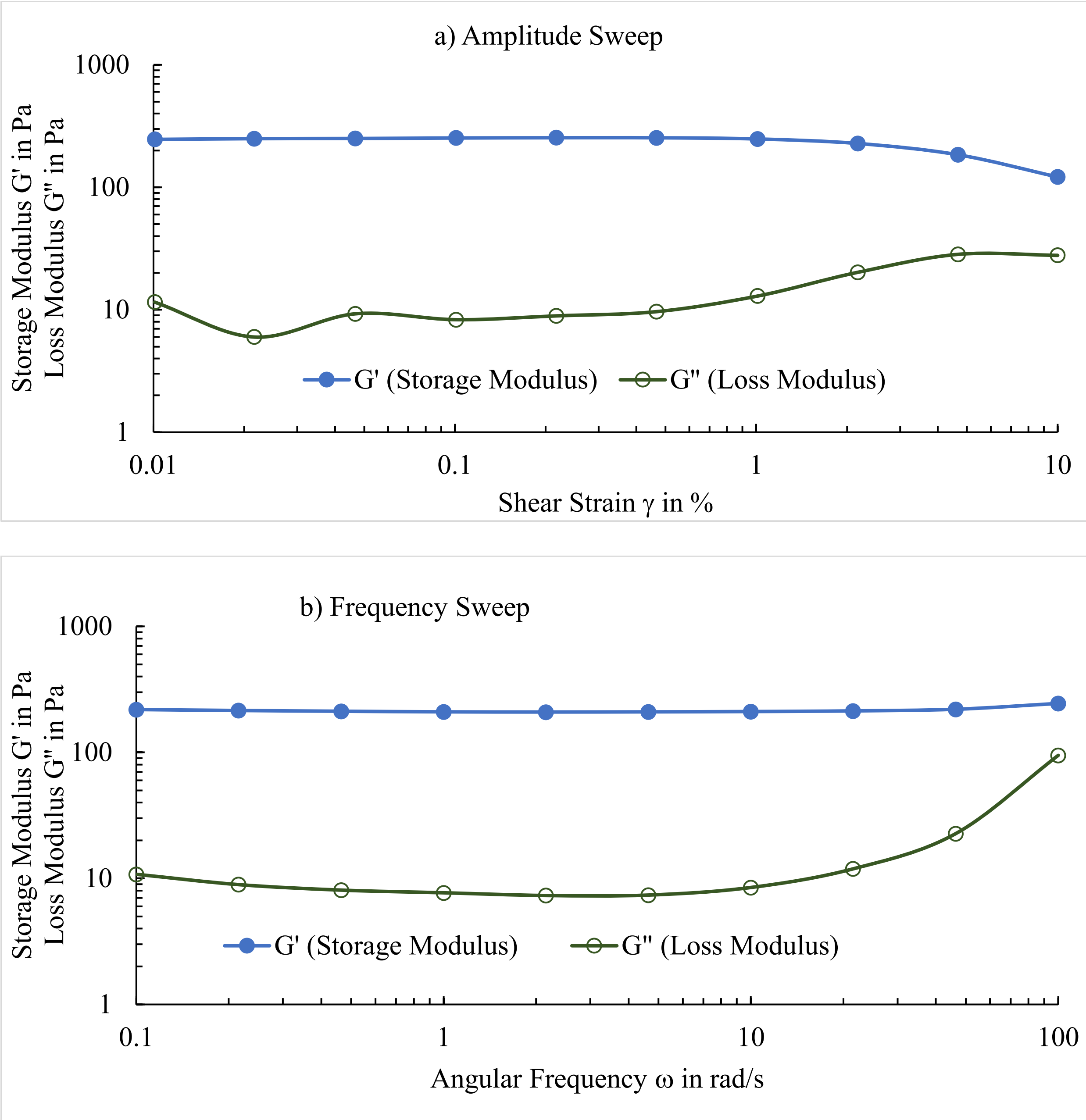

Figure S29. Amplitude sweep (a) and frequency sweep (b) for the gel formed by compound **7** in the presence of Zn (II).

Table S6. The G' and G", and the ratio of G'/G" for the gel prepared from compound **7** in the presence of Cu (II).

ω[rad/s]	G' (Storage Modulus)	G" (Loss Modulus)	G'/G"
100.00	90.657	69.931	1.30
46.40	34.206	8.6688	3.95
21.50	23.245	2.7401	8.48
10.00	21.07	1.4075	14.97
4.64	21.246	1.2007	17.69
2.15	22.012	1.3135	16.76
1.00	23.713	1.5261	15.54
0.46	26.068	1.7734	14.70
0.22	28.719	2.1672	13.25
0.10	31.875	2.7339	11.66
Average (G'/G") 0.1 to 10 [rad/s]			14.9

Table S7. The G' and G", and the ratio of G'/G" for the gel prepared from compound **7** in the presence of Fe (II).

ω [rad/s]	G' (Storage Modulus)	G" (Loss Modulus)	G'/G"
100.00	80.103	71.901	1.11
46.40	47.636	10.25	4.65
21.50	40.51	3.68	11.01
10.00	38.583	2.2941	16.82
4.64	38.15	1.8147	21.02
2.15	38.063	1.8385	20.70
1.00	38.28	1.8891	20.26
0.46	38.767	1.9991	19.39
0.22	39.344	2.2255	17.68
0.10	40.099	2.6043	15.40
Average (G'/G") 0.1 to 10 [rad/s]			18.8

Table S8. The G' and G", and the ratio of G'/G" for the gel prepared from compound **7** in the presence of Ni (II).

ω [rad/s]	G' (Storage Modulus)	G" (Loss Modulus)	G'/G"
100.00	209.34	73.267	2.86
46.40	109.32	12.2	8.96
21.50	88.128	5.0579	17.42
10.00	83.438	3.4216	24.39
4.64	81.976	2.9813	27.50
2.15	81.565	3.1079	26.24
1.00	81.664	3.1459	25.96
0.46	82.38	3.3913	24.29
0.22	83.29	3.7047	22.48
0.10	84.392	4.2137	20.03
Average (G'/G") 0.1 to 10 [rad/s]			24.4

Table S9. The G' and G", and the ratio of G'/G" for the gel prepared from compound **7** in the presence of Zn (II).

ω [rad/s]	G' (Storage Modulus)	G" (Loss Modulus)	G'/G"
100.00	244.58	94.681	2.58
46.40	219.91	22.678	9.70
21.50	213.45	11.909	17.92
10.00	210.96	8.4754	24.89
4.64	209.76	7.3766	28.44
2.15	209.1	7.3181	28.57
1.00	209.81	7.6915	27.28
0.46	212.1	8.0765	26.26
0.22	214.96	8.9319	24.07
0.10	218.77	10.783	20.29
Average (G'/G") 0.1 to 10 [rad/s]			25.7

V. Dye absorption studies using gels formed by compound 7

1. Toluidine Blue dye absorption study

The gel column was prepared using 3 mg of compound **7** in a 1 mL DMSO:H_2O (1:2) solution. Then the solution was heated and transferred to a gel column, where it was allowed to settle for 2 hours to form a gel. 1 mL of DI water was added on top of the gel column to flush DMSO from the gel. Then, 0.1 mL of Toluidine Blue (50 mg/L) was added to 1 mL of gel, and the mixture was allowed to diffuse for 30 minutes. The dye then passed through the gel, followed by the addition of 1 mL of water. The eluted liquid (1.4 mL) was collected and diluted to 2 mL. At the end of the experiment, the gel was stable enough to be inverted. Absorbance at λ_{max} (630 nm) of Toluidine Blue was measured using a calibration curve,[1] with about 90% of the dye from the initial solution.

UV absorption λ_{max} (630 nm) = 0.0221

From calibration curve: $y = 0.0302x - 0.0061$

$$0.0221 = 0.0302X - 0.0061 \rightarrow x = \frac{0.0221 + 0.0061}{0.0302} = 0.901\ \mu M$$

Amount of TB after column: 0.901 µM × (1 L/1000) × 305.82 × (1 L/1000 mL) × 2 mL =0.000551 mg

Original amount of TB added to column: 50.0 mg/L × (1 L/1000 mL) × 0.1 mL= 0.005 mg

Amount of TB removed from the solution: 0.00444 mg, 90%

VI. FTIR Spectra of compound 7 and its ethanol water gel

Gel was prepared at the concentration of 1.8 mg/mL at EtOH:H_2O (1:2). Bruker Alpha FTIR spectrometer was used for the measurement of the IR. A small piece of the gel/solid compound was placed on the disc of instrument and analyzed using OPUS software.

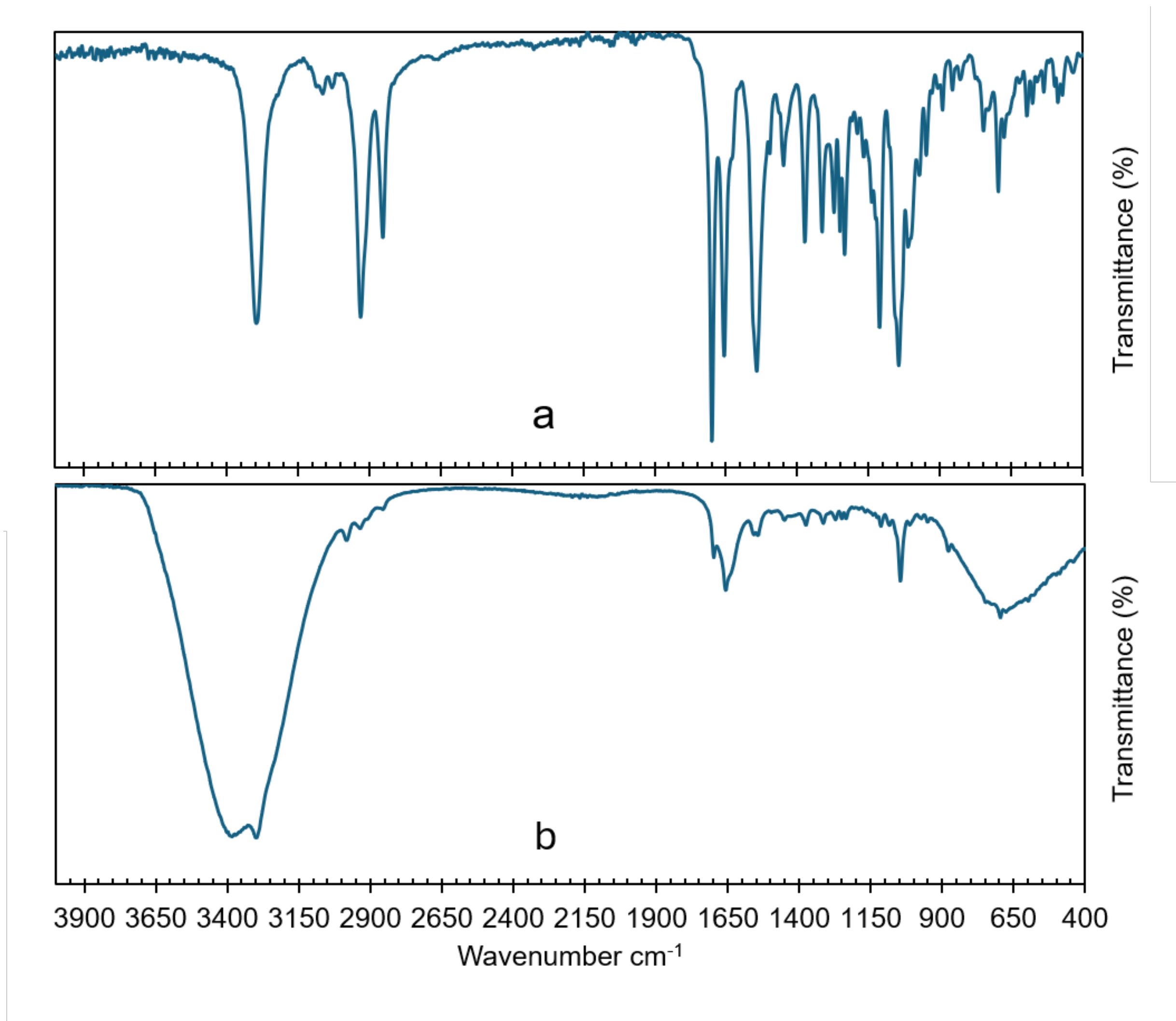

Figure 30. FT-IR spectrum of compound **7**. a) In solid form, b) in gel form of EtOH:H_2O (v/v 1:2) at 1.8 mg/mL.

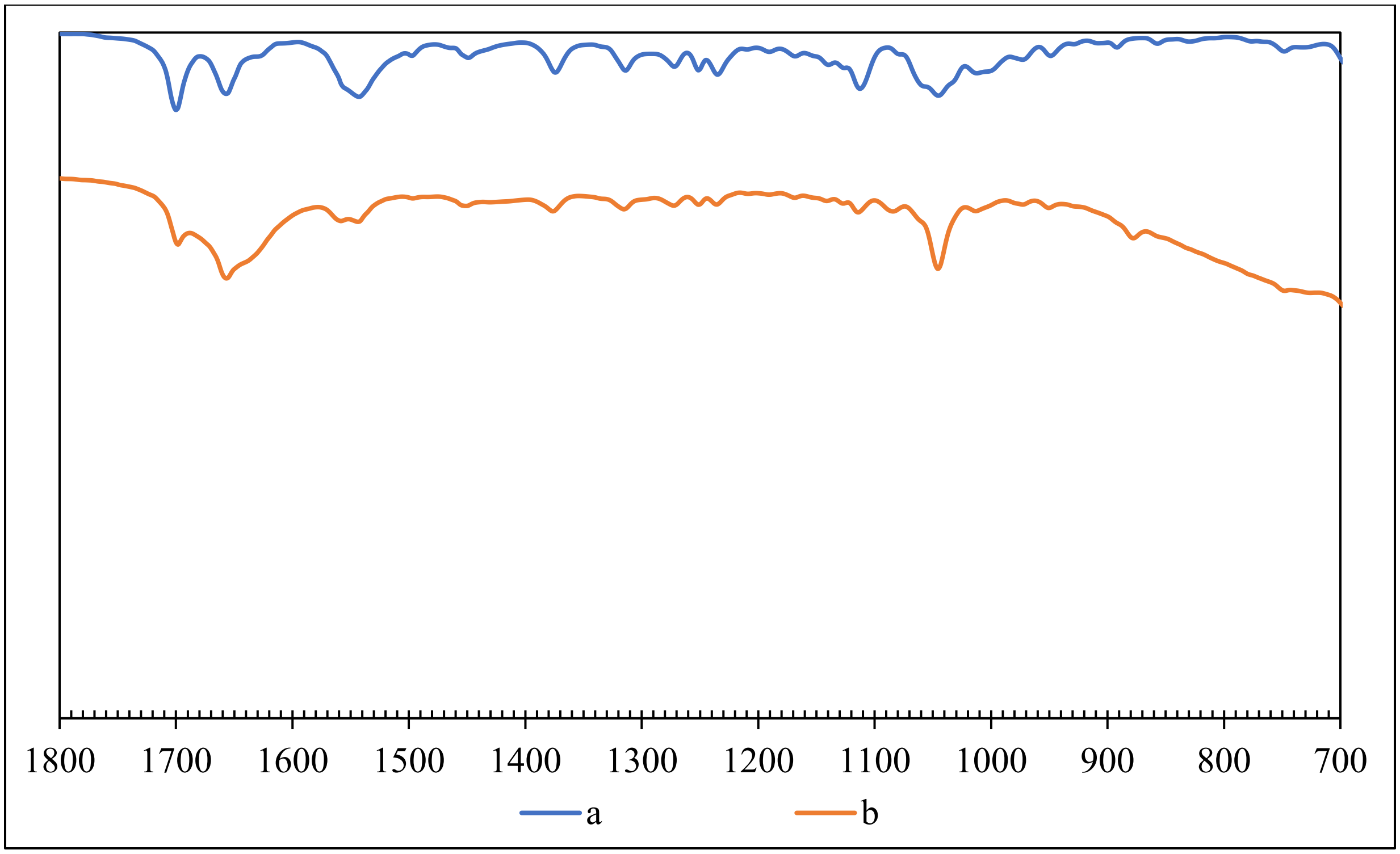

Figure 31. Overlay of IR spectra of compound **7**. a) compound **7** as solid; b) gel formed by compound **7** in EtOH:H_2O (1:2) at 1.8 mg/mL.

VII. Chemiluminescence experiments

The chemiluminescence of the metallogels was analyzed using the Varioskan LUX with SkanIt Software. After adding the peroxide to the metallogel/Luminol solution, the mixture was shaken for 5 seconds at 600 rpm. After, there was a five-second pause before chemiluminescent readings were taken. The luminescent spectrum was taken starting at 270 nm to 670 nm with a measurement time of 0.1 second. This was followed by a luminescence reading with a

measurement time of 1 sec. The luminescent spectrum and luminescence reading were then repeated twice. Averages were taken and displayed in the graphs and charts below.

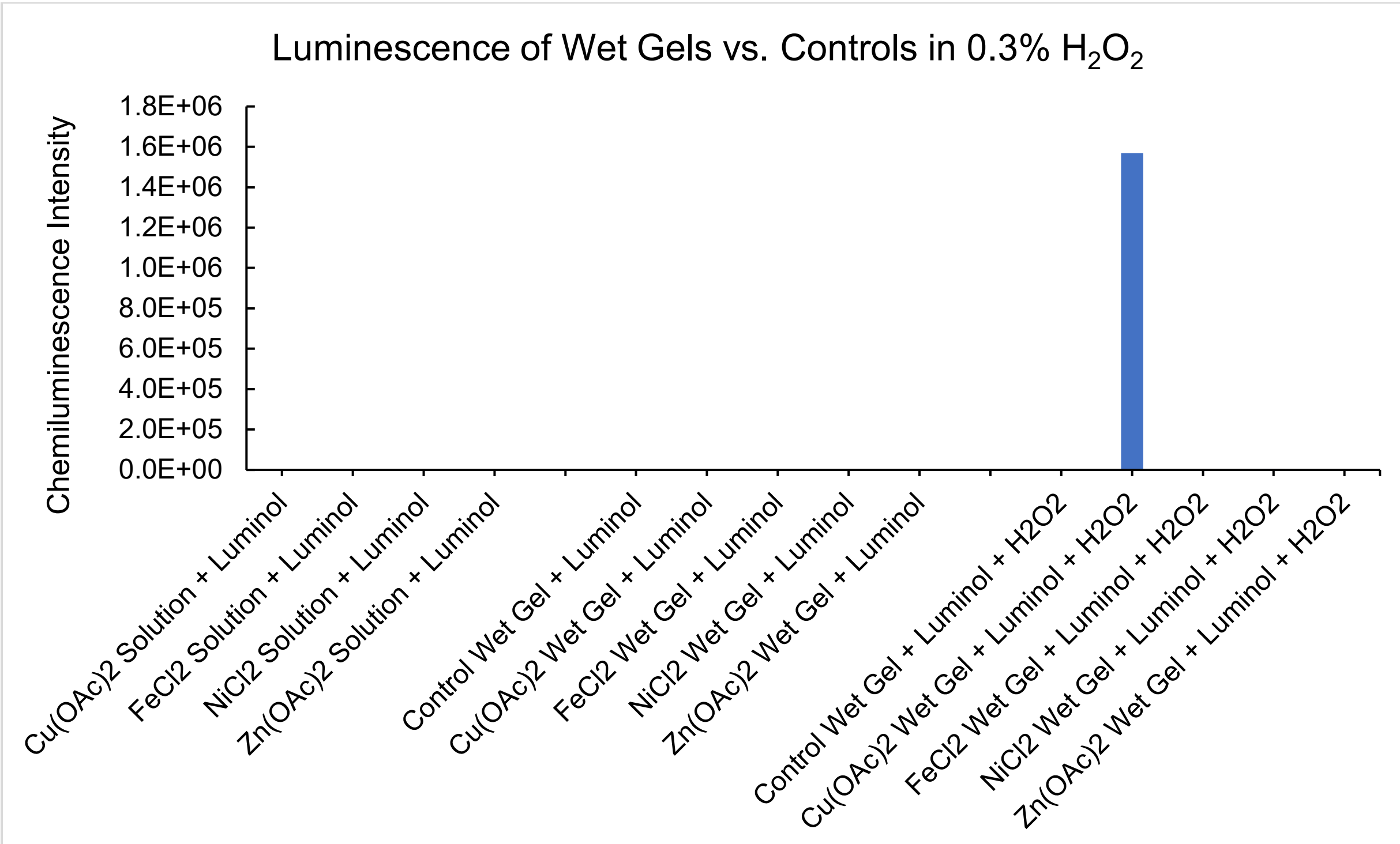

Figure S32. Chemiluminescence intensities of luminol with metal-hydrogels with different metal ions. Control: Metallogel (100 µL 4.0 mg/mL in 1:4 DMSO:MilliQ water) + Luminol (100 µL of 2.0 mg/mL solution in 10.0 mM NaOH). Wet gel: Metallogel (100 µL 4.0 mg/mL in 1:4 DMSO:MilliQ water) + Luminol (100 µL of 2.0 mg/mL solution in 10.0 mM NaOH) + H_2O_2 (100 µL of 0.3%).

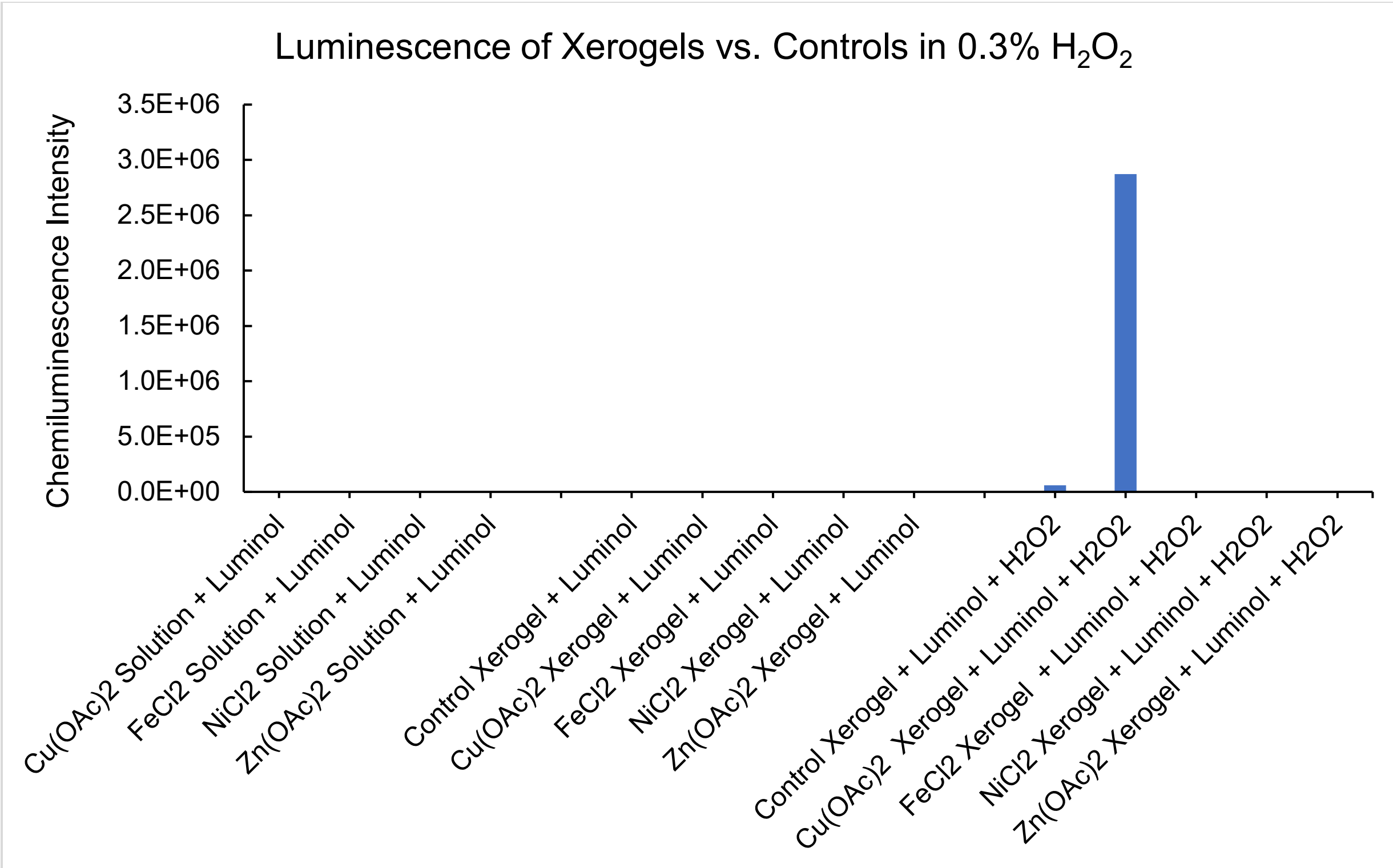

Figure S33. Chemiluminescence intensities of luminol with metal-hydrogels with different metal ions. Control: Metallogel (100 µL 4.0 mg/mL in 1:4 DMSO:MilliQ water) + Luminol (100 µL of 2.0 mg/mL solution in 10.0 mM NaOH). Xerogel: Metallogel (100 µL 4.0 mg/mL in 1:4 DMSO:MilliQ water) + Luminol (100 µL of 2.0 mg/mL solution in 10.0 mM NaOH) + H_2O_2 (100 µL of 0.3%).

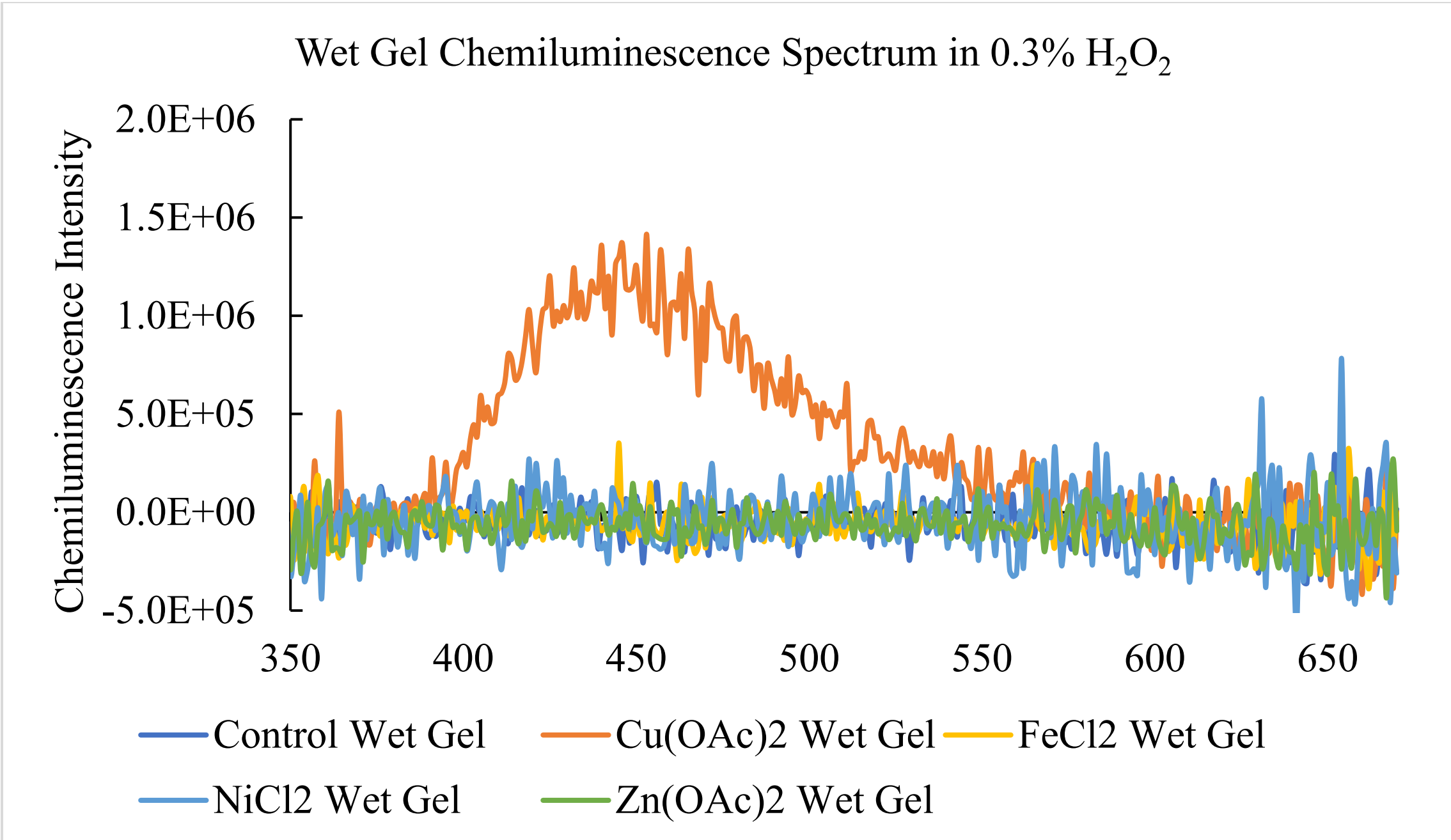

Figure S34. Chemiluminescence spectra of wet gels with 0.3% H_2O_2

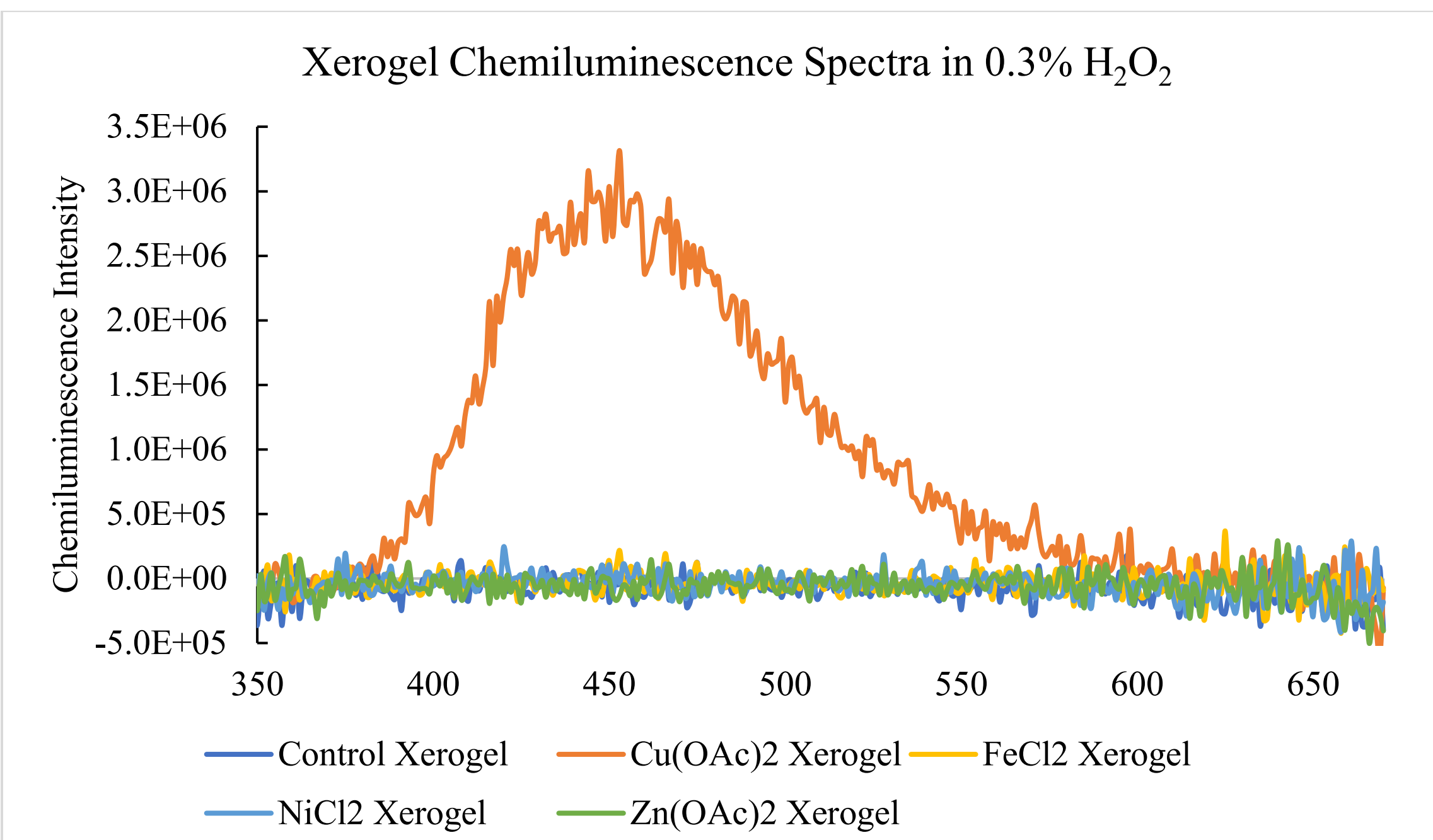

Figure S35. Chemiluminescence spectra of wet gels with 0.3% H_2O_2

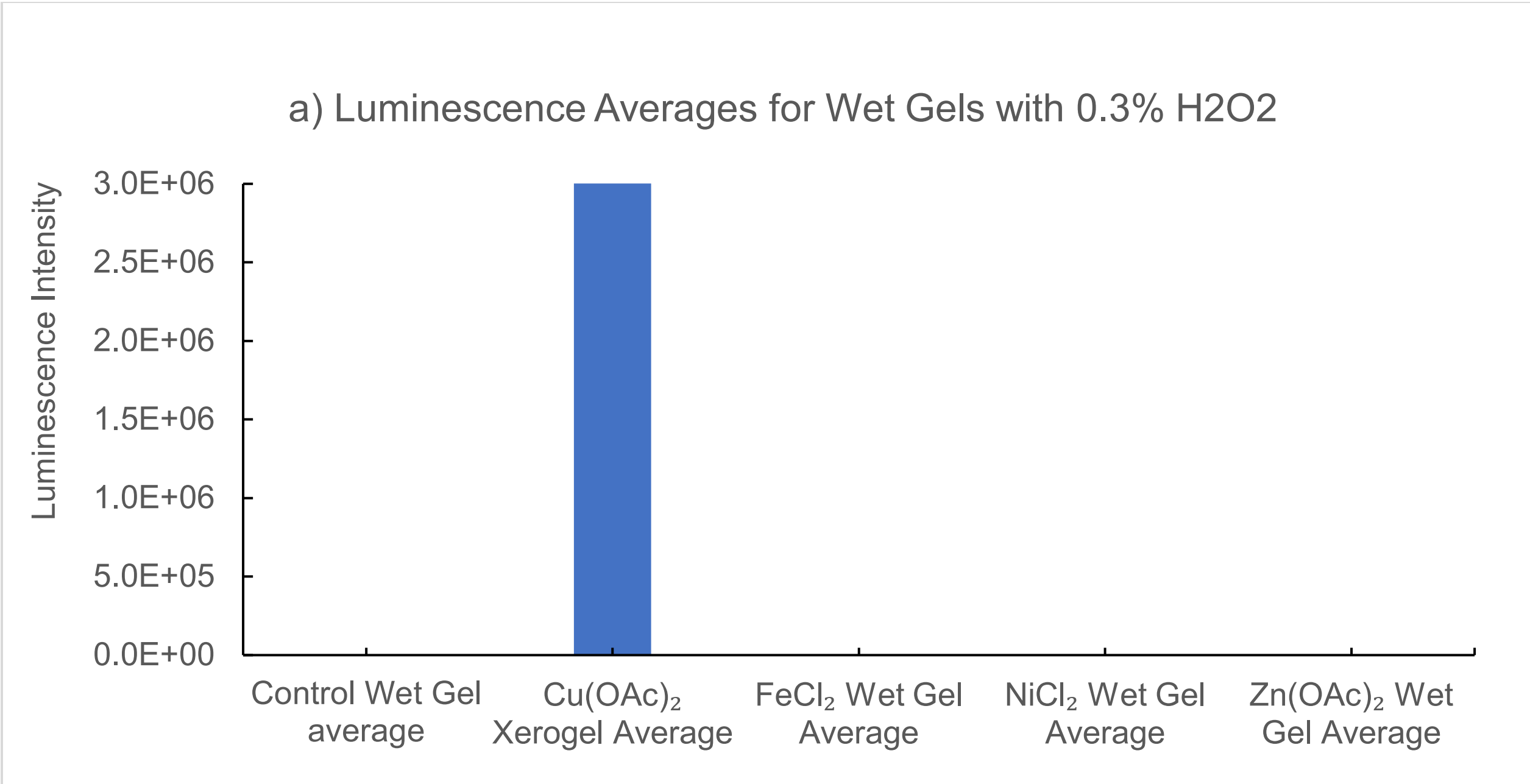

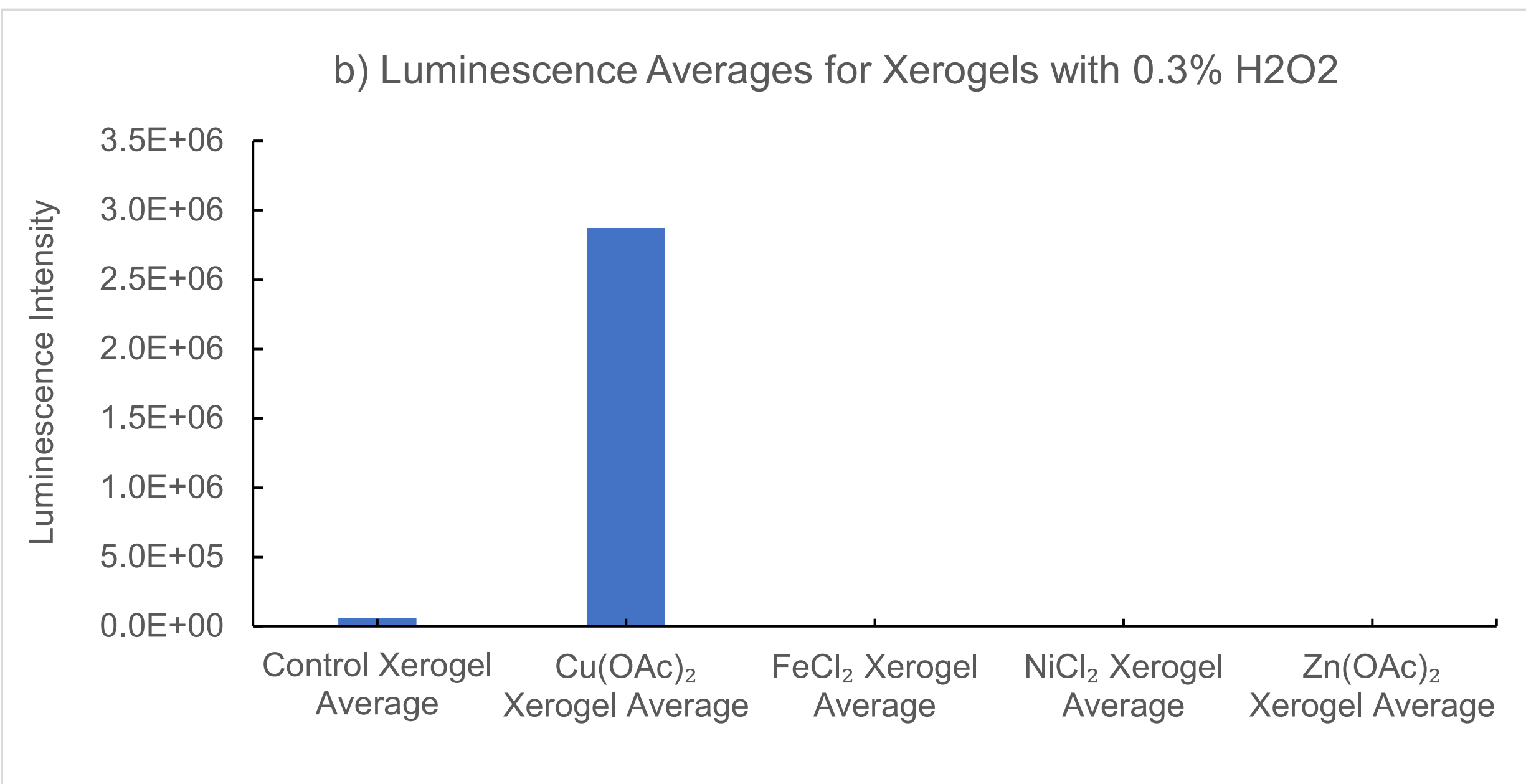

Figure S36 (a) Chemiluminescence intensities of luminol in the presence of metallogels with different metal ions. Metal organogels (100 μL of 4.0 mg/mL in 1:4 DMSO:MilliQ water) + Luminol (100 μL of 2.0 mg/mL solution in 10.0 mM NaOH) + H_2O_2 (100 μL of 0.3%). (b) Chemiluminescence intensities of luminol in the presence of zero metal-organic gels with different metal ions.

Table S10. Chemiluminescence data for Compound **7** Wet Gels

Wet Gel in 0.3% H_2O_2					
Trial	Chemiluminescence Intensity				
	Control + Luminol + H_2O_2	$Cu(OAc)_2$ + Luminol + H_2O_2	Fe + Luminol + H_2O_2	Ni + Luminol + H_2O_2	Zn + Luminol + H_2O_2
1	1001	1755000	48.02	4399	191.6
2	927.6	1507000	26.35	4505	226.3
3	819.6	1445000	18.31	4531	123.1
Average	916.1	1569000	30.89	4478	180.3
SD%	9.96	10.45	49.74	1.56	29.12

Table S11. Chemiluminescence data for Compound **7** Xerogels

Xerogel in 0.3% H_2O_2					
Trial	Chemiluminescence Intensity				
	Control + Luminol + H_2O_2	$Cu(OAc)_2$ + Luminol + H_2O_2	$FeCl_2$ + Luminol + H_2O_2	$NiCl_2$ + Luminol + H_2O_2	$Zn(OAc)_2$ + Luminol + H_2O_2
1	53800	2953000	8269	4223	1416
2	57050	2935000	6807	4325	1363
3	63100	2731000	6058	4048	1676
Average	57983	2873000	7045	4199	1485
SD%	8.14	4.29	15.96	3.34	11.28

References.

(1) Aryal, P.; Adhikari, S. B.; Duffney, A.; Wang, G. Sugar isoxazole based hydrogelators and their applications as reusable hydrogels for dye removal. *New Journal of Chemistry* **2023**, *47* (37), 17224-17228, 10.1039/D3NJ03038E. DOI: 10.1039/D3NJ03038E.
